# Deep learning enables robust assessment and selection of human blastocysts after in vitro fertilization

**DOI:** 10.1038/s41746-019-0096-y

**Published:** 2019-04-04

**Authors:** Pegah Khosravi, Ehsan Kazemi, Qiansheng Zhan, Jonas E. Malmsten, Marco Toschi, Pantelis Zisimopoulos, Alexandros Sigaras, Stuart Lavery, Lee A. D. Cooper, Cristina Hickman, Marcos Meseguer, Zev Rosenwaks, Olivier Elemento, Nikica Zaninovic, Iman Hajirasouliha

**Affiliations:** 1 Institute for Computational Biomedicine, Department of Physiology and Biophysics, Weill Cornell Medicine of Cornell University, New York, NY USA; 2 Caryl and Israel Englander Institute for Precision Medicine, The Meyer Cancer Center, Weill Cornell Medicine, New York, NY USA; 3 Yale Institute for Network Science, Yale University, New Haven, CT USA; 4 The Ronald O. Perelman and Claudia Cohen Center for Reproductive Medicine, Weill Cornell Medicine, New York, NY USA; 5 Institute of Reproduction and Developmental Biology, Imperial College, Hammersmith Campus, London, UK; 6 Department of Biomedical Informatics, Emory University School of Medicine, Atlanta, GA USA; 7 Instituto Valenciano de Infertilidad, Universidad de Valencia, Valencia, Spain; 8 WorldQuant Initiative for Quantitative Prediction, Weill Cornell Medicine, New York, NY USA

**Keywords:** Image processing, Machine learning

## Abstract

Visual morphology assessment is routinely used for evaluating of embryo quality and selecting human blastocysts for transfer after in vitro fertilization (IVF). However, the assessment produces different results between embryologists and as a result, the success rate of IVF remains low. To overcome uncertainties in embryo quality, multiple embryos are often implanted resulting in undesired multiple pregnancies and complications. Unlike in other imaging fields, human embryology and IVF have not yet leveraged artificial intelligence (AI) for unbiased, automated embryo assessment. We postulated that an AI approach trained on thousands of embryos can reliably predict embryo quality without human intervention. We implemented an AI approach based on deep neural networks (DNNs) to select highest quality embryos using a large collection of human embryo time-lapse images (about 50,000 images) from a high-volume fertility center in the United States. We developed a framework (STORK) based on Google’s Inception model. STORK predicts blastocyst quality with an AUC of >0.98 and generalizes well to images from other clinics outside the US and outperforms individual embryologists. Using clinical data for 2182 embryos, we created a decision tree to integrate embryo quality and patient age to identify scenarios associated with pregnancy likelihood. Our analysis shows that the chance of pregnancy based on individual embryos varies from 13.8% (age ≥41 and poor-quality) to 66.3% (age <37 and good-quality) depending on automated blastocyst quality assessment and patient age. In conclusion, our AI-driven approach provides a reproducible way to assess embryo quality and uncovers new, potentially personalized strategies to select embryos.

## Introduction

Infertility remains an unremitting reproductive issue that affects about 186 million people worldwide.^1^ In the United States, infertility affects ~8% of women of child-bearing age.^2^

In vitro fertilization (IVF) is one of the most common treatments for infertility. IVF involves ovarian stimulation followed by the retrieval of multiple oocytes, fertilization, and embryo culture for 1–6 days in controlled environmental conditions. Although IVF and embryo-transfer technologies have improved considerably over the past 30 years, the efficacy of IVF remains relatively low.^3^

Conventional embryo evaluation involves manual grading of human embryos at the blastocyst stage (embryo on day 5) based on morphological analysis by skilled embryologists.^4^ While this selection method is used universally in clinical practice, the evaluation of an embryo based on a static image represents a crude, subjective evaluation of embryo quality, which is incomplete as well as time-consuming.^5–7^

Moreover, there continues to be a tendency for inconsistent blastocyst classification, often associated with different grading systems among medical centers. Indeed, attempts to establish a universal grading and selection system have thus far failed to catch on.^8^

Improving the ability to select the single best embryo with the highest implantation potential would increase pregnancy rates as well as minimize the chance of multiple pregnancies due to the transfer of multiple embryos.^9^ Time-lapse imaging (TLI)^10^ is an emerging technology that allows continuous observation of embryo development without removing embryos from controlled and stable incubator conditions.^11^ However, even though TLI represents a step toward more objective embryo evaluation, the inter- and intra-evaluator variation among embryologists using conventional morphological grading and/or TLI annotations is well documented.^12–14^

There are various efficient machine learning methods, which due to their relatively better performances in various fields of research are utilized for embryo classification. Two recent studies have attempted to use some of these approaches for embryo quality analysis, with varying degrees of success^15,16^ on a limited bovine and mammalian oocytes data, using AI- and random forest (RF)-based classification, respectively. Their results showed 76.4% (test set = 73 embryos) and 75% (test set = 56 embryos) accuracy for discretization of bovine embryo grades and mammalian oocyte grades, respectively. Furthermore, a few previously published approaches have focused on classifying human blastocyst quality based on specific features, such as the inner cell mass (ICM) area, trophectoderm (TE) area, zona pellucida (ZP) thickness, and blastocyst area and radius separately.^9,17^ In particular, Filho et al.^17^ presented a semi-automatic grading system of human embryos. The authors showed that classifiers can have different accuracies for each embryo component (blastocyst extension, ICM, and TE). Their results indicated various accuracy ranges from 67 to 92% for the embryo extension, from 67 to 82% for the ICM, and from 53 to 92% for TE detection; 92% was the highest accuracy achieved across a 73-embryo test set.^17^ Although these methods achieved reasonable accuracy in assessing human embryo quality, they require advanced embryological expertize and several preprocessing steps, and do not scale to large datasets.

**Fig. 1:**
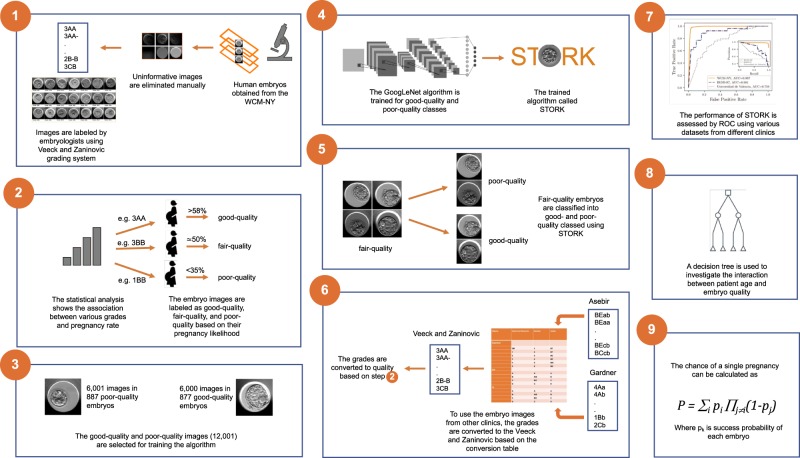
The STORK flowchart: This flowchart illustrates the design and assessment of STORK. First, Human embryo images are provided from the embryology lab and labeled by embryologists as good-quality or poor-quality based on their pregnancy likelihood. Then, the labels and clinical information from the extracted images are integrated, and the Inception-V1 algorithm is trained for good-quality and poor-quality classes. Furthermore, STORK is evaluated by a blind test set to assess its performance in predicting embryo quality. Finally, the CHAID decision tree is used to investigate the interaction between patient age and embryo quality

Deep learning algorithms, in particular convolutional neural networks (CNNs), have recently been used to address a number of medical-imaging problems, such as detection of diabetic retinopathy,^18^ skin lesions,^19^ and diagnosing disease.^20^ They have become the technique of choice in computer vision and they are the most successful type of models for image analysis. Unlike regular neural networks, CNNs contain neurons arranged in three dimensions (i.e., width, height, depth). Recently, deep architectures of CNNs such as Inception^21^ and ResNet^22^ have dramatically increased the progress rate of deep learning methods in image classification.^23^ In this paper, we sought to use deep learning to accurately predict the quality of human blastocysts and help select the best single embryo for transfer (Fig. 1).

## Results

### Deep neural network achieves a highly accurate classification of embryo images

**Fig. 2:**
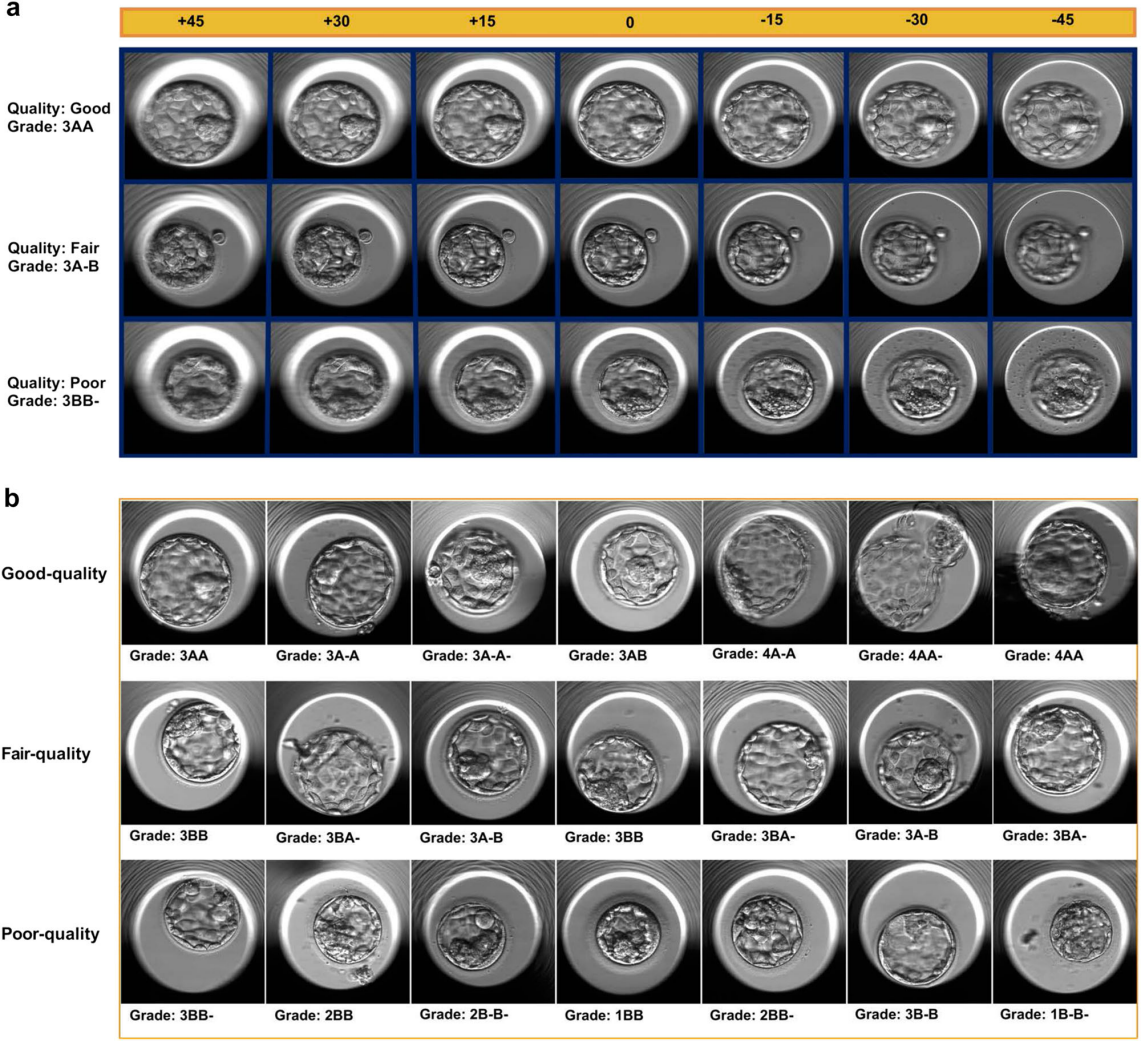
Embryologists’ evaluation: **a** This figure shows three examples of Veeck and Zaninovich grades and their corresponding quality labels across seven focal depths. **b** Embryologists evaluate embryo quality using an internal scoring system and subsequently classify them into three major groups (good-quality, fair-quality, poor-quality) based on the pregnancy rate

We used time-lapse images from 10,148 human embryos, obtained from the Center for Reproductive Medicine at Weill Cornell Medicine to train and validate our DNN. The 10,148 embryos (WCM-NY dataset) were classified into three major quality groups, good-quality (*n* = 1345 embryos), fair-quality (*n* = 4062 embryos), and poor-quality (*n* = 4741 embryos) (Fig. 2a, b) based on their assigned grades (see Methods). We obtained time-lapse images from each of the embryos, each consisting of several time points, seven focal depths per time point (Fig. 2a, b) and 500 × 500 pixels black and white images per focal depth (+45, +30, +15, 0, −15, −30, and −45). Upon preprocessing and removal of images with readability issues (e.g., those with a dark background) and random selection of a balanced set of images (see Methods), we were left with a total of 12,001 images from up to seven focal depths: 6000 images in 877 good-quality embryos, and 6001 images in 887 poor-quality embryos.

We then trained an Inception-V1 DNN–based algorithm using the two quality groups at both ends of the spectrum, i.e., good-quality and poor-quality. The Inception-V1 architecture is a transfer learning algorithm, where we initially performed fine-tuning of the parameters for all of the layers. We used 50,000 steps for training the DNN and subsequently evaluated the performance of our DNN (called STORK) using a randomly selected independent test set with 964 good-quality images from 141 embryos and 966 poor-quality images from 142 embryos. Our results showed that the trained algorithm was able to identify good-quality and poor-quality images with 96.94% accuracy (1871 correct predictions out of 1930 images).

To measure the accuracy of STORK for individual embryos, we used a simple voting system across multiple image focal depths. If the majority of images from the same embryo were predicted to be of good-quality, then the final quality of the embryo was considered good. For a small number of cases in which the number of good-quality and poor-quality images was equal (e.g., three good-quality and three poor-quality for six focal depths), we used STORK’s output probability scores to break the tie. At the embryo level, we obtained 97.53% accuracy with 276 correct predictions out of 283 embryos.

**Fig. 3:**
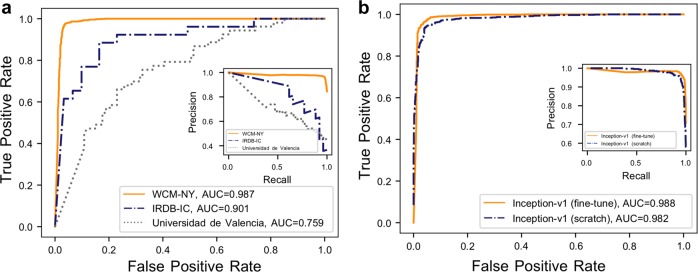
Deep neural network results: **a** Inception-V1 (fine-tuning the parameters for all layers) results for three datasets. **b** Inception-V1 via two different training methods (fine-tuning the parameters for all layers and training from scratch) in good-quality and poor-quality embryo quality discrimination dataset. WCM-NY: data from the Center for Reproductive Medicine and Infertility at Weill Cornell Medicine of New York; IRDB-IC: data from the Institute of Reproduction and Developmental Biology of Imperial College; Universidad de Valencia: data from the Institute Valenciano de Infertilidad, Universidad de Valencia

At the image level, we observed an average area under the curve (AUC) of 0.987 (Fig. 3a) on the blind test set. We also found that training an Inception-V1 model without parameter fine-tuning did not affect performance (accuracy; Fig. 3b). This observation is in agreement with previous studies using these deep learning techniques.^20,24,25^

**Fig. 4:**
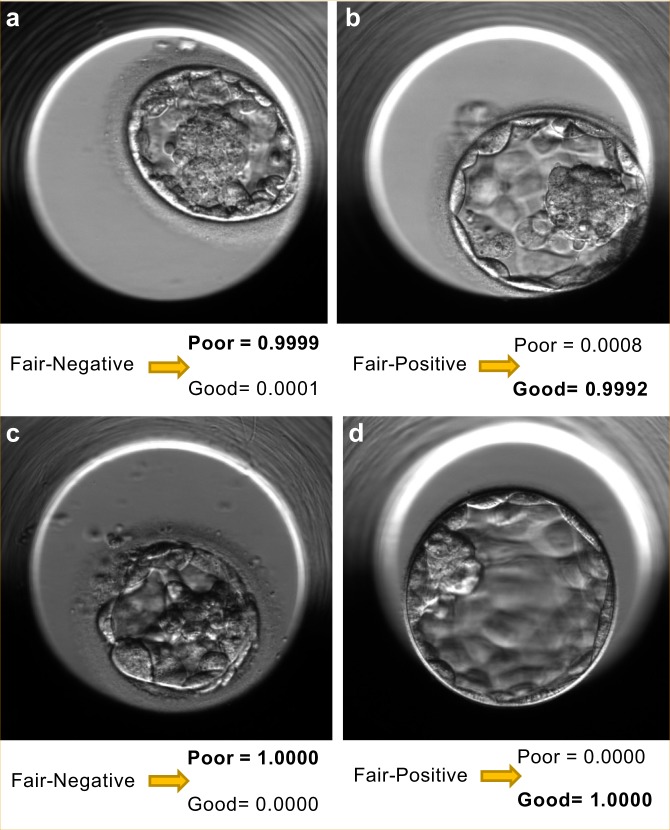
STORK vs. embryologists classification: STORK classifies the fair-quality images into existing good-quality and poor-quality classes. For example, panels “**a**” and “**b**” are labeled 3A-B (fair-quality) according to the Veeck and Zaninovic grading system, while STORK classified them as poor-quality and good-quality, respectively. Also, panels “**c**” and “**d**” are both labeled 3BB (fair-quality). However, the algorithm correctly classified panel “**c**” as poor-quality and panel “**d**” as good-quality. As the figure shows, the outcome in the embryos in “**b**” and “**d**” is positive live birth, whereas it is negative live birth in “**a**” and “**c**”

We also found that STORK classified the fair-quality embryo (intermediate group, Figs. 2 and 4) images (4480 images from 640 embryos) as 82% good-quality (526 embryos) and 18% poor-quality (114 embryos), respectively. As Inception-V1 was trained for good-quality and poor-quality classes with different pregnancy probabilities (an ~58% and 35% chance of pregnancy for good-quality and poor-quality classes, respectively), we wondered if STORK nonetheless produced relevant predictions (association between embryo quality and pregnancy rate) within the fair-quality class. A closer look showed that embryos with fair-quality images that were classified as poor-quality by STORK had a lower likelihood of positive live birth (50.9%) as compared to those classified as good-quality (61.4% positive live birth; *p* < 0.05 by the two-tailed Fisher’s test). Note that STORK alone cannot estimate the pregnancy rate. However, it can detect the association between embryo quality and pregnancy rate based on morphological classification.

In addition, we found that fair-quality embryos predicted to be good-quality by STORK came from younger patients (33.9 years old on average) than those predicted to be poor-quality (34.25 years old on average). Interestingly, these numbers are similar to the age of patients with good-quality and poor-quality embryos: 33.86 and 34.72 years old on average, respectively. This suggests that STORK finds sufficient structure within embryos classified as fair-quality to make clinically relevant predictions (Fig. 4).

### STORK is robust when applied to datasets from other clinics

To evaluate STORK’s robustness, we tested its performance by using additional datasets of embryo images obtained from two other IVF centers, Universidad de Valencia and IRDB-IC, comprising 127 (74 good-quality, 53 poor-quality) and 87 (61 good-quality, 26 poor-quality) embryos, respectively (Supplementary Table 2). Our experimental results (See Fig. 3a) demonstrate that although the scoring systems used for these centers are different from the system used to train our model, STORK can successfully identify and register score variations and robustly discriminate between them, with an AUC of 0.90 and 0.76 for the IRDB-IC and Universidad de Valencia and (Fig. 3a), respectively. Lower concordance of the classification results (by STORK) for the Universidad de Valencia dataset could be related to different grading systems used by that clinic. The images of Universidad de Valencia dataset are labeled using Asebir^26^ while IRDB-IC is labeled using the Gardner system.^27^ The Veeck and Zaninovic grading system is a slightly modified version of the Gardner system (Supplementary Table 4).

### STORK outperforms individual embryologists for embryo selection

It is well known that embryo scoring frequently varies among embryologists,^28^ mainly due to the subjectivity of the scoring process and different interpretations of embryo quality. We, therefore, sought to create a small but robust benchmark embryo dataset that would represent the consensus of several embryologists. We asked five embryologists from three different clinics to provide scores for each of 394 embryos generated in different labs (Supplementary Table 6). Note that these images were not used in the training phase of our algorithm. The embryo images were scored using the Gardner scoring system^27^ and then mapped onto our simplified three groups (good-quality, fair-quality, and poor-quality; see Supplementary Table 4 for the mapping method).

As expected, we found a low level of agreement among the embryologists (Supplementary Fig. 1b), with only 89 embryos out of the 394 classified as the same quality by all five embryologists (Supplementary Fig. 1a). Therefore, to create a larger and more accurate gold standard dataset, we used an embryologist majority voting procedure (i.e., the quality of each image was determined by the score given by at least three out of the five embryologists) to classify 239 images (32 good-quality and 207 poor-quality).

**Fig. 5:**
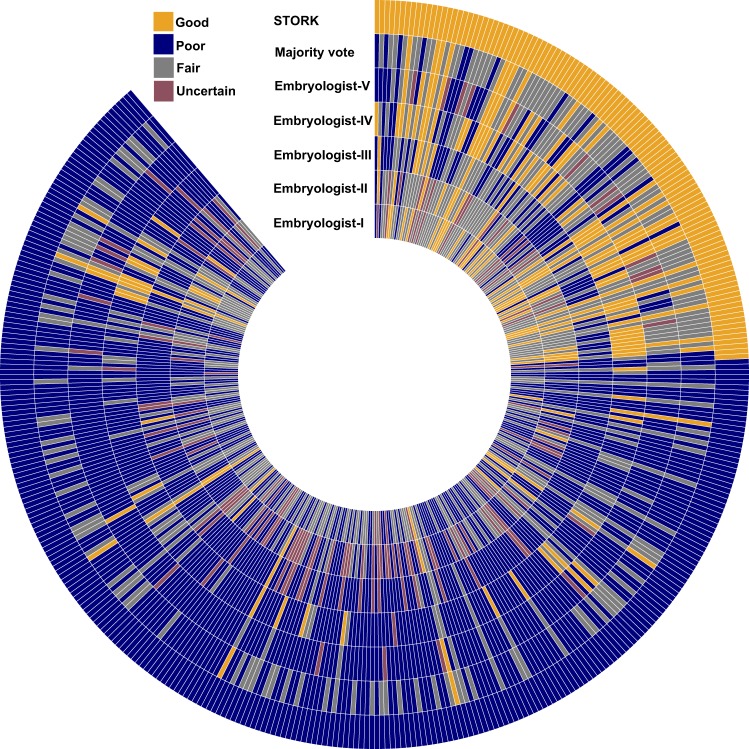
Assessment comparison of STORK with five embryologists: This circular heatmap demonstrates the prediction of STORK and five embryologists in the labeling of the same images from 394 embryos. STORK outputs good and poor grades. The heatmap compares STORK’s result with the majority vote results from all of the embryologists for 239 embryos in which the majority (i.e., at least three out of five embryologists) gives good or poor. The embryologists assess the embryos quality using Gardner grading system. Then, they convert the grades to the three different quality scores as good-quality (orange), fair-quality (gray), and poor-quality (navy) based on the pregnancy rate. Also, for a few embryos, the embryologist uses “?” signs (e.g. 3A?), which refer to the low certainty (red) as they are not sure about the exact label. The heatmap illustrates the result of STORK, Majority vote, Embryologist-V, Embryologist-IV, Embryologist-III, Embryologist-II, and Embryologist-I from the outer circle to the inner ones. Orange: embryos with good-quality; navy: embryos with poor-quality; gray: embryos with fair-quality; red: embryos that are not labeled due to uncertainty

When we applied STORK to these 239 images, we found that it predicted the embryologist majority vote with precision of 95.7% (Cohen’s kappa = 0.63). In comparison, STORK agreed with each individual embryologist as follows: 0.69, 0.54, 0.25, 0.62, and 0.54 Cohen’s kappa score. These results indicate that STORK may outperform individual embryologists when assessing embryo image quality (Fig. 5).

### A decision tree predicts likelihood of successful pregnancy based on embryo quality and clinical parameters

It is known that other factors, besides embryo quality, such as patient age, the patient’s genetic background, clinical diagnosis, and treatment-related characteristics, can affect pregnancy outcome.^29,30^ As embryo quality is one of the most important of these factors, the ultimate aim of any embryo assessment approach is to identify embryos that have the highest implantation potential resulting in live birth.^27,31,32^ However, embryo quality alone is not enough to accurately determine the pregnancy probability (see Supplementary Method 1, Supplementary Method 2 and Supplementary Fig. 2).

Therefore, in this section we present an alternative method for predicting successful pregnancy probability based on a state-of-the-art decision tree method that integrates clinical information and embryo quality. We wondered if we could assess the successful pregnancy rate by using a combination of embryo quality and patient age, as age is one of the most important clinical variables. For this purpose, we used a hierarchical decision tree method known as chi-squared automatic interaction detection (CHAID) algorithm.^33^

**Fig. 6:**
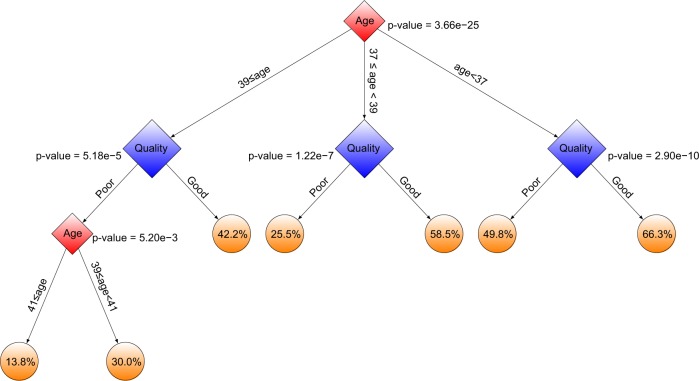
Interactions between age and embryo quality: The decision tree shows the interactions between IVF patient age and embryo quality using CHAID

We designed a CHAID^34,35^ decision tree using 2182 embryos from the WCM-NY database (Supplementary Table 6) with available clinical information and pregnancy outcome results (Fig. 6). We then investigated the interaction between patient age (consisting of seven classes: ≤30, 31–32, 33–34, 35–36, 37–38, 39–40, and ≥41) (Supplementary Fig. 3a) and embryo quality (consisting of two classes: good-quality and poor-quality). The fully de-identified data consists of a very diverse population of patients (Supplementary Fig. 3b). The effect on live birth outcome is demonstrated in (Supplementary Fig. 3c). The CHAID algorithm can project interactions between variables and non-linear effects, which are generally missed by traditional statistical techniques. CHAID builds a tree to determine how variables can explain an outcome in a statistically meaningful way.^34,35^ CHAID uses chi-squared statistics for identification of optimal multi-way splits, and identifies a set of characteristics (e.g., patient age and embryo quality) that best differentiates individuals based on a categorical outcome (here, live birth) and creates exhaustive and mutually exclusive subgroups of individuals. It chooses the best partition on the basis of statistical significance and uses Bonferroni-adjusted *p*-values to determine significance with a predetermined minimum size of end nodes. We used a 1% Bonferroni-adjusted *p*-value, a maximum depth of the tree (*n* = 5), and a minimum size of end nodes (*n* = 20) as the stopping criteria. The application of a tree-based algorithm on the embryo data would help to more precisely define the effect of patient age and embryo quality (good-quality or poor-quality) on live birth outcome, and to better understand any interactions between these two clinical variables (patient age and embryo quality).

Note that while several other classification algorithms could have been employed for the prediction, CHAID enabled a user-friendly visualization of the resulting decision tree.^36,37^

As Fig. 6 shows, patients were automatically classified into three age groups: (i) ≤36, (ii) 37 and 38, and (iii) ≥39 years old due to age data distribution. For each age group, embryos were classified in good- and poor-quality groups (Supplementary Fig. 3c).

The results confirm the association between probability of successful pregnancy and patient age. The live birth probability for patients with good-quality embryos is significantly (1% Bonferroni-adjusted *p*-value) higher than that for patients with poor-quality embryos across different ages. Figure 6 indicates that patients ≤36 years old have a higher successful pregnancy rate compared to patients in the other two age groups. The CHAID decision tree analysis also indicates that the chance of favorable outcome using IVF varies from 13.8% (e.g., when the embryo is of poor-quality as assessed by STORK and the patient is ≥41 years old) to 66.3% (e.g., when the embryo is of good-quality and the patient is <37 years old) (Fig. 6).

### Probability analysis optimizes embryo selection and maximizes likelihood of single pregnancy

It is a common practice in IVF clinics to select and transfer more than one embryo in order to increase the chance of a successful pregnancy. As the success rate of individual embryos are typically <50% and transferring two or more embryos can increase the success probability. However, when the number of transferred embryos increases, the chance of multiple pregnancies (twins or even triplets) and associated complications also increase. For example, let’s simply assume we transfer three embryos each with an independent success probability of 1/4. The chance of a pregnancy can be, thus, calculated as *p* = 1−(3/4)^3^ ≈ 0.58. However, in this scenario, the chance of twin and triplets pregnancy would be 3 × (1/4)^2^(3/4) ≈ 0.14 and (1/4)^3^ ≈ 0.02, respectively. The chance of a single pregnancy would be 3 × (1/4)(3/4)^2^ ≈ 0.42.

Given a list of potential embryos for transfer and their predicted success rate using our decision tree analysis, we can calculate the probability of no pregnancy, single pregnancy, and multiple pregnancies, for any selection of embryos from the list. In general, if *k* embryos are transferred with indicated success probabilities of *p*_*1*_*, …, p*_*k*_ (where *p*_*i*_ shows the success probability of embryo *i*, for any index *i* between 1 and *k*) then the chance of a single pregnancy can be calculated as *P* *=* *∑*_*i*_ *p*_*i*_*∏*_*j≠i*_*(1−p*_*j*_*)*. Given the success rate of any individual embryo transfer, we showed how to calculate the probability of a single successful pregnancy when *k* embryos (*k* > 1) are transferred. This will help embryologists to select those embryos (for example two or three), which maximize the chance of a single pregnancy when transferred together.

## Discussion

Studies on human embryo evaluation are still very limited and mostly based on morphological features. They often involve low numbers of embryos from single centers, and they lack validations in independent cohorts. Furthermore, publications to date have relied on single static images. However, time-lapse images have the advantage of being consistent in terms of size, lighting, contrast, quality, and in terms of capturing the timing of embryo development, which is particularly important when quantifying blastocyst expansion. Currently, no robust and fully automatic method exists to analyze human embryo data by TLI.

Recently, there have been several studies utilizing classical machine learning approaches, such as support vector machine (SVM) and RF, and deep learning methods, such as CNN-basic,^15,16,38^ for outcome prediction or grade classification. To date, several AI methods have been used to assess blastocysts.^39^ Image segmentation and advanced image analysis techniques using neural networks with textured descriptors, level set, phase congruency, and fitting of ellipse methods have been demonstrated in mouse,^40^ bovine,^15^ and human blastocysts.^4,17^

More recently, Segal et al.^41^ have developed a random forest classifier-based tool using 2744 embryos to predict which patients should have extended culture with an accuracy of 76.4%. Also, Matsumoto et al.^42^ used time-lapse monitoring of 118 human embryos to determine good-quality embryos using deep learning-based method, which is based on the Keras neural network library. They achieved 70 and 80% accuracy for the validation dataset through two different cell stages that are significantly lower than the performance obtained from our framework. Besides, the advantage of our method is that instead of only focusing on the predetermined, segmented features that embryologists are trained to analyze, the entire image of the embryo is assessed, allowing for quantification of all the available data. Convolution, therefore, allows the AI to identify patterns in morphological features that we do not know how to assess.

We have demonstrated that deep learning approaches can provide accurate quality assessments in various clinical conditions. The STORK framework presented here provides a method that can be easily implemented for a wide range of applications, including embryo grading. Our method yields a cutting-edge sensitivity when performing the challenging task of assessing embryo quality using multi-focal embryo images. Notably, our STORK framework is fully automated and does not require any manual augmentations or preprocessing on the input images. In fact, it provides embryologists a straightforward platform to use without requiring sophisticated computational knowledge. Furthermore, we designed a decision tree based on the CHAID algorithm to investigate the interaction between embryo quality and patient characteristics (i.e., patient age) in a diverse population, and their effect on the likelihood of live birth.

Finally, although STORK can run on traditional computer microprocessors (CPUs), abundant system memory and graphics processing units (GPUs) make the training process faster (at least one order of magnitude) as training requires loading a significant number of medical images for training and validation.

Nevertheless, our method still has limitations. For example, we explored the possibility of directly predicting the likelihood of pregnancy based on only embryo images that are labeled as “positive live birth” or “negative live birth” (Supplementary Method 2). The result showed that the trained algorithm cannot identify positive live birth and negative live birth successfully using embryo morphology alone.

## Methods

### Images from human blastocysts

This study included 10,148 embryos from the Center for Reproductive Medicine at Weill Cornell Medicine (2012/05–2017/12). We referred to this dataset as WCM-NY throughout this manuscript. This study used retrospective and fully de-identified data. The study was performed in accordance with relevant guidelines and regulations and was approved by the Institutional Review Board at Weill Cornell Medicine (IRB number: 1401014735). The images were captured using the EmbryoScope® time-lapse system (Vitrolife, Sweden) with a built-in microscope. Images were captured using single red LED (635 nm) every 20 min., with seven focal depths (+45, +30, +15, 0, −15, −30, and −45) of the embryo taken each time, representing a total of 50,392 images (500 × 500 pixels) captured precisely 110 h post-insemination (hpi) (Fig. 2a). The standardization of images by the EmbryoScope software was consistent, and the images of the blastocysts were labeled using the Veeck and Zaninovic grading system^43^ (Supplementary Table 1). For validation, we also used two external datasets from the Universidad de Valencia, Valencia, Spain, and the Institute of Reproduction and Developmental Biology of Imperial College, London, UK (IRDB-IC) (Supplementary Table 2).

### Dataset preparation and the comprised images

This study presents a framework to classify different embryo images based on blastocyst grading and map those grades to good- and poor-quality.

In machine learning, especially for classification, a high-quality training dataset is important for training the classification model. Therefore, for the first step of selecting images, we manually eliminated the images with dark background and uninformative images (Supplementary Fig. 4) from the training set. Moreover, the number of images with good-quality usually is much less than that of poor-quality within the training sample. To avoid unbalanced classes for training the algorithm, we randomly deleted images from the class, which has sufficient observations (poor-quality) so that the comparative ratio of two classes is equal in our data.

For the next step, the embryologists labeled the embryo images with quality labels (good-quality, fair-quality, and poor-quality) to map certain quantitative scores from the grading system of Veeck and Zaninovic (e.g., 3AA, 3BB, 1BB) to three quality grades based on the pregnancy outcome obtained from statistical analysis of clinical data. In this regard, any grade that contained B- or C and an extension rate equal to or less than three was considered part of the poor-quality group with <35% pregnancy chance. In addition, any score with two A or A- grades, or one A with B, with an extension of 3 or greater could be labeled as good-quality with >58% pregnancy chance. However, the experts debated about some scores (e.g., 3BB, 3BA-), putting them in a separate category (fair-quality) as their pregnancy likelihood was about 50%. Then, fair-quality images were removed manually, and we were left with a total of 12,001 images for good- and poor-quality images to train the Inception-V1 algorithm using two quality groups at both ends of the spectrum. The complete list of scores and their quality map for good- and poor-quality are shown in Supplementary Table 5.

Finally, we divided the images into training, validation, and test groups. We allocated 70% of the images to the training group and the remaining 30% to the validation and test groups (Supplementary Table 3). The training, validation, and test sets (Supplementary Table 6) did not overlap.

### Algorithm architectures and training methods

We employed a deep neural network (DNN) for embryo image analysis based on Google’s Inception-V1^21^ architecture, which offers an effective run-time and computational cost.^44,45^

Convolutional neural networks (CNNs) comprise several convolutions to pass the result to the next layer, pooling layers to combine the outputs of neurons into a single neuron, and fully connected layers, which represents the outputs. Inception-V1 architecture (popularly known as GoogLeNet) utilizes nine inception modules made of 22 layers with parameters and 27, including pooling layers.^21^ The preliminary three convolution nodes are combined with two max pooling operations and followed by nine inception modules that are stacked linearly. The architecture ends with a fully connected layer and then a softmax output layer to map the non-normalized output to a probability distribution over predicted output classes.^21^

To train this architecture, we used transfer learning, which involves training the whole network. We initialized our network parameters to the best parameter set that was achieved on ImageNet database. We then fine-tuned the parameters of all of the convolution filters of all outer layers of the network on our data (WCM-NY images) via back propagation. The training process was run for 50,000 iterations and implemented using the TensorFlow software library.^46^

### Evaluation of method and implementation details

To implement our framework called STORK, we used Tensorflow version 1.4.0^46^ and the TF-Slim Python library for defining, training, and evaluating models in TensorFlow. Training of our DNN method was performed on a server running the SMP Linux operating system. This server is powered by four NVIDIA GeForce GTX 1080 GPUS with 8 GB of memory for each GPU and 12 1.7-GHz Intel Xeon CPUs.

To evaluate the performance of our method, we used an *accuracy* measure, which is the fraction of correctly identified images.^20^ To assess the performance of different algorithms, precision-recall curves (PRCs) were used. Additionally, receiver operating characteristics (ROCs) were estimated. The ROC curve is depicted by plotting the true positive rate (TPR) versus the false positive rate (FPR) at various threshold settings. The accuracy is measured by the area under the ROC curve (AUC).^47,48^

## Supplementary information

Supplemental merged

## Data Availability

The embryo imaging datasets analyzed in this study are not publicly available: due to reasonable privacy and security concerns, the embryo imaging data are not easily redistributable to researchers other than those engaged in the Institutional Review Board-approved research collaborations with the named medical centers. Our method is not specific to the datasets used in this study and users can train and test the deep learning model on any relevant imaging data.

## Supplementary information

**Supplementary Information** accompanies the paper on the *npj Digital Medicine* website (10.1038/s41746-019-0096-y).

## Code availability

The source code and the schematic guideline are publicly available at http://github.com/ih-lab/STORK. In addition, STORK is available through a web-based user interface at https://ivf-stork.org (password protected).
