# Supplementary material for: Deep learning enables robust assessment and selection of human blastocysts after in vitro fertilization
Source: NPJ Digit Med. 2019 Apr 4;2:21. doi: 10.1038/s41746-019-0096-y (PMC6550169; doi:10.1038/s41746-019-0096-y)
Supplement: Supplementary file 1 — Supplemental merged [file 41746_2019_96_MOESM1_ESM.pdf]

Electronic supplementary material

Deep Learning Enables Robust Assessment and Selection of Human Blastocysts after In Vitro Fertilization

Pegah Khosravi, Ehsan Kazemi, Qiansheng Zhan, Jonas E. Malmsten, Marco Toschi, Pantelis Zisimopoulos, Alexandros Sigaras, Stuart Lavery, Lee A.D. Cooper, Cristina Hickman, Marcos Meseguer, Zev Rosenwaks, Olivier Elemento, Nikica Zaninovic*, and Iman Hajirasouliha*

*Co-corresponding authors: imh2003@med.cornell.edu (IH), nizanin@med.cornell.edu (NZ)

Weill Cornell Medicine of Cornell University, New York, USA

Supplementary Methods

Supplementary Figures

Supplementary Tables

Supplementary Method 1. Embryologists split and merge the quantity grades

In this project, skilled embryologists determined the quantitative scores based on the grading system of Veeck and Zaninovic[43]. This grading system has three components: The first is a number showing the level of blastocyst expansion (CM, 1, 2, 3, 4, and 5), the second is a letter indicating the cell abundance and conformity in the ICM (grades A, B, C, and D), and the third is a letter quantifying the quality of TE cells (grades A, B, C, and D), which are extra-embryonic tissues that support the embryo proper (see).

For the first step of this project, the embryologists selected 13,931 images of embryos with good- and poor-quality based on their grades and pregnancy outcome. We converted various quantitative grades related to other data resources to the Veeck and Zaninovic[43] scoring system (**Supplementary Table 4**) before testing our trained algorithm with other clinical resources (**Supplementary Table 2**). For instance, the 3AA grade in our WCM-NY dataset is equivalent to the BEaa grade in the Universidad de Valencia dataset and the 4AA grade in the IRDB-IC dataset (**Supplementary Table 4**), which is based on the Gardner system[8,27]. Notably, these two datasets are less accurate compared to the WCM-NY dataset due to variations in the grading systems. Information about the grading systems used for the different datasets is shown in **Supplementary Table 4**.

Supplementary Method 2. Predicting pregnancy rate based on morphological quality of embryos

To explore the possibility of predicting the likelihood of pregnancy based on embryo images, we used WCM-NY images associated with 1,620 embryos for which we had the pregnancy outcome (live birth) information and the informative images (**Supplementary Table 3**). We allocated 85% of the embryos (1,377 embryos, 9,639 images) to build two classes-“negative live birth” (603 embryos) and “positive live birth” (774 embryos)-as training and validation. There were good- and poor-quality embryos that were assessed by embryologist, in both the “negative live birth” (embryos ‘a’ and ‘b’ in **Supplementary Figure 2**) and “positive live birth” classes (embryos ‘c’ and ‘d’ in **Supplementary Figure 2**). Thus, we had embryo images with four different characteristics in two classes (**Supplementary Figure 2**).

We built a new training algorithm, different from STORK, called DCNN (deep convolutional neural network) to fine-tune the Inception-V1 algorithm using two classes (positive and negative live birth) with 50,000 steps.

Finally, we tested DCNN with 243 randomly selected embryos as a blind test comprising 136 and 107 “positive live birth” and “negative live birth” embryos (1,701 images), respectively (**Supplementary Table 3**).

We obtained only a 51.85% accuracy for discretization of positive and negative live birth. This suggests that discretization of images based on live birth outcome using embryo morphology alone cannot be useful since other important characteristics,

such as the patient age and genetic or clinical variations, can affect the pregnancy rate.

We wondered what explained the low accuracy of DCNN in predicting pregnancy rate via positive and negative live birth. To find the reason, we looked closer at the results for embryos with four different characteristics (**Supplementary Figure 2**) that we integrated into two classes (positive and negative live birth).

We found that 28.85%, 47.27%, 41.02%, and 71.13% accuracy for a randomly selected test set (243 embryos) comprised “negative live birth” with “good-quality” (52 embryos) (embryo ‘a’ in **Supplementary Figure 2**), “negative live birth” with “poor-quality” (55 embryos) (embryo ‘b’ in **Supplementary Figure 2**), “positive live birth” with “poor-quality” (39 embryos) (embryo ‘c’ in **Supplementary Figure 2**), and “positive live birth” with “good quality” (97 embryos) (embryo ’d’ in **Supplementary Figure 2**), respectively.

This suggests that the trained algorithm can classify images based only on their quality (good or poor) while disregarding their outcome (positive or negative live birth) (**Supplementary Figure 2**). Therefore, the accuracy of DCNN could be increased if we utilized a larger number of images with “poor-quality and negative live birth” and “good-quality and positive live birth” in our test set. Moreover, the DCNN performance decreased due to the integration of good- and poor-quality images with, for example, “negative live birth” in a single class (e.g., embryos ’a’ and ’b’ in **Supplementary Figure 2**).

Supplementary Figures

a

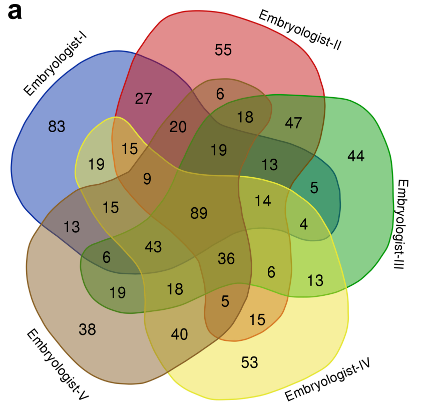

b

	Embryologists-I	Embryologists-II	Embryologists-III	Embryologists-IV	Embryologists-V
Embryologists-I		0.327	0.278	0.311	0.319
Embryologists-II	0.327		0.368	0.223	0.235
Embryologists-III	0.278	0.368		0.198	0.219
Embryologists-IV	0.311	0.223	0.198		0.374
Embryologists-V	0.319	0.235	0.219	0.374	

0 = agreement equivalent to chance
0.1 – 0.20 = slight agreement
0.21 – 0.40 = fair agreement
0.41 – 0.60 = moderate agreement
0.61 – 0.80 = substantial agreement
0.81 – 0.99 = near perfect agreement
1 = perfect agreement

Supplementary Figure 1. (a) This diagram[49] demonstrates the agreement among embryologists in the labeling of the same embryo images. The colors indicate different embryologists, and the numbers represent the number of embryos. (b) Cohen's Kappa percent that shows the low agreement among five embryologists for classification of 394 embryos in good-, fair-, and poor-quality classes. The Kappa statistic varies from 0 to 1.

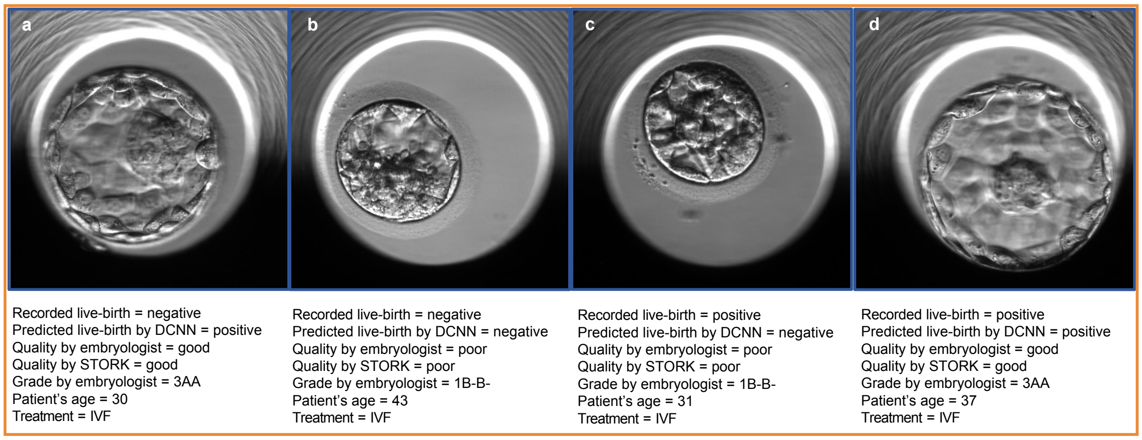

Supplementary Figure 2. The DCNN classifies embryo images with positive and negative live birth labels with a focus on their morphological quality. For example, embryos "a" and "d" are recorded by the laboratory data manager as negative live birth and positive live birth, respectively. DCNN, however, predicted positive live birth for embryos "a" and "d" because they both have good morphological quality. Embryos "b" and "c" are recorded as negative live birth and positive live birth, respectively. However, the algorithm again classified both embryos "b" and "c" as negative live birth because they have poor-quality.

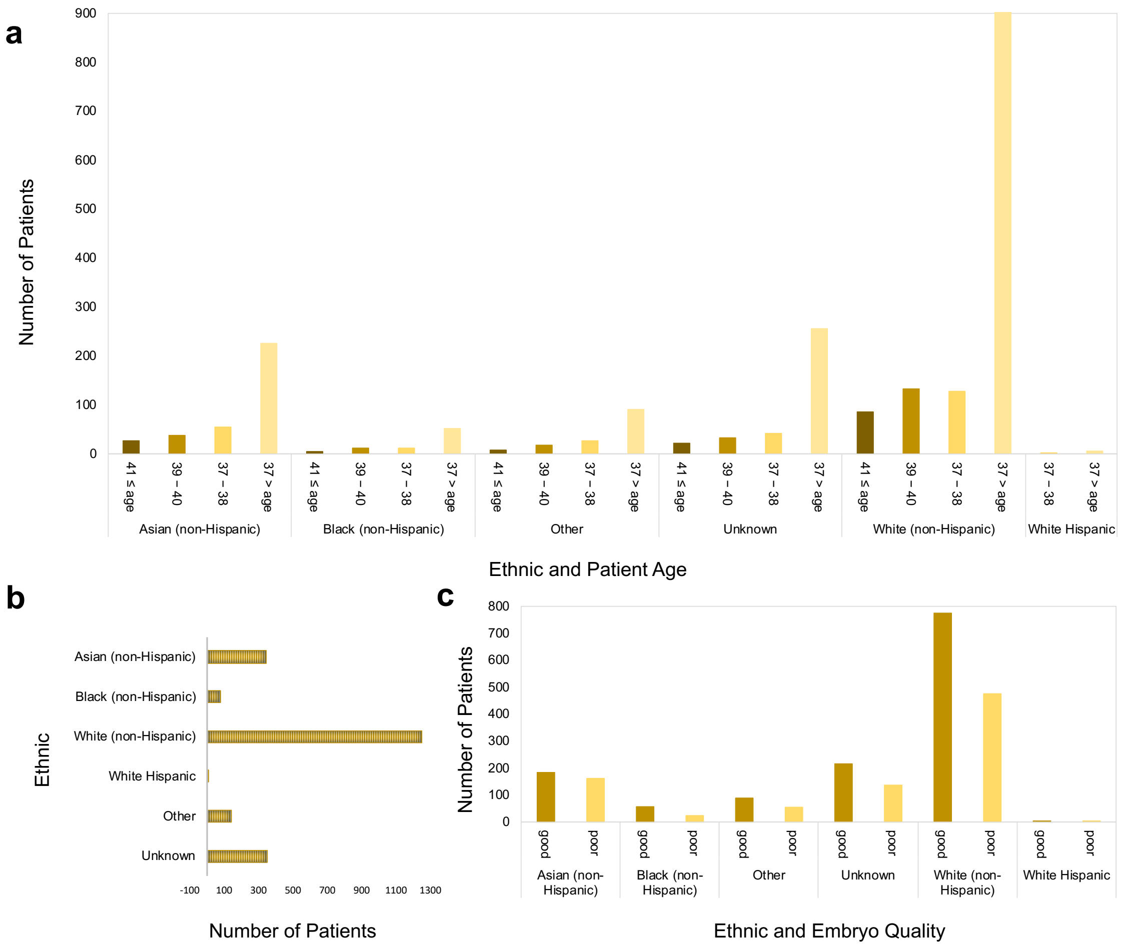

Supplementary Figure 3. (a) We used the fully de-identified data consists very diverse ethnic groups of patients through various age groups. (b) Although the ethnic majority groups in our study is white (non-Hispanic), the database consists other ethnic groups such as Asian and Black. (c) For each age group, embryos were classified in good- and poor-quality groups.

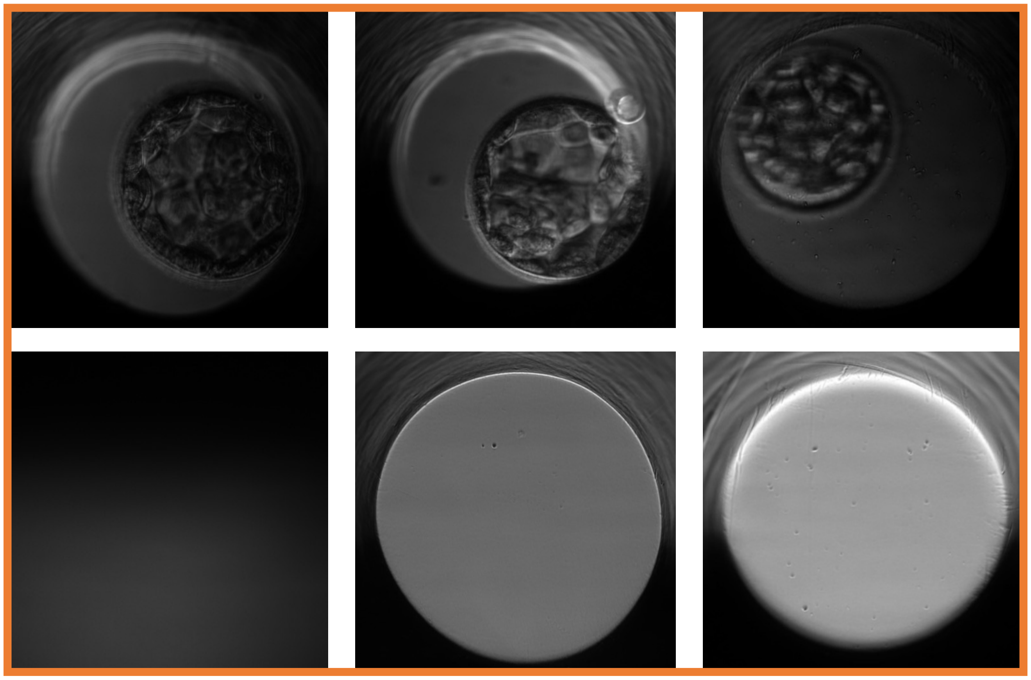

Supplementary Figure 4. Images with dark background and the uninformative images were eliminated from the training set.

Supplementary Tables

Supplementary Table 1. Characteristics of 130 various grades and their image numbers were labeled using the Veeck and Zaninovic grading system.

The morphological grades	Class size
1-2B-/CB, 1-2B-/CB-/C, 1-2B-C, 1-2BA-, 1B-/CB-/C, 1BC, 1CB-/C, 1CC, 2-2B-C, 2-3B-A-, 2-3BA, 2-3BC, 2AA, 2AB-/C, 3-4AA, 3A-B-/C, 3B-/CA, 3B-B-/B, 3BC, 3CA-, 3CB, 3CB-, 3CC, 4A-A, 4A-A-, 4AB-, 4B-/CB, 4B-/CB-, 5A-B, 5AA-, 5BA, 5BA-, 5BB-, 1-2A-B-, 1-2B-/CB-, 1B-/CB, 1B-/CB-, 1B-/CC, 2-3AA, 2-3AA-, 2-3B-/CB, 2-3B-/CB-, 2A-B-/C, 2B-/CB, 2BA, 3A-C, 3B-/CB-/C, 3B-/CC, 4B-B-, 4BB-, 5B-B, 1-2BB-/C, 1AB, 2B-/CB-/C, 3B-C, 4AB, 4B-B, 5A-A-, 5BB, 6BB, 1BB-/C, 1BB-/C, 1-2B-B-/C, 1A-B-, 1B-C, 2-3AB-, 2-3BB-/C, 4A-B, 4AA-, 4BA-, 2-3B-B-/C, 2A-A-, 2B-/CB-, 3B-A, 4AA, 2-3BA-, 1-2A-B, 1A-B, 2BA-, 3B-/CB-, 2AB, 5B-B-, 2AB-, MOR	Less than 10 images per grade
2-3A-B-, 3B-/CB, 2A-B-, 2BB-/C, 3B-A-, 4BB, 2-3AB, 2-3A-A-, CM, CAVM, 1B-B-/C, 2B-B-/C, 3BB-/C, 3BA, 3B-B-/C, 2A-B, 1-2B-B	More than 10 and less than 50 images per grade
2-3A-B, 1-2B-B-, 2-3B-B-, 3A-A, 1-2BB-, 3AB-, 2-3B-B	More than 50 and less than 100 images per grade
1B-B, 1BB-, 2-3BB-, 1-2BB, 1B-B-, 3A-B-, 3AA, 2B-B, 3BA-, 3AA-, 2B-B-, 2-3BB, 3AB, 2BB-, 3B-B-, 1BB, 3A-A-	More than 100 and less than 500 images per grade

2BB, 3B-B, 3BB-, 3A-B, 3BB	More than 500 images per grade

Supplementary Table 2. The results of applying STORK on various datasets to discriminate two classes of embryo quality. WCM-NY: The Center for Reproductive Medicine and Infertility at Weill Cornell Medicine of New York; Universidad de Valencia: Institute Valenciano de Infertilidad, Universidad de Valencia; IRDB-IC: Institute of Reproduction and Developmental Biology of Imperial College.

Datasets	Grades	Number of test embryos	STORK result (AUC)
WCM-NY	3AA, 3AA-, 3AB, 3A-A-, 5AA-, 3A-A, 3-4AA, 4AA (good-quality), 3BB-, 2BB-, 2BB, 1BB, 1B-B, 2B-B-, 1BB-, 1B-B-, 2B-B, 1-2BB, 3B-B, 1-2B-B-, 1-2BB-, 1BB-C, 1-2B-B, 3CB (poor-quality)	283	0.987
Universidad de Valencia	BEab, BEaa, BHiaa, BHiab, BHab (good-quality), BCbb, BCbc, BEcc, BEbc, BCcc, BEcb, BCcb (poor-quality)	127	0.759
IRDB-IC	4Aa, 4Ab, 5Ab, 5Aa (good-quality), 2Cb, 4Bc, 2Bc, 4Cc, 3Bc, 2Cc, 1Bb, 3Cc (poor-quality)	87	0.901

Supplementary Table 3. Four datasets showing different images (different number of embryos and clinical information) selected from the databases of WCM-NY (three datasets) and the Universidad de Valencia (one dataset) to assess the performance of STORK across different conditions.

Datasets	Dataset representation	Labels of inputs and outputs	Number of classes and images
Good-Poor	110hpi images of embryos (WCM-NY)	Discrimination of good- and poor-quality of embryos	2 classes: 12,001 images for training and 1,930 images for test set
Outcome-Quality	110hpi images of embryos (WCM-NY)	Discrimination of positive and negative outcome of embryos through good- and poor-quality of embryos	2 classes: 9,639 images for training and 1,701 images for test set
Five-Experts	110hpi images of embryos	Discrimination of good- and poor-	2 classes: 12,001 images for training

	(WCM-NY and Universidad de Valencia)	quality of embryos	(STORK as trained algorithm by WCM-NY dataset) and 394 embryos for test set from Universidad de Valencia database

Supplementary Table 4. Information about different grading systems in three clinics. The Veeck and Zaninovic grading system[43] is a slightly modified version of the Gardner system[27], classifying embryos based on blastocyst expansion (grades 1 to 6), cell abundance, and conformity in the ICM (grades A, B, and C) and TE (grades A, B, and C). We also used two other datasets from the Universidad de Valencia and the Institute of Reproduction and Developmental Biology of Imperial College (IRDB-IC). The data from the Universidad de Valencia was graded based on a slightly different of the Gardner scoring system known as Asebir[26]. Compared to the Gardner system, Asebir uses five rather than six expansion categories and changes the ICM and TE rating terminology to single A, B, C, and D letters. The IRDB-IC data was graded using the Gardner scoring system.

Objects	Veeck and Zaninovic	Gardner	Asebir
Expansion			
	CM	1	BT
	1	2	BT
	2	3	BC
	3	4	BE
	4	5	BHi
	5	6	BH
ICM			
	A	A	A
	B	A/B	B
	C	B/C	C
	D	C	D
TE			
	A	A	A
	B	A/B	B
	C	B/C	C
	D	C	D

Supplementary Table 5. The quantity scores that the algorithm is trained for. The embryologists categorized the scores into two groups (classes) and labeled them as good-quality and poor-quality.

The list of grades	The quality map
3-4AA, 4A-A, 4A-A-, 5AA-, 4AB, 5A-A-, 4AA-, 4AA, 3A-A, 3AA, 3AA-, 3AB, 3A-A-	good-quality
1-2B-/CB, 1-2B-/CB-/C, 1-2B-C, 1B-/CB-/C, 1BC, 1CB-/C, 1CC, 2-2B-C, 2-3BC, 3B-B-/B, 3BC, 3CA-, 3CB, 3CB-, 3CC, 1-2B-/CB-, 1B-/CB, 1B-/CB-, 1B-/CC, 2-3B-/CB, 2-3B-/CB-, 2B-/CB, 3B-/CB-/C, 3B-/CC, 1-2BB-/C, 2B-/CB-/C, 3B-C, 1BB-/C, 1BB-/C, 1-2B-B-/C, 1B-C, 2-3BB-/C, 2-3B-B-/C, 2B-/CB-, 3B-/CB-, 3B-/CB, 2BB-/C, 1B-B-/C, 2B-B-/C, 3BB-/C, 3B-B-/C, 1-2B-B, 1-2B-B-, 2-3B-B-, 1-2BB-, 2-3B-B, 1B-B, 1BB-, 2-3BB-, 1-2BB, 1B-B-, 2B-B, 2B-B-, 2BB-, 3B-B-, 1BB, 2BB, 3B-B, 3BB-	poor-quality

Supplementary Table 6. The following tables contain the STORK score for the test set (1930 images), patients clinical characteristics (their pregnancy outcome, patient age, and their embryo grade and quality) for 2,182 embryos, and the result of five embryologists for 394 embryos.

Images	good	poor	accuracy for images=97.5
good_14069738_-30_3AA	1.0000	0.0000	
good_19113441_-30_3A-A	1.0000	0.0000	
good_12024489_30_3AA-	1.0000	0.0000	
poor_78085332_15_2BB	0.0000	1.0000	
poor_78233182_30_1B-B-	0.0000	1.0000	
poor_79219063_15_3B-B	0.0862	0.9138	
poor_81322712_-45_1-2B-B	0.0000	1.0000	
good_16433634_-30_3A-A-	1.0000	0.0000	
good_12731906_-30_3A-A-	1.0000	0.0000	
poor_76994692_-15_2BB-	0.0006	0.9994	
poor_80058256_45_1BB	0.0000	1.0000	
good_13831587_45_3AB	1.0000	0.0000	
poor_77666018_-45_1B-B-	0.0000	1.0000	
poor_85900848_45_3B-B	0.0003	0.9997	
poor_78753524_0_1-2B-B-	0.0000	1.0000	
poor_78407847_-30_2BB-	0.0000	1.0000	
good_16621319_-15_3A-A-	1.0000	0.0000	
good_16286039_-15_3A-A-	0.8781	0.1219	
good_13318962_0_3AB	1.0000	0.0000	
good_16621319_45_3A-A-	1.0000	0.0000	
poor_77433878_15_1BB-	0.0000	1.0000	
good_12997501_15_3A-A-	1.0000	0.0000	
poor_88984960_-15_3CB	0.9974	0.0026	
good_10232189_30_3A-A-	1.0000	0.0000	
poor_81105911_30_1B-B	0.0000	1.0000	
poor_94062269_-45_3B-B	0.0000	1.0000	
poor_83626811_0_3B-B	0.0000	1.0000	
poor_83838129_0_3BB-	0.0000	1.0000	
good_17612946_-30_3A-A-	1.0000	0.0000	
poor_78928905_30_1BB	0.0000	1.0000	
poor_83199911_45_3BB-	0.0000	1.0000	
poor_83453674_-45_3BB-	0.0029	0.9971	
poor_78651678_15_1B-B-	0.0000	1.0000	
good_17164399_-45_3AB	1.0000	0.0000	
good_15764642_45_3A-A-	1.0000	0.0000	
good_19271216_15_3A-A-	0.0000	1.0000	
poor_83663432_15_1-2BB-	0.0001	0.9999	
good_16433634_45_3A-A-	1.0000	0.0000	
poor_80678895_-45_1B-B-	0.0000	1.0000	
good_15430377_15_3A-A-	1.0000	0.0000	
poor_82384146_15_1-2BB	0.0000	1.0000	
poor_83309479_45_1-2BB	0.0000	1.0000	
poor_92921469_30_3B-B	0.0000	1.0000	
poor_77059306_15_2BB	0.0000	1.0000	

good_11311361_0_3A-A-	1.0000	0.0000
good_11311361_-30_3A-A-	1.0000	0.0000
good_15892653_15_3A-A-	0.9703	0.0297
good_11969009_30_3AA	1.0000	0.0000
good_18213488_-15_3AB	1.0000	0.0000
good_10423835_15_3A-A-	1.0000	0.0000
poor_81526383_15_3BB-	0.0000	1.0000
good_13685820_-15_3A-A-	1.0000	0.0000
poor_83001010_-30_3BB-	0.1257	0.8743
good_10443637_15_3AA-	1.0000	0.0000
poor_77282204_-15_1B-B	0.0000	1.0000
poor_84565094_-30_3B-B	0.0000	1.0000
good_20498781_-15_3AB	1.0000	0.0000
good_14493269_0_3AA-	1.0000	0.0000
poor_79318932_-30_2BB-	0.0000	1.0000
good_17164399_30_3AB	1.0000	0.0000
poor_82384146_-45_1-2BB	0.0000	1.0000
good_17937817_-30_3A-A-	1.0000	0.0000
good_20496692_30_3A-A-	1.0000	0.0000
poor_80876604_30_1-2B-B	0.0000	1.0000
good_17990992_30_3A-A-	1.0000	0.0000
good_11486813_45_3AA-	1.0000	0.0000
poor_88330925_30_3BB-	0.0000	1.0000
poor_97314024_-15_3B-B	0.0000	1.0000
good_14411543_15_3AA-	0.9999	0.0001
good_14952697_0_3AA-	1.0000	0.0000
good_17591250_0_3AA	1.0000	0.0000
poor_98690041_0_3B-B	0.0000	1.0000
poor_79535552_15_1BB-C	0.0000	1.0000
good_16880651_-30_3AB	1.0000	0.0000
poor_84080274_-30_1-2B-B	0.0000	1.0000
good_11142866_-15_3A-A-	1.0000	0.0000
good_11838370_15_3AB	0.9971	0.0029
good_11084942_-30_3AB	1.0000	0.0000
poor_82127295_-30_1BB-	0.0000	1.0000
poor_78715791_-30_3BB-	0.0000	1.0000
poor_77206651_0_2B-B-	0.0001	0.9999
poor_77004779_-45_2BB	0.0000	1.0000
good_12024489_-45_3AA-	0.9994	0.0006
poor_78651678_-30_1B-B-	0.0000	1.0000
poor_77004779_30_2BB	0.0000	1.0000
good_16130067_-45_3A-A-	1.0000	0.0000
poor_80296580_45_3BB-	0.0001	0.9999
good_12097042_-30_3AB	1.0000	0.0000
good_20498781_45_3AB	0.9992	0.0008

good_18570412_0_3AB	1.0000	0.0000
poor_80310340_30_1B-B-	0.0000	1.0000
poor_84080274_45_1-2B-B	0.0000	1.0000
poor_78970539_0_2BB	0.0001	0.9999
good_14493269_45_3AA-	1.0000	0.0000
poor_79424795_-15_2B-B-	0.0000	1.0000
good_10046346_-30_3AA	1.0000	0.0000
poor_77632697_0_2BB-	0.0000	1.0000
good_13158796_0_3A-A	0.9996	0.0004
poor_83663432_-30_1-2BB-	0.0000	1.0000
good_11957580_-45_3AA-	1.0000	0.0000
good_17612946_30_3A-A-	0.9983	0.0017
poor_81033173_30_1-2BB	0.0001	0.9999
good_19141554_-45_3AA-	1.0000	0.0000
good_11132559_45_3A-A-	1.0000	0.0000
poor_88984960_30_3CB	0.9971	0.0029
good_18102857_30_3AA-	1.0000	0.0000
poor_79085047_15_1BB	0.0000	1.0000
poor_83582148_-30_1B-B-	0.0000	1.0000
poor_79231202_30_3BB-	0.1104	0.8896
poor_79751379_-15_2BB	0.0000	1.0000
poor_84116513_-15_1BB-	0.0000	1.0000
good_20253695_30_3AB	1.0000	0.0000
poor_77725120_45_2B-B	0.0000	1.0000
poor_83990350_-30_1B-B-	0.0003	0.9997
poor_78869483_-15_3B-B	0.0002	0.9998
poor_83666558_30_1BB-	0.0000	1.0000
poor_79431255_45_1BB	0.0777	0.9223
poor_77898499_-45_1BB	0.0034	0.9966
poor_79365441_-45_2BB-	0.0000	1.0000
good_15075835_-15_3A-A-	1.0000	0.0000
good_15697722_45_3AA-	1.0000	0.0000
good_17937817_0_3A-A-	1.0000	0.0000
poor_77519659_-30_1BB-	0.0002	0.9998
good_17340290_45_3A-A-	1.0000	0.0000
good_13117449_-45_3A-A-	1.0000	0.0000
good_16837019_-15_3A-A-	1.0000	0.0000
good_13991233_45_3AA-	1.0000	0.0000
poor_79496470_30_1BB	0.0001	0.9999
poor_81526383_-45_3BB-	0.0000	1.0000
good_10423835_-45_3A-A-	1.0000	0.0000
poor_94994695_-45_3B-B	0.0010	0.9990
good_17392842_0_3A-A-	1.0000	0.0000
good_17248977_15_3A-A-	1.0000	0.0000
poor_81322712_45_1-2B-B	0.0003	0.9997

poor_83001010_15_3BB-	0.0048	0.9952
good_20253695_-15_3AB	1.0000	0.0000
good_13831587_0_3AB	0.9758	0.0242
poor_80296580_0_3BB-	0.0000	1.0000
poor_98690041_15_3B-B	0.0000	1.0000
good_17887898_0_3A-A-	0.0000	1.0000
poor_82745365_15_3BB-	0.0000	1.0000
poor_85900848_-15_3B-B	0.0000	1.0000
poor_77867306_-15_1BB	0.0000	1.0000
poor_77509010_15_2BB-	0.0000	1.0000
good_17709370_0_3A-A	0.9999	0.0001
good_16004053_15_3A-A-	1.0000	0.0000
poor_79535552_-45_1BB-C	0.0000	1.0000
good_20123884_30_3AA-	1.0000	0.0000
good_14005274_-30_3AB	1.0000	0.0000
good_13533711_-15_3AA	1.0000	0.0000
poor_78407847_30_2BB-	0.0000	1.0000
poor_77175651_15_2BB	0.0027	0.9973
poor_81581513_-45_3BB-	0.0421	0.9579
poor_77059306_-15_2BB	0.0001	0.9999
good_14331589_-15_3A-A-	1.0000	0.0000
good_19247486_-30_3AA-	1.0000	0.0000
good_17937817_30_3A-A-	1.0000	0.0000
poor_77272153_-45_2BB-	0.0025	0.9975
good_19247486_0_3AA-	0.9988	0.0012
good_11922626_-15_3A-A-	1.0000	0.0000
poor_79675372_30_3BB-	1.0000	0.0000
good_11486813_-15_3AA-	1.0000	0.0000
good_13729954_15_4AA	1.0000	0.0000
good_20123884_-15_3AA-	1.0000	0.0000
good_14331589_0_3A-A-	1.0000	0.0000
good_14952697_-45_3AA-	1.0000	0.0000
good_14851789_-45_3AA	1.0000	0.0000
poor_79431255_0_1BB	0.0000	1.0000
poor_94202441_45_3B-B	0.0000	1.0000
good_14853831_-30_3A-A-	1.0000	0.0000
poor_88330925_0_3BB-	0.0000	1.0000
poor_78164186_15_3BB-	0.0006	0.9994
poor_83990350_45_1B-B-	0.0043	0.9957
good_13852248_-45_3AB	1.0000	0.0000
good_10718644_-30_3AA	1.0000	0.0000
poor_78753524_30_1-2B-B-	0.0000	1.0000
good_11142866_15_3A-A-	1.0000	0.0000
good_20496692_-45_3A-A-	1.0000	0.0000
good_11838370_0_3AB	0.9953	0.0047

poor_79365441_30_2BB-	0.0000	1.0000
good_19998381_-15_3A-A-	0.8543	0.1457
poor_83199911_0_3BB-	0.0001	0.9999
poor_78970539_30_2BB	0.0000	1.0000
poor_79545498_-30_1-2B-B	0.0002	0.9998
good_11743470_0_3A-A-	1.0000	0.0000
poor_79441878_-45_1B-B-	0.0000	1.0000
poor_78341980_15_3B-B	0.0000	1.0000
poor_93031274_45_3B-B	0.0000	1.0000
poor_79968167_45_1BB	0.0000	1.0000
poor_80384973_45_3BB-	0.0001	0.9999
poor_78085332_-30_2BB	0.0000	1.0000
poor_79496470_-15_1BB	0.0545	0.9455
good_17887898_30_3A-A-	0.0000	1.0000
good_20236076_15_3A-A-	0.0011	0.9989
poor_78265703_15_2BB-	0.0000	1.0000
poor_80384973_-15_3BB-	0.0018	0.9982
poor_96225317_45_3B-B	0.0011	0.9989
good_15764642_-30_3A-A-	1.0000	0.0000
poor_79146459_15_2B-B-	0.0000	1.0000
poor_84565094_45_3B-B	0.0000	1.0000
poor_77681626_-45_1B-B	0.0000	1.0000
poor_78233182_-30_1B-B-	0.0000	1.0000
good_20236076_-30_3A-A-	0.0000	1.0000
poor_79751379_30_2BB	0.0000	1.0000
poor_92602106_-30_3B-B	0.0000	1.0000
good_17585937_15_3AA-	1.0000	0.0000
poor_84092585_-15_1BB-	0.0000	1.0000
good_19070722_-15_3AA-	1.0000	0.0000
poor_78928905_-30_1BB	0.0000	1.0000
poor_82153169_-30_3B-B	0.0000	1.0000
poor_78176117_-45_1-2BB	0.0000	1.0000
good_16880651_0_3AB	1.0000	0.0000
poor_78848868_-30_2BB	0.0002	0.9998
poor_77725120_0_2B-B	0.0000	1.0000
good_15075835_0_3A-A-	1.0000	0.0000
good_13858706_-45_3AA-	1.0000	0.0000
good_20185605_30_3AA	1.0000	0.0000
good_20498781_0_3AB	1.0000	0.0000
poor_82168233_30_1-2B-B	0.0000	1.0000
poor_78869483_45_3B-B	0.0019	0.9981
good_18803285_15_3AB	1.0000	0.0000
poor_84092585_15_1BB-	0.0000	1.0000
good_19678783_-15_3AA-	1.0000	0.0000
good_19030331_-30_3AA	1.0000	0.0000

poor_77867306_30_1BB	0.0000	1.0000
poor_78828717_-15_2B-B	0.0008	0.9992
poor_88330925_-15_3BB-	0.0002	0.9998
poor_78304592_-45_2B-B-	0.0000	1.0000
poor_78548643_15_1BB	0.0000	1.0000
poor_79231202_-30_3BB-	0.5441	0.4559
poor_78334440_-45_2BB	0.0000	1.0000
good_17257791_-30_3AA	1.0000	0.0000
poor_79431035_45_1B-B	0.0000	1.0000
poor_79380966_30_3B-B	0.0026	0.9974
good_14851789_15_3AA	1.0000	0.0000
poor_79161507_30_1-2BB	0.0000	1.0000
poor_76986756_-30_3BB-	0.0000	1.0000
good_12997501_-45_3A-A-	1.0000	0.0000
good_16390514_30_3AA	1.0000	0.0000
good_17641234_-45_3A-A-	1.0000	0.0000
good_14164726_-30_3A-A-	1.0000	0.0000
good_11139600_-30_3AA	1.0000	0.0000
good_18570412_-15_3AB	1.0000	0.0000
poor_79867694_-15_1BB	0.0000	1.0000
poor_77390363_15_1BB-	0.0000	1.0000
poor_88503434_45_3BB-	0.0000	1.0000
poor_78284204_-15_2B-B	0.0000	1.0000
good_16130067_30_3A-A-	1.0000	0.0000
poor_79380966_0_3B-B	0.0014	0.9986
good_14994218_-30_3A-A-	1.0000	0.0000
good_14331589_-45_3A-A-	1.0000	0.0000
good_11922626_-45_3A-A-	1.0000	0.0000
poor_79545498_0_1-2B-B	0.0000	1.0000
poor_76986756_0_3BB-	0.0000	1.0000
good_17454864_-15_3AA-	1.0000	0.0000
good_11302954_-15_3AA-	0.0000	1.0000
good_13158796_-30_3A-A	0.9793	0.0207
good_19890072_0_3AB	0.9863	0.0137
poor_79441878_-15_1B-B-	0.0000	1.0000
good_17585937_-45_3AA-	0.9998	0.0002
good_19998381_-45_3A-A-	0.1735	0.8265
good_17612946_0_3A-A-	0.8612	0.1388
poor_78555781_30_2BB	0.0000	1.0000
good_15697722_0_3AA-	1.0000	0.0000
good_20496692_-15_3A-A-	1.0000	0.0000
poor_96225317_-45_3B-B	0.0083	0.9917
good_11142866_45_3A-A-	1.0000	0.0000
poor_78753524_45_1-2B-B-	0.0000	1.0000
poor_91094982_30_3B-B	0.0002	0.9998

poor_83990350_15_1B-B-	0.0000	1.0000
good_12741796_30_3AB	1.0000	0.0000
good_13858706_15_3AA-	1.0000	0.0000
good_19449529_0_3AA-	1.0000	0.0000
good_10423835_-15_3A-A-	0.9991	0.0009
good_18781105_0_3AA	1.0000	0.0000
good_18102857_0_3AA-	1.0000	0.0000
good_17248977_45_3A-A-	0.9998	0.0002
good_13729954_-30_4AA	1.0000	0.0000
good_16837019_-45_3A-A-	1.0000	0.0000
poor_83838129_30_3BB-	0.0000	1.0000
good_17340290_15_3A-A-	1.0000	0.0000
good_13117449_-15_3A-A-	1.0000	0.0000
good_16621319_0_3A-A-	1.0000	0.0000
poor_77772754_15_1BB-	0.0000	1.0000
good_10443637_-45_3AA-	1.0000	0.0000
poor_78814052_-45_2BB	0.0000	1.0000
poor_79318932_15_2BB-	0.0000	1.0000
good_15075835_-45_3A-A-	1.0000	0.0000
good_10046346_0_3AA	1.0000	0.0000
good_16416586_-30_3AB	1.0000	0.0000
poor_78869483_0_3B-B	0.0019	0.9981
poor_77788406_15_2B-B	0.0000	1.0000
good_16004053_45_3A-A-	1.0000	0.0000
poor_78828717_45_2B-B	0.0000	1.0000
poor_81322712_15_1-2B-B	0.0002	0.9998
poor_79535552_-15_1BB-C	0.0000	1.0000
poor_77196120_15_1B-B	0.0000	1.0000
good_17257791_30_3AA	1.0000	0.0000
poor_80428295_45_3BB-	0.1334	0.8666
good_19536372_15_3AB	0.6890	0.3110
good_14164726_30_3A-A-	1.0000	0.0000
good_18213488_45_3AB	0.9995	0.0005
good_17516331_15_3AA-	1.0000	0.0000
good_13318962_-15_3AB	1.0000	0.0000
good_10101873_-45_3AA-	1.0000	0.0000
good_19271216_-15_3A-A-	0.0000	1.0000
good_13158796_15_3A-A	0.9675	0.0325
poor_83199911_-45_3BB-	0.0005	0.9995
good_14486014_-45_3AB	1.0000	0.0000
good_15764642_0_3A-A-	1.0000	0.0000
poor_78164186_0_3BB-	0.0000	1.0000
poor_87749651_15_3B-B	0.0000	1.0000
poor_83666558_-30_1BB-	0.0000	1.0000
good_16416586_45_3AB	1.0000	0.0000

poor_78634338_-15_2BB	0.0000	1.0000
good_17641234_-15_3A-A-	1.0000	0.0000
poor_78304592_30_2B-B-	0.0005	0.9995
good_18240544_-30_3A-A-	0.9362	0.0638
good_19449529_30_3AA-	1.0000	0.0000
good_12997501_-15_3A-A-	1.0000	0.0000
poor_81678844_-30_3BB-	0.0000	1.0000
poor_83453674_0_3BB-	0.1220	0.8780
poor_84565094_0_3B-B	0.0000	1.0000
poor_78210494_-15_1BB-	0.0000	1.0000
poor_78304592_-15_2B-B-	0.0000	1.0000
poor_79146459_45_2B-B-	0.0000	1.0000
good_17392842_30_3A-A-	1.0000	0.0000
good_17591250_-15_3AA	1.0000	0.0000
poor_77681626_45_1B-B	0.0001	0.9999
poor_82313942_15_1-2B-B-	0.0000	1.0000
good_17454864_45_3AA-	1.0000	0.0000
good_20236076_45_3A-A-	0.9408	0.0592
poor_83582148_0_1B-B-	0.0000	1.0000
poor_77320590_-45_3BB-	0.0000	1.0000
good_11838370_-15_3AB	0.5040	0.4960
poor_79861101_15_2BB-	0.0000	1.0000
poor_79545873_0_2BB-	0.0000	1.0000
poor_79478757_-15_2BB	0.0000	1.0000
good_15453155_15_3AB	1.0000	0.0000
poor_78233182_0_1B-B-	0.0000	1.0000
poor_78176117_-15_1-2BB	0.0000	1.0000
good_10232189_0_3A-A-	1.0000	0.0000
good_16482386_45_3AA	1.0000	0.0000
good_19998381_0_3A-A-	0.9963	0.0037
good_12828151_30_3AB	1.0000	0.0000
good_18707760_15_3AA	1.0000	0.0000
good_13261081_30_3AB	0.9999	0.0001
good_16416586_0_3AB	1.0000	0.0000
poor_81105911_-45_1B-B	0.0000	1.0000
poor_83626811_-30_3B-B	0.0000	1.0000
good_19238542_0_3A-A-	1.0000	0.0000
good_17710633_45_3AB	1.0000	0.0000
poor_77519659_0_1BB-	0.2298	0.7702
poor_78814052_30_2BB	0.0000	1.0000
poor_82384146_45_1-2BB	0.0027	0.9973
poor_83309479_15_1-2BB	0.0000	1.0000
poor_94202441_-30_3B-B	0.1354	0.8646
good_15430377_45_3A-A-	1.0000	0.0000
good_17300193_30_3A-A-	1.0000	0.0000

good_18935832_0_3A-A-	1.0000	0.0000
poor_80678895_-15_1B-B-	0.0000	1.0000
poor_80051850_0_2BB	0.0000	1.0000
poor_78164186_-30_3BB-	0.0000	1.0000
good_16433634_15_3A-A-	1.0000	0.0000
good_19334426_-30_3A-A-	1.0000	0.0000
good_10718644_15_3AA	1.0000	0.0000
poor_78651678_45_1B-B-	0.0000	1.0000
good_15764642_15_3A-A-	1.0000	0.0000
good_12097042_45_3AB	1.0000	0.0000
good_13831587_-45_3AB	1.0000	0.0000
poor_76994692_30_2BB-	0.0000	1.0000
good_13318962_45_3AB	1.0000	0.0000
poor_78085332_0_2BB	0.0000	1.0000
poor_79478757_15_2BB	0.0000	1.0000
poor_84116513_45_1BB-	0.0000	1.0000
poor_81678844_15_3BB-	0.0000	1.0000
good_17710633_-45_3AB	1.0000	0.0000
poor_77175651_-15_2BB	0.0000	1.0000
good_13991233_-45_3AA-	1.0000	0.0000
good_10163149_-15_3AB	1.0000	0.0000
good_13685820_-45_3A-A-	1.0000	0.0000
good_10423835_45_3A-A-	0.9728	0.0272
good_13533711_45_3AA	1.0000	0.0000
good_18102857_-15_3AA-	1.0000	0.0000
poor_79620727_15_1BB-	0.0000	1.0000
good_10423352_-15_3AA-	1.0000	0.0000
poor_83663432_45_1-2BB-	0.0000	1.0000
poor_80042420_30_3BB-	0.0049	0.9951
poor_93633156_15_3B-B	0.0000	1.0000
poor_79431255_-45_1BB	0.0000	1.0000
poor_77666018_-15_1B-B-	0.0000	1.0000
good_18935832_30_3A-A-	1.0000	0.0000
poor_79968167_0_1BB	0.0000	1.0000
good_11183795_-45_3AB	1.0000	0.0000
poor_82127295_0_1BB-	0.0000	1.0000
good_16286039_0_3A-A-	0.9928	0.0072
good_20337692_30_3AB	1.0000	0.0000
good_12731906_0_3A-A-	1.0000	0.0000
good_15481025_45_3A-A	1.0000	0.0000
good_11124729_-30_3AA	1.0000	0.0000
poor_81322712_-15_1-2B-B	0.0000	1.0000
poor_78176117_30_1-2BB	0.0000	1.0000
poor_77320590_0_3BB-	0.0000	1.0000
poor_77519659_45_1BB-	0.0000	1.0000

poor_78810664_0_1B-B-	0.0000	1.0000
poor_79620727_-15_1BB-	0.0000	1.0000
poor_77666018_0_1B-B-	0.0000	1.0000
good_16621319_15_3A-A-	1.0000	0.0000
good_16621319_-45_3A-A-	1.0000	0.0000
poor_76986756_45_3BB-	0.0000	1.0000
good_16286039_-45_3A-A-	0.9913	0.0087
poor_93031274_-15_3B-B	0.0001	0.9999
good_12997501_45_3A-A-	1.0000	0.0000
good_16433634_0_3A-A-	1.0000	0.0000
good_18803285_0_3AB	1.0000	0.0000
good_20337692_0_3AB	1.0000	0.0000
poor_87749651_-30_3B-B	0.0000	1.0000
good_16390514_-45_3AA	1.0000	0.0000
poor_82745365_-45_3BB-	0.0000	1.0000
good_14411543_-30_3AA-	1.0000	0.0000
good_11132559_15_3A-A-	1.0000	0.0000
good_11743470_30_3A-A-	1.0000	0.0000
good_18803285_-30_3AB	1.0000	0.0000
poor_82127295_45_1BB-	0.0000	1.0000
poor_79424795_-45_2B-B-	0.0000	1.0000
poor_78909238_30_3BB-	0.0000	1.0000
poor_77308012_-30_1BB-	0.0000	1.0000
poor_92602106_30_3B-B	0.0000	1.0000
good_17003193_-30_3AA	1.0000	0.0000
good_16837019_0_3A-A-	1.0000	0.0000
poor_80876604_0_1-2B-B	0.0000	1.0000
poor_79675372_-45_3BB-	0.9993	0.0007
poor_84080274_15_1-2B-B	0.0000	1.0000
poor_80051850_30_2BB	0.0000	1.0000
good_10163149_45_3AB	1.0000	0.0000
poor_78848868_15_2BB	0.0000	1.0000
poor_82153169_45_3B-B	0.0000	1.0000
good_15453155_0_3AB	1.0000	0.0000
poor_99187238_30_3B-B	0.0000	1.0000
good_15697722_-45_3AA-	0.9999	0.0001
good_11957580_15_3AA-	1.0000	0.0000
poor_79535552_45_1BB-C	0.0000	1.0000
poor_78548643_-45_1BB	0.0000	1.0000
good_19890072_-45_3AB	0.9924	0.0076
poor_79463940_45_2BB	0.0000	1.0000
good_14046542_30_3A-A-	1.0000	0.0000
poor_77772754_-30_1BB-	0.0000	1.0000
poor_82384146_-15_1-2BB	0.0000	1.0000
poor_92921469_-45_3B-B	0.0693	0.9307

poor_77272153_45_2BB-	0.0021	0.9979
good_20106212_-30_3AA-	1.0000	0.0000
poor_79085047_-15_1BB	0.0000	1.0000
good_19070722_45_3AA-	1.0000	0.0000
good_19247486_15_3AA-	0.9966	0.0034
poor_77509010_-15_2BB-	0.0000	1.0000
good_14005274_45_3AB	1.0000	0.0000
poor_77189946_-30_1BB	0.0000	1.0000
good_14486014_45_3AB	0.9998	0.0002
good_10232189_-30_3A-A-	1.0000	0.0000
good_17321648_45_3AB	1.0000	0.0000
good_18707760_-30_3AA	1.0000	0.0000
good_14493269_-45_3AA-	1.0000	0.0000
good_19113441_45_3A-A	1.0000	0.0000
poor_80058256_-45_1BB	0.0000	1.0000
good_16130067_-15_3A-A-	1.0000	0.0000
poor_88893942_15_3BB-	0.7928	0.2072
good_20106212_30_3AA-	1.0000	0.0000
good_19030331_30_3AA	1.0000	0.0000
poor_83453674_45_3BB-	0.9648	0.0352
good_11142866_-45_3A-A-	1.0000	0.0000
poor_77206651_30_2B-B-	0.0042	0.9958
good_14994218_30_3A-A-	1.0000	0.0000
poor_92602106_-15_3B-B	0.0000	1.0000
good_20498781_-30_3AB	1.0000	0.0000
good_19527698_45_3A-A	1.0000	0.0000
poor_94202441_0_3B-B	0.0265	0.9735
good_17164399_15_3AB	1.0000	0.0000
good_10423352_45_3AA-	1.0000	0.0000
poor_84092585_-30_1BB-	0.0000	1.0000
good_19070722_-30_3AA-	1.0000	0.0000
good_17585937_30_3AA-	1.0000	0.0000
poor_81406987_30_1-2BB-	0.0000	1.0000
good_16130067_0_3A-A-	1.0000	0.0000
poor_83309479_-45_1-2BB	0.0000	1.0000
poor_81033173_-30_1-2BB	0.0008	0.9992
good_14661384_30_3A-A-	1.0000	0.0000
poor_94994695_45_3B-B	0.0000	1.0000
poor_78909238_-45_3BB-	0.0000	1.0000
poor_82153169_-15_3B-B	0.0000	1.0000
good_10239739_45_3AA	1.0000	0.0000
good_16880651_-15_3AB	1.0000	0.0000
good_11969009_0_3AA	1.0000	0.0000
poor_78810664_15_1B-B-	0.0000	1.0000
good_11838370_30_3AB	0.9998	0.0002

good_11084942_-15_3AB	1.0000	0.0000
good_10101873_45_3AA-	1.0000	0.0000
poor_84106579_0_1B-B-	0.0000	1.0000
poor_78265703_30_2BB-	0.0000	1.0000
poor_84116513_0_1BB-	0.0000	1.0000
poor_78810664_-45_1B-B-	0.0000	1.0000
good_11486813_0_3AA-	1.0000	0.0000
good_13685820_45_3A-A-	1.0000	0.0000
poor_78828717_0_2B-B	0.0002	0.9998
poor_83838129_-45_3BB-	0.0000	1.0000
poor_82168233_0_1-2B-B	0.0000	1.0000
poor_77004779_15_2BB	0.0000	1.0000
good_15481025_-45_3A-A	1.0000	0.0000
good_12097042_-15_3AB	0.9997	0.0003
poor_80384973_-30_3BB-	0.0063	0.9937
good_19890072_45_3AB	1.0000	0.0000
poor_78265703_0_2BB-	0.0000	1.0000
good_10046346_-15_3AA	1.0000	0.0000
good_18432821_-45_3AB	1.0000	0.0000
poor_88330925_-30_3BB-	0.0000	1.0000
poor_78828717_-30_2B-B	0.0000	1.0000
poor_80530664_15_1-2B-B	0.0000	1.0000
good_15481025_0_3A-A	1.0000	0.0000
poor_79231202_-15_3BB-	0.0704	0.9296
good_14952697_45_3AA-	0.9989	0.0011
poor_79161507_-15_1-2BB	0.0000	1.0000
poor_79380966_15_3B-B	0.0007	0.9993
poor_76986756_-15_3BB-	0.0003	0.9997
poor_88984960_15_3CB	0.9983	0.0017
poor_79085047_30_1BB	0.0000	1.0000
poor_84106579_-30_1B-B-	0.0000	1.0000
good_11142866_0_3A-A-	1.0000	0.0000
poor_79751379_-30_2BB	0.0000	1.0000
good_17750221_-45_3A-A-	1.0000	0.0000
poor_83582148_15_1B-B-	0.0000	1.0000
good_20255380_30_3A-A-	1.0000	0.0000
good_20253695_15_3AB	1.0000	0.0000
poor_84092585_30_1BB-	0.0000	1.0000
good_19678783_-30_3AA-	1.0000	0.0000
good_20255380_-45_3A-A-	1.0000	0.0000
good_19238542_15_3A-A-	0.9976	0.0024
good_18657357_-45_3AA-	0.2248	0.7752
good_17321648_-45_3AB	1.0000	0.0000
good_14069738_-15_3AA	1.0000	0.0000
poor_77852541_45_2BB-	0.0000	1.0000

poor_78085332_30_2BB	0.0000	1.0000
poor_81406987_-45_1-2BB-	0.0000	1.0000
good_15472679_45_3AA-	1.0000	0.0000
poor_85900848_-30_3B-B	0.0000	1.0000
good_17641234_30_3A-A-	1.0000	0.0000
poor_77898499_45_1BB	0.0000	1.0000
poor_98690041_30_3B-B	0.0000	1.0000
poor_82745365_30_3BB-	0.0000	1.0000
poor_77509010_30_2BB-	0.0000	1.0000
good_17750221_30_3A-A-	1.0000	0.0000
good_11922626_15_3A-A-	1.0000	0.0000
good_12097042_0_3AB	1.0000	0.0000
poor_83309479_0_1-2BB	0.0000	1.0000
poor_83666558_15_1BB-	0.0000	1.0000
poor_83626811_45_3B-B	0.0000	1.0000
poor_79311557_45_1-2BB-	0.4505	0.5495
good_11666570_-45_3-4AA	1.0000	0.0000
poor_78869483_-30_3B-B	0.0026	0.9974
poor_94062269_45_3B-B	0.0000	1.0000
good_11666570_15_3-4AA	1.0000	0.0000
poor_77519659_-15_1BB-	0.2485	0.7515
poor_88984960_-30_3CB	0.9994	0.0006
poor_80530664_0_1-2B-B	0.0000	1.0000
good_11302954_45_3AA-	0.0000	1.0000
poor_77655020_-45_2BB-	0.0002	0.9998
poor_77852541_-45_2BB-	0.0000	1.0000
poor_83001010_30_3BB-	0.0013	0.9987
poor_78928905_15_1BB	0.0000	1.0000
poor_98690041_-45_3B-B	0.0000	1.0000
good_18240544_15_3A-A-	0.9999	0.0001
good_20106212_0_3AA-	1.0000	0.0000
poor_78164186_30_3BB-	0.0113	0.9887
good_15430377_0_3A-A-	1.0000	0.0000
good_19527698_-45_3A-A	1.0000	0.0000
poor_77320590_45_3BB-	0.0000	1.0000
good_13198789_-45_3AA	1.0000	0.0000
poor_79365441_15_2BB-	0.0000	1.0000
poor_77655020_45_2BB-	0.0000	1.0000
poor_78341980_30_3B-B	0.0000	1.0000
poor_77059306_30_2BB	0.0002	0.9998
poor_79219063_-45_3B-B	0.0004	0.9996
poor_78407847_15_2BB-	0.0000	1.0000
good_11969009_15_3AA	1.0000	0.0000
good_18213488_-30_3AB	1.0000	0.0000
good_20123884_15_3AA-	1.0000	0.0000

poor_79675372_15_3BB-	0.9999	0.0001
good_12971925_45_3A-A-	1.0000	0.0000
poor_78634338_45_2BB	0.0000	1.0000
good_19247486_-15_3AA-	0.9935	0.0065
good_20123884_-30_3AA-	1.0000	0.0000
good_11486813_-30_3AA-	1.0000	0.0000
good_12741796_-45_3AB	1.0000	0.0000
poor_85923677_-45_3B-B	0.0186	0.9814
good_11969009_-45_3AA	1.0000	0.0000
good_17248977_-30_3A-A-	1.0000	0.0000
good_20185605_-45_3AA	1.0000	0.0000
good_12971925_-30_3A-A-	1.0000	0.0000
good_19998381_30_3A-A-	0.9997	0.0003
poor_79496470_-30_1BB	0.0021	0.9979
poor_78085332_-15_2BB	0.0000	1.0000
good_14046542_-15_3A-A-	1.0000	0.0000
poor_82127295_-15_1BB-	0.0000	1.0000
poor_78715791_-15_3BB-	0.0000	1.0000
good_14661384_-15_3A-A-	1.0000	0.0000
good_17017003_0_3A-A-	1.0000	0.0000
poor_79751379_15_2BB	0.0000	1.0000
poor_79318932_-15_2BB-	0.0000	1.0000
poor_79318932_0_2BB-	0.0000	1.0000
poor_84565094_-15_3B-B	0.0000	1.0000
poor_80678895_0_1B-B-	0.0000	1.0000
poor_79146459_-30_2B-B-	0.0001	0.9999
poor_78928905_-15_1BB	0.0000	1.0000
good_17017003_30_3A-A-	1.0000	0.0000
poor_88330925_15_3BB-	0.0000	1.0000
good_11139600_0_3AA	1.0000	0.0000
good_17878975_-45_3AB	1.0000	0.0000
poor_97314024_-30_3B-B	0.0000	1.0000
good_16880651_45_3AB	1.0000	0.0000
good_14411543_30_3AA-	1.0000	0.0000
good_14450819_-15_3A-A-	1.0000	0.0000
poor_79038991_-45_1BB-	0.0000	1.0000
good_16558022_-45_3AB	1.0000	0.0000
poor_78848868_-15_2BB	0.0000	1.0000
good_14853831_45_3A-A-	1.0000	0.0000
poor_79231202_15_3BB-	0.0284	0.9716
poor_88503434_0_3BB-	0.0000	1.0000
good_20185605_15_3AA	1.0000	0.0000
poor_84116513_-30_1BB-	0.0000	1.0000
good_19079428_-15_3A-A-	1.0000	0.0000
good_19678783_45_3AA-	1.0000	0.0000

good_17990992_-15_3A-A-	1.0000	0.0000
poor_84106579_30_1B-B-	0.0000	1.0000
good_18803285_30_3AB	1.0000	0.0000
good_15075835_15_3A-A-	1.0000	0.0000
good_10794293_45_5AA-	0.3079	0.6921
good_15430377_-30_3A-A-	0.9969	0.0031
good_16558022_0_3AB	1.0000	0.0000
good_13117449_15_3A-A-	1.0000	0.0000
good_19030331_-15_3AA	1.0000	0.0000
poor_77206651_-30_2B-B-	0.0032	0.9968
poor_82313942_-15_1-2B-B-	0.0000	1.0000
poor_81678844_0_3BB-	0.0000	1.0000
good_17017003_-15_3A-A-	1.0000	0.0000
poor_77867306_15_1BB	0.0000	1.0000
poor_79545498_15_1-2B-B	0.0000	1.0000
good_12483038_30_3A-A-	1.0000	0.0000
poor_78548643_30_1BB	0.0000	1.0000
good_11183795_45_3AB	1.0000	0.0000
poor_82384146_0_1-2BB	0.0000	1.0000
good_17257791_-15_3AA	1.0000	0.0000
good_14851789_30_3AA	1.0000	0.0000
good_16390514_15_3AA	1.0000	0.0000
poor_77650194_-45_2B-B-	0.0000	1.0000
good_17516331_-45_3AA-	0.9996	0.0004
poor_78911758_-45_1-2BB	0.0000	1.0000
good_18102857_15_3AA-	1.0000	0.0000
poor_77769680_15_2B-B-	0.0000	1.0000
poor_79441878_0_1B-B-	0.0000	1.0000
poor_77433878_30_1BB-	0.0000	1.0000
good_12117875_45_36A-A-	1.0000	0.0000
poor_80876604_-15_1-2B-B	0.0000	1.0000
poor_80530664_-30_1-2B-B	0.0000	1.0000
good_12731906_30_3A-A-	1.0000	0.0000
good_17340290_-15_3A-A-	1.0000	0.0000
good_11132559_-15_3A-A-	1.0000	0.0000
poor_81105911_15_1B-B	0.0000	1.0000
poor_79496470_15_1BB	0.0110	0.9890
poor_77769680_-45_2B-B-	0.0000	1.0000
poor_79861101_-45_2BB-	0.0000	1.0000
good_17392842_-15_3A-A-	1.0000	0.0000
poor_77800673_0_2B-B-	0.0000	1.0000
good_19113441_-15_3A-A	1.0000	0.0000
good_12024489_15_3AA-	1.0000	0.0000
good_20253695_-30_3AB	1.0000	0.0000
poor_84080274_0_1-2B-B	0.0000	1.0000

poor_79867694_45_1BB	0.0000	1.0000
poor_79219063_30_3B-B	0.2807	0.7193
poor_77867306_-30_1BB	0.0000	1.0000
poor_88503434_-45_3BB-	0.0000	1.0000
poor_80296580_-45_3BB-	0.0000	1.0000
good_17300193_-30_3A-A-	1.0000	0.0000
poor_76994692_-30_2BB-	0.0016	0.9984
poor_91094982_-45_3B-B	0.1507	0.8493
poor_77898499_0_1BB	0.0000	1.0000
poor_82168233_-45_1-2B-B	0.0000	1.0000
poor_78407847_-15_2BB-	0.0000	1.0000
poor_80428295_-45_3BB-	0.0000	1.0000
good_10443637_0_3AA-	1.0000	0.0000
good_14005274_-15_3AB	1.0000	0.0000
good_13533711_-30_3AA	1.0000	0.0000
poor_79478757_0_2BB	0.0000	1.0000
poor_77175651_30_2BB	0.0208	0.9792
good_12971925_0_3A-A-	1.0000	0.0000
poor_77059306_-30_2BB	0.0002	0.9998
poor_81526383_30_3BB-	0.0000	1.0000
good_13729954_30_4AA	1.0000	0.0000
good_15961962_-30_3AA	1.0000	0.0000
good_10443637_30_3AA-	1.0000	0.0000
poor_78753524_-15_1-2B-B-	0.0000	1.0000
poor_83001010_-15_3BB-	0.0201	0.9799
good_19334426_30_3A-A-	1.0000	0.0000
poor_78911758_45_1-2BB	0.0000	1.0000
good_12117875_-15_36A-A-	1.0000	0.0000
poor_78341980_-45_3B-B	0.0001	0.9999
poor_77282204_-30_1B-B	0.0000	1.0000
good_10718644_-15_3AA	1.0000	0.0000
good_19942087_-30_3A-A-	1.0000	0.0000
good_18432821_45_3AB	1.0000	0.0000
good_17887898_-45_3A-A-	0.1310	0.8690
good_17709370_-45_3A-A	1.0000	0.0000
good_16286039_30_3A-A-	0.9989	0.0011
good_11743470_-45_3A-A-	1.0000	0.0000
poor_92921469_15_3B-B	0.0000	1.0000
poor_78715791_45_3BB-	0.0000	1.0000
poor_78970539_15_2BB	0.0000	1.0000
good_14450819_45_3A-A-	1.0000	0.0000
good_17257791_0_3AA	1.0000	0.0000
good_18657357_45_3AA-	0.9996	0.0004
good_13117449_45_3A-A-	1.0000	0.0000
poor_92602106_15_3B-B	0.0000	1.0000

good_17591250_45_3AA	1.0000	0.0000
poor_78909238_15_3BB-	0.0002	0.9998
poor_79311557_0_1-2BB-	0.0000	1.0000
poor_92602106_0_3B-B	0.0000	1.0000
poor_77308012_-15_1BB-	0.0000	1.0000
good_19079428_30_3A-A-	1.0000	0.0000
good_17990992_-45_3A-A-	1.0000	0.0000
poor_93221519_45_3B-B	0.0000	1.0000
good_15075835_45_3A-A-	1.0000	0.0000
poor_79545498_45_1-2B-B	0.0000	1.0000
good_19079428_-45_3A-A-	1.0000	0.0000
good_11124729_45_3AA	1.0000	0.0000
poor_85923677_45_3B-B	0.0001	0.9999
good_18781105_-45_3AA	1.0000	0.0000
poor_78715791_0_3BB-	0.0000	1.0000
poor_99187238_15_3B-B	0.0005	0.9995
good_11311361_30_3A-A-	1.0000	0.0000
good_14853831_15_3A-A-	1.0000	0.0000
poor_87749651_-15_3B-B	0.0000	1.0000
poor_78911758_-15_1-2BB	0.0000	1.0000
good_20236076_0_3A-A-	0.0001	0.9999
poor_78634338_-30_2BB	0.0000	1.0000
poor_77769680_45_2B-B-	0.0024	0.9976
poor_79751379_0_2BB	0.0000	1.0000
good_13852248_45_3AB	1.0000	0.0000
good_13858706_0_3AA-	1.0000	0.0000
poor_79545873_45_2BB-	0.0003	0.9997
poor_80310340_-30_1B-B-	0.0000	1.0000
good_14411543_-15_3AA-	1.0000	0.0000
poor_77650194_-15_2B-B-	0.0000	1.0000
poor_97314024_45_3B-B	0.0000	1.0000
good_17017003_-45_3A-A-	0.9965	0.0035
good_19238542_-30_3A-A-	1.0000	0.0000
good_15472679_-45_3AA-	0.9998	0.0002
good_11254311_-45_3A-A	1.0000	0.0000
poor_93221519_-45_3B-B	0.0000	1.0000
good_14661384_-45_3A-A-	1.0000	0.0000
good_10046346_45_3AA	1.0000	0.0000
good_19030331_0_3AA	1.0000	0.0000
good_17591250_-30_3AA	1.0000	0.0000
good_16482386_-45_3AA	1.0000	0.0000
poor_88893942_30_3BB-	0.0769	0.9231
good_11254311_45_3A-A	1.0000	0.0000
good_11922626_0_3A-A-	1.0000	0.0000
good_20106212_15_3AA-	1.0000	0.0000

poor_77800673_15_2B-B-	0.0000	1.0000
good_14046542_-45_3A-A-	1.0000	0.0000
good_11838370_-30_3AB	0.0786	0.9214
good_16004053_-30_3A-A-	1.0000	0.0000
poor_77189946_45_1BB	0.0000	1.0000
good_19536372_0_3AB	0.9929	0.0071
poor_77725120_-45_2B-B	0.0000	1.0000
poor_79478757_-30_2BB	0.0000	1.0000
poor_79365441_0_2BB-	0.0000	1.0000
good_14450819_-45_3A-A-	1.0000	0.0000
good_15453155_30_3AB	1.0000	0.0000
good_11957580_30_3AA-	1.0000	0.0000
poor_77772754_-15_1BB-	0.0000	1.0000
good_19247486_30_3AA-	0.9548	0.0452
poor_77509010_-30_2BB-	0.0001	0.9999
good_12828151_15_3AB	1.0000	0.0000
good_18707760_30_3AA	0.9955	0.0045
good_20106212_-15_3AA-	1.0000	0.0000
good_13261081_15_3AB	1.0000	0.0000
good_16837019_30_3A-A-	1.0000	0.0000
good_11139600_-15_3AA	1.0000	0.0000
poor_78911758_15_1-2BB	0.0001	0.9999
poor_81678844_30_3BB-	0.0000	1.0000
poor_77666018_30_1B-B-	0.0000	1.0000
poor_76994692_15_2BB-	0.0000	1.0000
poor_79311557_-30_1-2BB-	0.0000	1.0000
good_18570412_-30_3AB	1.0000	0.0000
poor_77800673_-15_2B-B-	0.0000	1.0000
poor_79867694_-30_1BB	0.0000	1.0000
good_20123884_0_3AA-	1.0000	0.0000
poor_78334440_45_2BB	0.0000	1.0000
poor_79620727_30_1BB-	0.0000	1.0000
good_10423352_-30_3AA-	1.0000	0.0000
poor_93633156_30_3B-B	0.0000	1.0000
poor_80042420_15_3BB-	0.0000	1.0000
good_14450819_0_3A-A-	1.0000	0.0000
good_18102857_-30_3AA-	1.0000	0.0000
poor_78407847_0_2BB-	0.0000	1.0000
good_14450819_15_3A-A-	1.0000	0.0000
poor_94202441_-15_3B-B	0.1318	0.8682
good_11055829_-45_3AB	0.9997	0.0003
poor_79441878_30_1B-B-	0.0000	1.0000
good_11743470_-15_3A-A-	1.0000	0.0000
poor_78555781_15_2BB	0.0000	1.0000
good_12117875_-45_36A-A-	1.0000	0.0000

good_17516331_0_3AA-	1.0000	0.0000
poor_78164186_-15_3BB-	0.0000	1.0000
good_10163149_0_3AB	1.0000	0.0000
good_17887898_-15_3A-A-	0.0014	0.9986
good_17392842_-45_3A-A-	0.9916	0.0084
good_12741796_15_3AB	1.0000	0.0000
poor_82168233_-15_1-2B-B	0.0000	1.0000
good_13729954_-15_4AA	1.0000	0.0000
good_18935832_-30_3A-A-	1.0000	0.0000
poor_77769680_-15_2B-B-	0.0004	0.9996
poor_79620727_-30_1BB-	0.0000	1.0000
good_11132559_-45_3A-A-	1.0000	0.0000
poor_93031274_-30_3B-B	0.0006	0.9994
good_14046542_0_3A-A-	1.0000	0.0000
poor_77632697_-45_2BB-	0.0000	1.0000
good_17340290_-45_3A-A-	1.0000	0.0000
good_17709370_45_3A-A	1.0000	0.0000
poor_94062269_0_3B-B	0.0000	1.0000
good_16416586_-15_3AB	1.0000	0.0000
good_12483038_-30_3A-A-	1.0000	0.0000
poor_88893942_-45_3BB-	0.0006	0.9994
good_17878975_45_3AB	1.0000	0.0000
good_20205181_30_3A-A-	1.0000	0.0000
good_17257791_15_3AA	1.0000	0.0000
good_19536372_30_3AB	0.5789	0.4211
good_13318962_-30_3AB	1.0000	0.0000
poor_80876604_-45_1-2B-B	0.0000	1.0000
good_12117875_15_36A-A-	1.0000	0.0000
poor_80051850_-45_2BB	0.0000	1.0000
good_19449529_15_3AA-	1.0000	0.0000
good_17340290_0_3A-A-	1.0000	0.0000
poor_79463940_-45_2BB	0.0001	0.9999
good_19141554_45_3AA-	1.0000	0.0000
poor_81678844_-15_3BB-	0.0000	1.0000
good_18803285_-15_3AB	1.0000	0.0000
poor_79161507_-45_1-2BB	0.0000	1.0000
poor_77282204_45_1B-B	0.0000	1.0000
poor_78210494_-30_1BB-	0.0000	1.0000
good_20255380_-15_3A-A-	1.0000	0.0000
good_17003193_-15_3AA	0.9987	0.0013
poor_78284204_45_2B-B	0.0000	1.0000
good_19238542_45_3A-A-	0.9729	0.0271
poor_77632697_45_2BB-	0.0000	1.0000
poor_87749651_30_3B-B	0.0000	1.0000
poor_83666558_-15_1BB-	0.0000	1.0000

good_20205181_-45_3A-A-	1.0000	0.0000
poor_78911758_0_1-2BB	0.0000	1.0000
poor_80051850_15_2BB	0.0000	1.0000
good_17750221_-15_3A-A-	1.0000	0.0000
poor_79038991_45_1BB-	0.0000	1.0000
good_14069738_45_3AA	1.0000	0.0000
poor_78848868_30_2BB	0.0000	1.0000
poor_83582148_45_1B-B-	0.0000	1.0000
poor_80530664_45_1-2B-B	0.0000	1.0000
good_11139600_45_3AA	1.0000	0.0000
good_18781105_45_3AA	1.0000	0.0000
poor_78810664_45_1B-B-	0.0000	1.0000
good_12741796_0_3AB	1.0000	0.0000
poor_83309479_-15_1-2BB	0.0000	1.0000
poor_77390363_-45_1BB-	0.0000	1.0000
good_20253695_0_3AB	1.0000	0.0000
poor_79380966_-45_3B-B	0.0126	0.9874
poor_83626811_-15_3B-B	0.0000	1.0000
good_19942087_30_3A-A-	1.0000	0.0000
poor_79085047_-30_1BB	0.0000	1.0000
poor_79171176_-45_1BB-	0.0000	1.0000
good_14411543_0_3AA-	1.0000	0.0000
good_18240544_0_3A-A-	0.9997	0.0003
poor_77272153_0_2BB-	0.0002	0.9998
poor_77189946_-15_1BB	0.0000	1.0000
good_18707760_-15_3AA	1.0000	0.0000
good_11055829_45_3AB	0.0039	0.9961
good_11183795_0_3AB	1.0000	0.0000
poor_78970539_-45_2BB	0.0000	1.0000
poor_78848868_0_2BB	0.0000	1.0000
poor_77059306_0_2BB	0.0000	1.0000
good_13685820_15_3A-A-	1.0000	0.0000
poor_80384973_0_3BB-	0.0199	0.9801
good_19030331_15_3AA	1.0000	0.0000
poor_78810664_-15_1B-B-	0.0000	1.0000
poor_79861101_30_2BB-	0.0000	1.0000
poor_81406987_0_1-2BB-	0.0000	1.0000
good_14331589_30_3A-A-	1.0000	0.0000
poor_79424795_30_2B-B-	0.0000	1.0000
poor_78814052_15_2BB	0.0000	1.0000
poor_99187238_-45_3B-B	0.0021	0.9979
poor_77650194_30_2B-B-	0.0000	1.0000
good_19536372_-45_3AB	0.3774	0.6226
good_10794293_-45_5AA-	0.0206	0.9794
good_16558022_45_3AB	1.0000	0.0000

poor_77772754_0_1BB-	0.0000	1.0000
poor_91094982_15_3B-B	0.0000	1.0000
poor_78265703_-45_2BB-	0.0002	0.9998
good_10718644_30_3AA	1.0000	0.0000
poor_79171176_45_1BB-	0.0000	1.0000
good_10239739_-45_3AA	1.0000	0.0000
poor_79545873_-45_2BB-	0.0000	1.0000
poor_79478757_30_2BB	0.0000	1.0000
poor_77390363_30_1BB-	0.0000	1.0000
poor_77175651_-30_2BB	0.0000	1.0000
good_12828151_-45_3AB	1.0000	0.0000
poor_80042420_-45_3BB-	0.0000	1.0000
good_17321648_0_3AB	1.0000	0.0000
good_19334426_0_3A-A-	1.0000	0.0000
good_20337692_-45_3AB	1.0000	0.0000
poor_78284204_-30_2B-B	0.0000	1.0000
poor_79968167_-45_1BB	0.0000	1.0000
good_10163149_-30_3AB	1.0000	0.0000
good_17710633_0_3AB	1.0000	0.0000
good_19449529_-45_3AA-	1.0000	0.0000
good_12971925_15_3A-A-	1.0000	0.0000
good_18570412_45_3AB	1.0000	0.0000
good_17454864_-30_3AA-	1.0000	0.0000
good_11302954_-30_3AA-	0.0000	1.0000
good_13158796_-15_3A-A	0.9993	0.0007
good_17003193_45_3AA	0.9994	0.0006
poor_78210494_45_1BB-	0.0000	1.0000
poor_77788406_30_2B-B	0.0000	1.0000
poor_81322712_0_1-2B-B	0.0000	1.0000
good_11922626_45_3A-A-	1.0000	0.0000
poor_77196120_30_1B-B	0.0000	1.0000
good_13198789_45_3AA	1.0000	0.0000
poor_77681626_0_1B-B	0.0000	1.0000
poor_79311557_15_1-2BB-	0.0000	1.0000
good_20337692_15_3AB	1.0000	0.0000
good_17516331_30_3AA-	1.0000	0.0000
poor_77788406_-45_2B-B	0.0000	1.0000
poor_85923677_0_3B-B	0.0000	1.0000
good_13158796_30_3A-A	0.9994	0.0006
good_11124729_-15_3AA	1.0000	0.0000
poor_79431035_-45_1B-B	0.0001	0.9999
good_13858706_30_3AA-	1.0000	0.0000
good_18240544_45_3A-A-	0.9966	0.0034
poor_80678895_30_1B-B-	0.0000	1.0000
poor_81581513_45_3BB-	0.0867	0.9133

poor_96225317_0_3B-B	0.0000	1.0000
poor_83838129_15_3BB-	0.0000	1.0000
good_12483038_0_3A-A-	0.9996	0.0004
good_11666570_45_3-4AA	0.9809	0.0191
poor_77772754_30_1BB-	0.0000	1.0000
poor_93031274_0_3B-B	0.0037	0.9963
poor_81406987_-15_1-2BB-	0.0000	1.0000
good_11666570_-15_3-4AA	1.0000	0.0000
poor_79318932_30_2BB-	0.0000	1.0000
poor_92921469_-30_3B-B	0.0072	0.9928
poor_78233182_-45_1B-B-	0.0000	1.0000
good_20236076_-45_3A-A-	0.0029	0.9971
good_16880651_15_3AB	1.0000	0.0000
poor_77272153_30_2BB-	0.0015	0.9985
good_20106212_-45_3AA-	1.0000	0.0000
good_17878975_-15_3AB	1.0000	0.0000
good_19070722_30_3AA-	1.0000	0.0000
poor_78176117_-30_1-2BB	0.0000	1.0000
poor_79751379_45_2BB	0.0000	1.0000
poor_78928905_-45_1BB	0.0000	1.0000
poor_77772754_-45_1BB-	0.0000	1.0000
good_20106212_45_3AA-	1.0000	0.0000
poor_83453674_30_3BB-	0.4236	0.5764
poor_77004779_0_2BB	0.0000	1.0000
good_17887898_45_3A-A-	0.0000	1.0000
poor_77725120_-15_2B-B	0.0000	1.0000
poor_78085332_-45_2BB	0.0000	1.0000
good_14493269_-30_3AA-	1.0000	0.0000
poor_78251375_30_1B-B	0.0000	1.0000
good_19113441_30_3A-A	1.0000	0.0000
good_17392842_15_3A-A-	1.0000	0.0000
good_15764642_-45_3A-A-	1.0000	0.0000
good_11254311_15_3A-A	1.0000	0.0000
poor_78304592_-30_2B-B-	0.0000	1.0000
good_16390514_45_3AA	1.0000	0.0000
poor_97314024_15_3B-B	0.0000	1.0000
poor_77509010_0_2BB-	0.0000	1.0000
poor_81406987_45_1-2BB-	0.0000	1.0000
poor_82127295_30_1BB-	0.0000	1.0000
good_15472679_-15_3AA-	1.0000	0.0000
good_11254311_-15_3A-A	1.0000	0.0000
poor_78909238_0_3BB-	0.0002	0.9998
poor_79424795_0_2B-B-	0.0000	1.0000
poor_93221519_-15_3B-B	0.0000	1.0000
poor_87749651_-45_3B-B	0.0000	1.0000

good_11183795_15_3AB	1.0000	0.0000
good_12997501_-30_3A-A-	1.0000	0.0000
poor_82745365_-30_3BB-	0.0000	1.0000
good_18240544_-15_3A-A-	0.9995	0.0005
poor_77867306_45_1BB	0.0000	1.0000
poor_81033173_0_1-2BB	0.0000	1.0000
poor_79161507_45_1-2BB	0.0000	1.0000
poor_79545873_15_2BB-	0.0000	1.0000
good_17257791_-45_3AA	1.0000	0.0000
poor_78304592_15_2B-B-	0.0000	1.0000
good_11124729_0_3AA	1.0000	0.0000
good_17641234_-30_3A-A-	1.0000	0.0000
good_14411543_-45_3AA-	1.0000	0.0000
poor_78334440_-30_2BB	0.0000	1.0000
poor_82313942_-30_1-2B-B-	0.0000	1.0000
poor_85923677_15_3B-B	0.0000	1.0000
poor_79675372_-30_3BB-	0.9992	0.0008
poor_99187238_45_3B-B	0.0000	1.0000
good_13117449_0_3A-A-	1.0000	0.0000
good_19030331_-45_3AA	1.0000	0.0000
good_15697722_-30_3AA-	1.0000	0.0000
poor_82153169_30_3B-B	0.0000	1.0000
good_17164399_0_3AB	1.0000	0.0000
poor_92602106_45_3B-B	0.0000	1.0000
poor_78909238_45_3BB-	0.0001	0.9999
good_16558022_-15_3AB	1.0000	0.0000
poor_78848868_-45_2BB	0.0002	0.9998
poor_93221519_15_3B-B	0.0000	1.0000
good_10101873_0_3AA-	1.0000	0.0000
good_20185605_45_3AA	1.0000	0.0000
good_19271216_-30_3A-A-	0.0000	1.0000
good_14164726_15_3A-A-	1.0000	0.0000
good_15481025_30_3A-A	1.0000	0.0000
poor_79867694_15_1BB	0.0000	1.0000
poor_79535552_-30_1BB-C	0.0000	1.0000
poor_79496470_0_1BB	0.0035	0.9965
poor_80058256_0_1BB	0.0000	1.0000
poor_88893942_-15_3BB-	0.9972	0.0028
poor_79496470_45_1BB	0.0000	1.0000
poor_77632697_-15_2BB-	0.0000	1.0000
poor_76986756_30_3BB-	0.0000	1.0000
good_17709370_15_3A-A	0.9613	0.0387
good_11254311_0_3A-A	1.0000	0.0000
poor_79431255_30_1BB	0.0001	0.9999
poor_77898499_-30_1BB	0.0001	0.9999

poor_77519659_30_1BB-	0.0000	1.0000
good_17340290_30_3A-A-	1.0000	0.0000
good_13117449_-30_3A-A-	1.0000	0.0000
good_10423835_-30_3A-A-	1.0000	0.0000
poor_78970539_45_2BB	0.0000	1.0000
good_12024489_0_3AA-	1.0000	0.0000
poor_78164186_-45_3BB-	0.0000	1.0000
poor_83990350_30_1B-B-	0.0007	0.9993
poor_81406987_-30_1-2BB-	0.0000	1.0000
poor_82313942_0_1-2B-B-	0.0000	1.0000
good_20496692_-30_3A-A-	1.0000	0.0000
good_13852248_-30_3AB	1.0000	0.0000
good_10718644_-45_3AA	1.0000	0.0000
poor_78753524_-30_1-2B-B-	0.0000	1.0000
poor_79861101_0_2BB-	0.0000	1.0000
poor_94202441_-45_3B-B	0.0523	0.9477
poor_79441878_-30_1B-B-	0.0000	1.0000
good_18432821_15_3AB	0.9847	0.0153
good_11084942_15_3AB	1.0000	0.0000
poor_80042420_45_3BB-	0.0212	0.9788
poor_79311557_30_1-2BB-	0.0023	0.9977
good_14994218_-15_3A-A-	1.0000	0.0000
good_17937817_45_3A-A-	1.0000	0.0000
good_14851789_-30_3AA	1.0000	0.0000
good_16130067_15_3A-A-	1.0000	0.0000
poor_84116513_30_1BB-	0.0000	1.0000
poor_76994692_45_2BB-	0.0000	1.0000
good_14005274_-45_3AB	1.0000	0.0000
good_13991233_-30_3AA-	1.0000	0.0000
good_14853831_-45_3A-A-	1.0000	0.0000
poor_77433878_0_1BB-	0.0000	1.0000
good_14164726_-15_3A-A-	1.0000	0.0000
good_12097042_-45_3AB	1.0000	0.0000
good_14994218_15_3A-A-	1.0000	0.0000
poor_77004779_45_2BB	0.0001	0.9999
poor_77206651_15_2B-B-	0.0000	1.0000
poor_78651678_-45_1B-B-	0.0006	0.9994
good_20498781_30_3AB	0.9973	0.0027
poor_77320590_-30_3BB-	0.0000	1.0000
poor_77681626_30_1B-B	0.0000	1.0000
good_16130067_-30_3A-A-	1.0000	0.0000
poor_77004779_-30_2BB	0.0000	1.0000
good_17454864_30_3AA-	1.0000	0.0000
good_10232189_-15_3A-A-	1.0000	0.0000
poor_77433878_-30_1BB-	0.0000	1.0000

poor_80310340_45_1B-B-	0.0000	1.0000
poor_77390363_-15_1BB-	0.0000	1.0000
good_13261081_0_3AB	1.0000	0.0000
good_20255380_0_3A-A-	1.0000	0.0000
poor_79380966_-15_3B-B	0.0246	0.9754
poor_82384146_-30_1-2BB	0.0000	1.0000
poor_81105911_-30_1B-B	0.0000	1.0000
poor_83626811_-45_3B-B	0.0000	1.0000
good_17937817_-45_3A-A-	1.0000	0.0000
good_20496692_45_3A-A-	1.0000	0.0000
good_10239739_15_3AA	1.0000	0.0000
good_16880651_-45_3AB	1.0000	0.0000
poor_79171176_-15_1BB-	0.0000	1.0000
good_14046542_15_3A-A-	1.0000	0.0000
good_17164399_45_3AB	1.0000	0.0000
good_19141554_0_3AA-	1.0000	0.0000
good_17990992_45_3A-A-	1.0000	0.0000
good_15102907_-30_3AA	1.0000	0.0000
good_11957580_0_3AA-	1.0000	0.0000
poor_79038991_15_1BB-	0.0000	1.0000
good_20253695_45_3AB	1.0000	0.0000
poor_78251375_0_1B-B	0.0000	1.0000
poor_83199911_-30_3BB-	0.0155	0.9845
poor_77632697_15_2BB-	0.0000	1.0000
good_15102907_0_3AA	1.0000	0.0000
poor_83666558_-45_1BB-	0.0000	1.0000
poor_83990350_-45_1B-B-	0.0001	0.9999
poor_88984960_45_3CB	0.9612	0.0388
poor_81678844_-45_3BB-	0.0000	1.0000
poor_77282204_15_1B-B	0.0000	1.0000
poor_81033173_45_1-2BB	0.0000	1.0000
good_11743470_15_3A-A-	1.0000	0.0000
good_14853831_0_3A-A-	1.0000	0.0000
good_17612946_45_3A-A-	1.0000	0.0000
good_18432821_-15_3AB	0.9973	0.0027
good_10046346_-45_3AA	1.0000	0.0000
good_19890072_15_3AB	1.0000	0.0000
good_14486014_0_3AB	0.9941	0.0059
good_11132559_30_3A-A-	1.0000	0.0000
good_19449529_45_3AA-	1.0000	0.0000
good_19141554_15_3AA-	1.0000	0.0000
poor_83582148_-45_1B-B-	0.0000	1.0000
good_10443637_-30_3AA-	1.0000	0.0000
good_19527698_0_3A-A	1.0000	0.0000
poor_78928905_45_1BB	0.0000	1.0000

poor_79146459_0_2B-B-	0.0000	1.0000
good_16621319_30_3A-A-	1.0000	0.0000
poor_78555781_-30_2BB	0.0000	1.0000
good_10232189_45_3A-A-	1.0000	0.0000
poor_78176117_15_1-2BB	0.0000	1.0000
poor_81581513_15_3BB-	0.0139	0.9861
poor_83838129_45_3BB-	0.0000	1.0000
good_17612946_-45_3A-A-	1.0000	0.0000
poor_80058256_30_1BB	0.0000	1.0000
poor_80428295_30_3BB-	0.0042	0.9958
poor_77196120_0_1B-B	0.0000	1.0000
poor_77788406_-15_2B-B	0.0000	1.0000
good_13831587_30_3AB	1.0000	0.0000
good_19070722_0_3AA-	1.0000	0.0000
poor_78233182_45_1B-B-	0.0000	1.0000
good_10101873_-30_3AA-	1.0000	0.0000
poor_79431035_-15_1B-B	0.0006	0.9994
good_12731906_-45_3A-A-	1.0000	0.0000
poor_78555781_0_2BB	0.0000	1.0000
poor_78210494_15_1BB-	0.0000	1.0000
good_14069738_-45_3AA	1.0000	0.0000
good_17321648_-15_3AB	1.0000	0.0000
good_16433634_-45_3A-A-	1.0000	0.0000
poor_79311557_-15_1-2BB-	0.0001	0.9999
good_18935832_15_3A-A-	1.0000	0.0000
poor_77898499_15_1BB	0.0000	1.0000
poor_78828717_30_2B-B	0.0001	0.9999
poor_77666018_-30_1B-B-	0.0000	1.0000
poor_83990350_0_1B-B-	0.0000	1.0000
poor_77282204_0_1B-B	0.0000	1.0000
good_11969009_-15_3AA	1.0000	0.0000
good_12741796_-15_3AB	1.0000	0.0000
poor_80530664_-45_1-2B-B	0.0000	1.0000
good_19449529_-15_3AA-	1.0000	0.0000
good_12117875_30_36A-A-	1.0000	0.0000
good_11311361_-45_3A-A-	1.0000	0.0000
good_20185605_-15_3AA	1.0000	0.0000
good_13158796_-45_3A-A	0.9156	0.0844
poor_79545873_-15_2BB-	0.0000	1.0000
good_11969009_45_3AA	1.0000	0.0000
poor_78634338_15_2BB	0.0000	1.0000
poor_77655020_0_2BB-	0.0000	1.0000
poor_80042420_-15_3BB-	0.0000	1.0000
poor_88503434_30_3BB-	0.0000	1.0000
good_15764642_30_3A-A-	1.0000	0.0000

poor_91094982_45_3B-B	0.0049	0.9951
poor_78265703_-15_2BB-	0.0000	1.0000
good_13198789_-15_3AA	1.0000	0.0000
good_19334426_-15_3A-A-	1.0000	0.0000
poor_96225317_-30_3B-B	0.0000	1.0000
good_16433634_30_3A-A-	1.0000	0.0000
poor_80678895_-30_1B-B-	0.0000	1.0000
poor_79171176_15_1BB-	0.0000	1.0000
good_10239739_0_3AA	1.0000	0.0000
good_17300193_15_3A-A-	1.0000	0.0000
good_17164399_-30_3AB	1.0000	0.0000
poor_83309479_30_1-2BB	0.0000	1.0000
good_17585937_-30_3AA-	1.0000	0.0000
poor_82168233_-30_1-2B-B	0.0000	1.0000
poor_99187238_-15_3B-B	0.0000	1.0000
good_12828151_0_3AB	1.0000	0.0000
good_10794293_-15_5AA-	0.0027	0.9973
poor_81581513_0_3BB-	0.9565	0.0435
good_10163149_30_3AB	1.0000	0.0000
good_14069738_15_3AA	1.0000	0.0000
poor_83990350_-15_1B-B-	0.0000	1.0000
poor_80051850_45_2BB	0.0000	1.0000
good_18657357_-15_3AA-	0.9999	0.0001
good_11139600_15_3AA	1.0000	0.0000
good_18781105_15_3AA	1.0000	0.0000
good_17003193_-45_3AA	1.0000	0.0000
poor_93633156_-30_3B-B	0.0000	1.0000
poor_78869483_30_3B-B	0.0000	1.0000
good_15102907_15_3AA	1.0000	0.0000
good_13858706_-30_3AA-	1.0000	0.0000
good_18803285_-45_3AB	1.0000	0.0000
poor_79380966_45_3B-B	0.0020	0.9980
poor_83582148_-15_1B-B-	0.0000	1.0000
poor_76986756_-45_3BB-	0.0000	1.0000
poor_79675372_0_3BB-	0.9999	0.0001
good_11743470_45_3A-A-	1.0000	0.0000
poor_80051850_-15_2BB	0.0000	1.0000
poor_81033173_15_1-2BB	0.0000	1.0000
good_16390514_-30_3AA	1.0000	0.0000
good_17612946_15_3A-A-	0.5081	0.4919
poor_78928905_0_1BB	0.0000	1.0000
poor_79463940_-15_2BB	0.0000	1.0000
poor_79424795_-30_2B-B-	0.0000	1.0000
poor_79431035_30_1B-B	0.0000	1.0000
good_11302954_0_3AA-	0.0000	1.0000

poor_79231202_-45_3BB-	0.3371	0.6629
good_14952697_15_3AA-	1.0000	0.0000
good_10232189_-45_3A-A-	1.0000	0.0000
good_19030331_45_3AA	1.0000	0.0000
poor_80310340_15_1B-B-	0.0000	1.0000
poor_96225317_30_3B-B	0.0000	1.0000
poor_83838129_-15_3BB-	0.0000	1.0000
good_15481025_-15_3A-A	1.0000	0.0000
poor_84565094_30_3B-B	0.0000	1.0000
good_17321648_30_3AB	1.0000	0.0000
poor_78651678_-15_1B-B-	0.0000	1.0000
good_11055829_15_3AB	0.9955	0.0045
good_18707760_-45_3AA	1.0000	0.0000
poor_77189946_-45_1BB	0.0000	1.0000
good_10101873_15_3AA-	1.0000	0.0000
good_14486014_30_3AB	0.9885	0.0115
poor_80384973_30_3BB-	0.0056	0.9944
good_14994218_45_3A-A-	1.0000	0.0000
poor_77206651_45_2B-B-	0.4670	0.5330
poor_80058256_-30_1BB	0.0000	1.0000
good_11142866_-30_3A-A-	1.0000	0.0000
poor_78970539_-15_2BB	0.0000	1.0000
poor_79545498_30_1-2B-B	0.0000	1.0000
poor_79535552_30_1BB-C	0.0000	1.0000
poor_94994695_15_3B-B	0.0000	1.0000
poor_82153169_-45_3B-B	0.0002	0.9998
poor_78909238_-15_3BB-	0.0000	1.0000
good_14046542_45_3A-A-	1.0000	0.0000
good_14005274_30_3AB	1.0000	0.0000
good_17990992_15_3A-A-	1.0000	0.0000
good_20496692_15_3A-A-	1.0000	0.0000
poor_92602106_-45_3B-B	0.0000	1.0000
good_19527698_15_3A-A	1.0000	0.0000
good_17937817_-15_3A-A-	1.0000	0.0000
poor_78548643_-30_1BB	0.0000	1.0000
poor_77681626_-30_1B-B	0.0000	1.0000
good_10423352_15_3AA-	1.0000	0.0000
good_19890072_-30_3AB	1.0000	0.0000
poor_79463940_30_2BB	0.0000	1.0000
poor_79038991_0_1BB-	0.0000	1.0000
good_14952697_-30_3AA-	1.0000	0.0000
good_13533711_30_3AA	1.0000	0.0000
poor_85923677_-15_3B-B	0.0000	1.0000
poor_94202441_30_3B-B	0.0000	1.0000
poor_79431255_-30_1BB	0.0000	1.0000

good_17003193_15_3AA	0.9997	0.0003
poor_80310340_0_1B-B-	0.0000	1.0000
good_18570412_15_3AB	1.0000	0.0000
poor_78304592_0_2B-B-	0.0000	1.0000
poor_78407847_45_2BB-	0.0000	1.0000
good_20123884_45_3AA-	1.0000	0.0000
good_13831587_-30_3AB	0.9835	0.0165
good_13318962_30_3AB	1.0000	0.0000
good_13685820_-30_3A-A-	1.0000	0.0000
good_10423835_30_3A-A-	0.9999	0.0001
poor_77272153_-30_2BB-	0.0001	0.9999
poor_79675372_45_3BB-	0.9961	0.0039
good_15961962_30_3AA	1.0000	0.0000
poor_81581513_-30_3BB-	0.0011	0.9989
good_20337692_-15_3AB	1.0000	0.0000
good_17710633_-30_3AB	1.0000	0.0000
good_11311361_-15_3A-A-	1.0000	0.0000
good_12828151_-15_3AB	1.0000	0.0000
good_19247486_-45_3AA-	1.0000	0.0000
good_13261081_-30_3AB	0.9911	0.0089
good_19527698_-15_3A-A	1.0000	0.0000
poor_77320590_15_3BB-	0.0000	1.0000
poor_79365441_45_2BB-	0.0000	1.0000
poor_77655020_15_2BB-	0.0002	0.9998
good_10239739_-15_3AA	1.0000	0.0000
poor_82384146_30_1-2BB	0.0000	1.0000
poor_93031274_30_3B-B	0.0207	0.9793
good_15430377_30_3A-A-	1.0000	0.0000
good_12097042_30_3AB	1.0000	0.0000
good_17300193_45_3A-A-	1.0000	0.0000
poor_79219063_-15_3B-B	0.0000	1.0000
poor_80530664_-15_1-2B-B	0.0000	1.0000
poor_80876604_-30_1-2B-B	0.0000	1.0000
good_17710633_30_3AB	1.0000	0.0000
good_16558022_15_3AB	1.0000	0.0000
good_19271216_30_3A-A-	0.0000	1.0000
poor_97314024_0_3B-B	0.0000	1.0000
good_19334426_-45_3A-A-	1.0000	0.0000
good_17990992_0_3A-A-	1.0000	0.0000
good_19536372_-15_3AB	0.9959	0.0041
poor_78814052_45_2BB	0.0000	1.0000
poor_78651678_30_1B-B-	0.0000	1.0000
good_13991233_30_3AA-	1.0000	0.0000
poor_81526383_-30_3BB-	0.0000	1.0000
good_11302954_15_3AA-	0.0000	1.0000

good_17612946_-15_3A-A-	0.9957	0.0043
poor_77655020_-15_2BB-	0.0025	0.9975
poor_78176117_45_1-2BB	0.0000	1.0000
poor_77852541_-15_2BB-	0.0000	1.0000
poor_94994695_-30_3B-B	0.0035	0.9965
poor_79311557_-45_1-2BB-	0.0000	1.0000
good_10232189_15_3A-A-	1.0000	0.0000
poor_98690041_-15_3B-B	0.0000	1.0000
good_17300193_0_3A-A-	1.0000	0.0000
poor_83666558_45_1BB-	0.0000	1.0000
good_16390514_0_3AA	1.0000	0.0000
poor_83626811_15_3B-B	0.0000	1.0000
good_12997501_30_3A-A-	1.0000	0.0000
poor_79365441_-30_2BB-	0.0000	1.0000
good_16621319_-30_3A-A-	1.0000	0.0000
good_16286039_-30_3A-A-	0.9360	0.0640
good_19678783_0_3AA-	1.0000	0.0000
poor_94062269_15_3B-B	0.0000	1.0000
poor_77519659_-45_1BB-	0.0002	0.9998
good_15697722_30_3AA-	1.0000	0.0000
good_12117875_-30_36A-A-	1.0000	0.0000
good_18935832_45_3A-A-	1.0000	0.0000
poor_83663432_0_1-2BB-	0.0012	0.9988
good_20337692_45_3AB	1.0000	0.0000
good_12731906_-15_3A-A-	0.9999	0.0001
good_11124729_-45_3AA	1.0000	0.0000
good_16433634_-15_3A-A-	1.0000	0.0000
good_11132559_0_3A-A-	1.0000	0.0000
poor_77852541_15_2BB-	0.0000	1.0000
good_15472679_15_3AA-	1.0000	0.0000
poor_85900848_0_3B-B	0.0000	1.0000
poor_78233182_15_1B-B-	0.0000	1.0000
poor_77175651_0_2BB	0.0002	0.9998
good_11183795_-30_3AB	1.0000	0.0000
good_13198789_15_3AA	1.0000	0.0000
poor_91094982_0_3B-B	0.0124	0.9876
poor_78304592_45_2B-B-	0.0034	0.9966
good_17516331_-15_3AA-	1.0000	0.0000
good_18102857_45_3AA-	1.0000	0.0000
good_18240544_-45_3A-A-	1.0000	0.0000
poor_82313942_-45_1-2B-B-	0.0000	1.0000
poor_79161507_15_1-2BB	0.0000	1.0000
good_13852248_15_3AB	1.0000	0.0000
good_14493269_30_3AA-	1.0000	0.0000
good_19141554_-30_3AA-	1.0000	0.0000

good_11957580_-30_3AA-	1.0000	0.0000
good_19079428_0_3A-A-	1.0000	0.0000
poor_77725120_30_2B-B	0.0000	1.0000
good_11124729_15_3AA	1.0000	0.0000
good_18781105_-15_3AA	1.0000	0.0000
good_10794293_15_5AA-	0.8315	0.1685
poor_77650194_0_2B-B-	0.0000	1.0000
good_16416586_30_3AB	1.0000	0.0000
good_19113441_0_3A-A	1.0000	0.0000
good_14486014_-30_3AB	0.9993	0.0007
good_13991233_0_3AA-	1.0000	0.0000
poor_81406987_15_1-2BB-	0.0000	1.0000
poor_79231202_45_3BB-	0.0004	0.9996
good_17591250_15_3AA	1.0000	0.0000
good_19678783_15_3AA-	1.0000	0.0000
good_11486813_30_3AA-	1.0000	0.0000
poor_88330925_45_3BB-	0.0000	1.0000
good_16482386_30_3AA	1.0000	0.0000
good_12828151_45_3AB	1.0000	0.0000
poor_79038991_-15_1BB-	0.0000	1.0000
poor_77867306_0_1BB	0.0000	1.0000
poor_79318932_-45_2BB-	0.0011	0.9989
poor_84565094_-45_3B-B	0.0002	0.9998
poor_78233182_-15_1B-B-	0.0000	1.0000
good_20236076_-15_3A-A-	0.0001	0.9999
good_11666570_0_3-4AA	1.0000	0.0000
good_15764642_-15_3A-A-	1.0000	0.0000
poor_79146459_30_2B-B-	0.0000	1.0000
good_17392842_45_3A-A-	1.0000	0.0000
poor_80296580_30_3BB-	0.0001	0.9999
poor_77189946_15_1BB	0.0000	1.0000
poor_80530664_30_1-2B-B	0.0000	1.0000
good_20236076_30_3A-A-	0.6602	0.3398
good_17887898_15_3A-A-	0.0000	1.0000
good_10046346_15_3AA	1.0000	0.0000
good_12024489_-30_3AA-	1.0000	0.0000
poor_82127295_-45_1BB-	0.0000	1.0000
poor_78715791_-45_3BB-	0.0000	1.0000
good_16482386_-15_3AA	1.0000	0.0000
poor_78753524_-45_1-2B-B-	0.0000	1.0000
poor_92921469_45_3B-B	0.0000	1.0000
poor_78715791_15_3BB-	0.0001	0.9999
good_18657357_15_3AA-	0.7584	0.2416
good_19998381_-30_3A-A-	0.1222	0.8778
poor_83199911_30_3BB-	0.0000	1.0000

poor_83453674_-30_3BB-	0.0000	1.0000
poor_88984960_0_3CB	1.0000	0.0000
good_11142866_30_3A-A-	1.0000	0.0000
good_17709370_-15_3A-A	1.0000	0.0000
good_11055829_-15_3AB	1.0000	0.0000
good_14853831_-15_3A-A-	1.0000	0.0000
poor_79219063_0_3B-B	0.0003	0.9997
good_14164726_-45_3A-A-	1.0000	0.0000
poor_83001010_-45_3BB-	0.9399	0.0601
good_16130067_45_3A-A-	1.0000	0.0000
poor_78341980_-15_3B-B	0.0000	1.0000
good_11922626_-30_3A-A-	1.0000	0.0000
poor_78251375_-30_1B-B	0.0000	1.0000
good_14331589_-30_3A-A-	1.0000	0.0000
good_14994218_-45_3A-A-	1.0000	0.0000
good_17937817_15_3A-A-	1.0000	0.0000
good_11139600_-45_3AA	1.0000	0.0000
poor_77189946_0_1BB	0.0000	1.0000
poor_78334440_15_2BB	0.0000	1.0000
poor_93633156_0_3B-B	0.0000	1.0000
poor_80296580_-15_3BB-	0.0000	1.0000
poor_91094982_-15_3B-B	0.3159	0.6841
good_17257791_45_3AA	1.0000	0.0000
good_16004053_0_3A-A-	1.0000	0.0000
poor_78176117_0_1-2BB	0.0000	1.0000
good_18213488_30_3AB	1.0000	0.0000
poor_80428295_-15_3BB-	0.0000	1.0000
poor_78407847_-45_2BB-	0.0000	1.0000
good_16004053_30_3A-A-	1.0000	0.0000
poor_85900848_30_3B-B	0.0000	1.0000
good_19113441_-45_3A-A	1.0000	0.0000
good_12024489_45_3AA-	1.0000	0.0000
good_14164726_45_3A-A-	1.0000	0.0000
good_16416586_-45_3AB	1.0000	0.0000
good_14005274_0_3AB	1.0000	0.0000
good_17878975_15_3AB	1.0000	0.0000
poor_78210494_0_1BB-	0.0000	1.0000
poor_88503434_-15_3BB-	0.0000	1.0000
good_17248977_30_3A-A-	1.0000	0.0000
good_15453155_-30_3AB	1.0000	0.0000
poor_94062269_-30_3B-B	0.0000	1.0000
poor_81105911_45_1B-B	0.0000	1.0000
poor_79861101_-15_2BB-	0.0000	1.0000
poor_78814052_-30_2BB	0.0000	1.0000
good_16837019_-30_3A-A-	1.0000	0.0000

good_15961962_0_3AA	1.0000	0.0000
good_13729954_-45_4AA	1.0000	0.0000
good_12741796_45_3AB	1.0000	0.0000
good_15075835_-30_3A-A-	1.0000	0.0000
poor_79231202_0_3BB-	0.0012	0.9988
poor_77308012_30_1BB-	0.0000	1.0000
poor_77196120_-30_1B-B	0.0000	1.0000
poor_80428295_15_3BB-	0.0000	1.0000
good_17003193_0_3AA	0.9961	0.0039
poor_77788406_-30_2B-B	0.0000	1.0000
good_17516331_45_3AA-	1.0000	0.0000
poor_79431035_-30_1B-B	0.0001	0.9999
good_10101873_-15_3AA-	1.0000	0.0000
good_13158796_45_3A-A	0.9999	0.0001
good_20205181_15_3A-A-	1.0000	0.0000
poor_79867694_30_1BB	0.0000	1.0000
good_17300193_-45_3A-A-	1.0000	0.0000
poor_78210494_30_1BB-	0.0000	1.0000
poor_77788406_45_2B-B	0.0000	1.0000
good_20253695_-45_3AB	0.9438	0.0562
poor_77867306_-45_1BB	0.0000	1.0000
poor_78828717_15_2B-B	0.0003	0.9997
good_12483038_-15_3A-A-	1.0000	0.0000
poor_76994692_0_2BB-	0.0000	1.0000
poor_77772754_45_1BB-	0.0000	1.0000
good_10443637_-15_3AA-	1.0000	0.0000
poor_94994695_0_3B-B	0.0003	0.9997
poor_79318932_45_2BB-	0.0000	1.0000
good_12731906_45_3A-A-	1.0000	0.0000
poor_77898499_-15_1BB	0.0000	1.0000
good_13858706_45_3AA-	1.0000	0.0000
poor_77769680_-30_2B-B-	0.0000	1.0000
poor_79431255_15_1BB	0.0000	1.0000
poor_81581513_30_3BB-	0.0022	0.9978
good_18935832_-15_3A-A-	1.0000	0.0000
poor_80321976_0_1B-B-	0.0000	1.0000
poor_78265703_-30_2BB-	0.0000	1.0000
poor_96225317_-15_3B-B	0.0000	1.0000
good_17750221_0_3A-A-	1.0000	0.0000
good_17887898_-30_3A-A-	0.0136	0.9864
good_16286039_45_3A-A-	0.9972	0.0028
good_20185605_0_3AA	1.0000	0.0000
good_19942087_-45_3A-A-	1.0000	0.0000
poor_79171176_30_1BB-	0.0001	0.9999
good_13852248_-15_3AB	1.0000	0.0000

good_17585937_-15_3AA-	0.9999	0.0001
poor_79441878_15_1B-B-	0.0000	1.0000
good_11743470_-30_3A-A-	1.0000	0.0000
poor_83666558_0_1BB-	0.0000	1.0000
good_10794293_-30_5AA-	0.0008	0.9992
good_14450819_30_3A-A-	1.0000	0.0000
poor_99187238_-30_3B-B	0.0000	1.0000
poor_81322712_-30_1-2B-B	0.0003	0.9997
good_18432821_30_3AB	1.0000	0.0000
poor_78334440_0_2BB	0.0000	1.0000
good_19449529_-30_3AA-	1.0000	0.0000
good_11084942_30_3AB	1.0000	0.0000
good_13729954_45_4AA	1.0000	0.0000
poor_78753524_15_1-2B-B-	0.0000	1.0000
good_17454864_-45_3AA-	1.0000	0.0000
good_14851789_-15_3AA	1.0000	0.0000
good_20496692_0_3A-A-	1.0000	0.0000
good_11302954_-45_3AA-	0.0000	1.0000
poor_77175651_45_2BB	0.0094	0.9906
good_19334426_45_3A-A-	1.0000	0.0000
poor_79545873_-30_2BB-	0.0000	1.0000
poor_78548643_0_1BB	0.0000	1.0000
good_13533711_-45_3AA	1.0000	0.0000
poor_77390363_45_1BB-	0.0000	1.0000
poor_77800673_-30_2B-B-	0.0000	1.0000
poor_88503434_15_3BB-	0.0000	1.0000
poor_77666018_15_1B-B-	0.0000	1.0000
poor_77059306_-45_2BB	0.0016	0.9984
poor_80042420_-30_3BB-	0.0000	1.0000
poor_78284204_-45_2B-B	0.0003	0.9997
poor_78911758_30_1-2BB	0.0000	1.0000
good_16837019_15_3A-A-	1.0000	0.0000
poor_79380966_-30_3B-B	0.0332	0.9668
poor_83663432_-45_1-2BB-	0.0000	1.0000
poor_77390363_-30_1BB-	0.0000	1.0000
poor_79171176_-30_1BB-	0.0000	1.0000
poor_78284204_0_2B-B	0.0000	1.0000
poor_79146459_-45_2B-B-	0.0000	1.0000
good_16880651_30_3AB	1.0000	0.0000
poor_81105911_-15_1B-B	0.0000	1.0000
poor_84080274_30_1-2B-B	0.0000	1.0000
good_17878975_-30_3AB	1.0000	0.0000
good_17017003_45_3A-A-	1.0000	0.0000
poor_82313942_45_1-2B-B-	0.0000	1.0000
good_17641234_0_3A-A-	1.0000	0.0000

good_16004053_-15_3A-A-	1.0000	0.0000
poor_77320590_-15_3BB-	0.0000	1.0000
poor_79463940_0_2BB	0.0001	0.9999
poor_77800673_30_2B-B-	0.0000	1.0000
good_18657357_0_3AA-	0.8919	0.1081
poor_79861101_45_2BB-	0.0000	1.0000
poor_79496470_-45_1BB	0.0000	1.0000
poor_79161507_0_1-2BB	0.0000	1.0000
poor_77681626_15_1B-B	0.0000	1.0000
good_17454864_15_3AA-	1.0000	0.0000
poor_77433878_-15_1BB-	0.0000	1.0000
good_12483038_45_3A-A-	1.0000	0.0000
good_19238542_-15_3A-A-	1.0000	0.0000
poor_77282204_30_1B-B	0.0000	1.0000
good_14851789_45_3AA	1.0000	0.0000
poor_78210494_-45_1BB-	0.0000	1.0000
poor_78548643_45_1BB	0.0000	1.0000
poor_77650194_-30_2B-B-	0.0000	1.0000
good_11183795_30_3AB	1.0000	0.0000
poor_80310340_-15_1B-B-	0.0000	1.0000
good_12997501_0_3A-A-	1.0000	0.0000
good_19141554_30_3AA-	1.0000	0.0000
poor_78334440_-15_2BB	0.0000	1.0000
poor_78911758_-30_1-2BB	0.0000	1.0000
poor_80876604_45_1-2B-B	0.0000	1.0000
good_14853831_30_3A-A-	1.0000	0.0000
good_11311361_15_3A-A-	1.0000	0.0000
poor_79038991_30_1BB-	0.0000	1.0000
good_18803285_45_3AB	1.0000	0.0000
good_13685820_0_3A-A-	1.0000	0.0000
poor_92921469_0_3B-B	0.0000	1.0000
poor_84080274_-45_1-2B-B	0.0000	1.0000
poor_83199911_-15_3BB-	0.0001	0.9999
good_10794293_0_5AA-	0.0464	0.9536
good_17878975_0_3AB	1.0000	0.0000
poor_84106579_45_1B-B-	0.0000	1.0000
good_19079428_15_3A-A-	1.0000	0.0000
good_16558022_-30_3AB	1.0000	0.0000
poor_77206651_-45_2B-B-	0.0000	1.0000
good_17585937_0_3AA-	1.0000	0.0000
poor_77632697_30_2BB-	0.0000	1.0000
good_15430377_-45_3A-A-	1.0000	0.0000
poor_87749651_45_3B-B	0.0000	1.0000
poor_76986756_15_3BB-	0.0000	1.0000
poor_93031274_-45_3B-B	0.6155	0.3845

poor_77632697_-30_2BB-	0.0000	1.0000
good_11666570_-30_3-4AA	1.0000	0.0000
good_17709370_30_3A-A	1.0000	0.0000
poor_80678895_15_1B-B-	0.0000	1.0000
poor_77519659_15_1BB-	0.0002	0.9998
poor_78555781_-15_2BB	0.0000	1.0000
poor_88984960_-45_3CB	0.9309	0.0691
poor_77852541_0_2BB-	0.0000	1.0000
poor_80058256_15_1BB	0.0000	1.0000
good_15481025_15_3A-A	1.0000	0.0000
good_17641234_45_3A-A-	1.0000	0.0000
good_13831587_15_3AB	0.9998	0.0002
poor_78085332_45_2BB	0.0000	1.0000
good_13852248_0_3AB	1.0000	0.0000
good_17321648_-30_3AB	1.0000	0.0000
poor_79545498_-45_1-2B-B	0.0000	1.0000
poor_93221519_0_3B-B	0.0000	1.0000
poor_77898499_30_1BB	0.0000	1.0000
good_17750221_45_3A-A-	1.0000	0.0000
poor_87749651_0_3B-B	0.0000	1.0000
poor_88893942_-30_3BB-	0.0056	0.9944
good_11969009_-30_3AA	0.9999	0.0001
good_12741796_-30_3AB	1.0000	0.0000
good_18102857_-45_3AA-	1.0000	0.0000
good_12971925_30_3A-A-	1.0000	0.0000
poor_77308012_0_1BB-	0.0000	1.0000
good_20185605_-30_3AA	1.0000	0.0000
good_10423352_-45_3AA-	1.0000	0.0000
good_18213488_-45_3AB	1.0000	0.0000
poor_81322712_30_1-2B-B	0.0006	0.9994
poor_84116513_15_1BB-	0.0000	1.0000
good_11084942_0_3AB	1.0000	0.0000
poor_81678844_45_3BB-	0.0005	0.9995
good_17248977_-45_3A-A-	1.0000	0.0000
good_12971925_-45_3A-A-	1.0000	0.0000
poor_78634338_30_2BB	0.0000	1.0000
good_19998381_45_3A-A-	1.0000	0.0000
good_13991233_-15_3AA-	1.0000	0.0000
good_13198789_-30_3AA	0.9999	0.0001
poor_77059306_45_2BB	0.0001	0.9999
poor_79171176_0_1BB-	0.0000	1.0000
good_17164399_-15_3AB	1.0000	0.0000
poor_77650194_15_2B-B-	0.0000	1.0000
poor_79424795_15_2B-B-	0.0000	1.0000
poor_82168233_45_1-2B-B	0.0000	1.0000

good_14331589_15_3A-A-	1.0000	0.0000
good_19271216_0_3A-A-	0.0000	1.0000
poor_83453674_15_3BB-	0.0013	0.9987
poor_77725120_-30_2B-B	0.0000	1.0000
poor_78810664_-30_1B-B-	0.0000	1.0000
good_13685820_30_3A-A-	1.0000	0.0000
good_20498781_15_3AB	1.0000	0.0000
poor_79620727_0_1BB-	0.0000	1.0000
poor_78251375_15_1B-B	0.0000	1.0000
good_18432821_0_3AB	0.9987	0.0013
good_11838370_45_3AB	0.9978	0.0022
good_14493269_-15_3AA-	1.0000	0.0000
good_19113441_15_3A-A	1.0000	0.0000
good_11254311_30_3A-A	1.0000	0.0000
poor_77004779_-15_2BB	0.0000	1.0000
poor_88893942_45_3BB-	0.1494	0.8506
good_19942087_15_3A-A-	1.0000	0.0000
good_14069738_0_3AA	1.0000	0.0000
poor_92921469_-15_3B-B	0.0000	1.0000
good_10239739_30_3AA	1.0000	0.0000
poor_77272153_15_2BB-	0.0001	0.9999
good_19070722_15_3AA-	1.0000	0.0000
poor_81033173_-45_1-2BB	0.0001	0.9999
good_19247486_45_3AA-	0.9992	0.0008
poor_77509010_-45_2BB-	0.0008	0.9992
poor_83309479_-30_1-2BB	0.0000	1.0000
good_11957580_45_3AA-	1.0000	0.0000
good_20498781_-45_3AB	1.0000	0.0000
good_15102907_-15_3AA	1.0000	0.0000
good_14661384_45_3A-A-	1.0000	0.0000
good_10718644_0_3AA	1.0000	0.0000
poor_78634338_0_2BB	0.0000	1.0000
good_12117875_0_36A-A-	1.0000	0.0000
poor_79675372_-15_3BB-	0.9945	0.0055
poor_85923677_30_3B-B	0.0001	0.9999
poor_82153169_15_3B-B	0.0000	1.0000
good_15697722_-15_3AA-	1.0000	0.0000
good_17750221_-30_3A-A-	1.0000	0.0000
poor_79751379_-45_2BB	0.0000	1.0000
good_20255380_45_3A-A-	1.0000	0.0000
poor_77788406_0_2B-B	0.0000	1.0000
poor_93221519_30_3B-B	0.0000	1.0000
good_20255380_-30_3A-A-	1.0000	0.0000
good_17454864_0_3AA-	1.0000	0.0000
poor_97314024_30_3B-B	0.0000	1.0000

good_15472679_-30_3AA-	1.0000	0.0000
good_11254311_-30_3A-A	1.0000	0.0000
poor_93221519_-30_3B-B	0.0000	1.0000
poor_79085047_45_1BB	0.0000	1.0000
poor_82127295_15_1BB-	0.0000	1.0000
good_18432821_-30_3AB	1.0000	0.0000
poor_77390363_0_1BB-	0.0001	0.9999
good_18707760_0_3AA	0.9998	0.0002
good_19890072_30_3AB	1.0000	0.0000
poor_82745365_-15_3BB-	0.0000	1.0000
poor_82745365_0_3BB-	0.0000	1.0000
poor_84106579_-45_1B-B-	0.0000	1.0000
poor_79545873_30_2BB-	0.0004	0.9996
good_14661384_0_3A-A-	1.0000	0.0000
good_12971925_-15_3A-A-	1.0000	0.0000
good_13533711_15_3AA	1.0000	0.0000
good_19998381_15_3A-A-	0.9972	0.0028
good_17248977_-15_3A-A-	1.0000	0.0000
good_13198789_0_3AA	1.0000	0.0000
good_10163149_-45_3AB	1.0000	0.0000
good_10443637_45_3AA-	1.0000	0.0000
good_17248977_0_3A-A-	1.0000	0.0000
poor_79431255_-15_1BB	0.0000	1.0000
good_17003193_30_3AA	0.9993	0.0007
poor_78341980_-30_3B-B	0.0000	1.0000
poor_77282204_-45_1B-B	0.0000	1.0000
good_18570412_30_3AB	1.0000	0.0000
poor_82153169_0_3B-B	0.0000	1.0000
poor_79478757_45_2BB	0.0000	1.0000
good_13831587_-15_3AB	0.0547	0.9453
good_13318962_15_3AB	1.0000	0.0000
poor_78251375_-15_1B-B	0.0000	1.0000
poor_81526383_45_3BB-	0.0000	1.0000
good_12828151_-30_3AB	1.0000	0.0000
poor_77175651_-45_2BB	0.0000	1.0000
good_20337692_-30_3AB	1.0000	0.0000
good_17710633_-15_3AB	1.0000	0.0000
poor_78715791_30_3BB-	0.0000	1.0000
good_13261081_-15_3AB	1.0000	0.0000
poor_79424795_45_2B-B-	0.0000	1.0000
good_12097042_15_3AB	1.0000	0.0000
good_10239739_-30_3AA	1.0000	0.0000
good_10718644_45_3AA	1.0000	0.0000
poor_77650194_45_2B-B-	0.0000	1.0000
good_18657357_30_3AA-	0.9989	0.0011

poor_83199911_15_3BB-	0.0003	0.9997
poor_83453674_-15_3BB-	0.0000	1.0000
good_17710633_15_3AB	1.0000	0.0000
good_17709370_-30_3A-A	1.0000	0.0000
poor_88893942_0_3BB-	0.8326	0.1674
good_16558022_30_3AB	1.0000	0.0000
good_13729954_0_4AA	1.0000	0.0000
good_19536372_-30_3AB	0.9943	0.0057
good_18240544_30_3A-A-	0.9991	0.0009
good_11055829_0_3AB	0.9989	0.0011
poor_79545498_-15_1-2B-B	0.0000	1.0000
poor_94062269_-15_3B-B	0.0000	1.0000
poor_79861101_-30_2BB-	0.0000	1.0000
poor_80678895_45_1B-B-	0.0000	1.0000
good_14164726_0_3A-A-	1.0000	0.0000
good_11666570_30_3-4AA	1.0000	0.0000
good_13533711_0_3AA	1.0000	0.0000
poor_81526383_0_3BB-	0.0000	1.0000
poor_77308012_15_1BB-	0.0000	1.0000
poor_77196120_-15_1B-B	0.0000	1.0000
good_11922626_30_3A-A-	1.0000	0.0000
poor_80296580_-30_3BB-	0.0002	0.9998
good_17750221_15_3A-A-	1.0000	0.0000
poor_76994692_-45_2BB-	0.0000	1.0000
poor_91094982_-30_3B-B	0.0182	0.9818
poor_85900848_15_3B-B	0.0000	1.0000
poor_80428295_-30_3BB-	0.0000	1.0000
good_17641234_15_3A-A-	1.0000	0.0000
poor_79535552_0_1BB-C	0.0000	1.0000
good_13198789_30_3AA	1.0000	0.0000
good_11183795_-15_3AB	1.0000	0.0000
poor_88503434_-30_3BB-	0.0000	1.0000
poor_79219063_45_3B-B	0.0047	0.9953
good_10163149_15_3AB	1.0000	0.0000
poor_78848868_45_2BB	0.0000	1.0000
good_19238542_30_3A-A-	0.9988	0.0012
poor_79431035_0_1B-B	0.0000	1.0000
good_14069738_30_3AA	1.0000	0.0000
poor_77725120_15_2B-B	0.0000	1.0000
poor_84092585_0_1BB-	0.0000	1.0000
good_10794293_30_5AA-	0.7742	0.2258
good_20255380_15_3A-A-	1.0000	0.0000
good_18781105_30_3AA	1.0000	0.0000
good_11139600_30_3AA	1.0000	0.0000
poor_83582148_30_1B-B-	0.0000	1.0000

good_10423352_0_3AA-	1.0000	0.0000
poor_84116513_-45_1BB-	0.0000	1.0000
good_19678783_30_3AA-	1.0000	0.0000
good_15102907_30_3AA	1.0000	0.0000
good_17516331_-30_3AA-	1.0000	0.0000
poor_84106579_-15_1B-B-	0.0000	1.0000
poor_99187238_0_3B-B	0.0001	0.9999
poor_80051850_-30_2BB	0.0000	1.0000
good_16390514_-15_3AA	1.0000	0.0000
poor_79161507_-30_1-2BB	0.0000	1.0000
good_14493269_15_3AA-	1.0000	0.0000
good_10423835_0_3A-A-	1.0000	0.0000
poor_78341980_0_3B-B	0.0000	1.0000
good_11957580_-15_3AA-	1.0000	0.0000
poor_79463940_-30_2BB	0.0000	1.0000
good_19141554_-15_3AA-	1.0000	0.0000
poor_80296580_15_3BB-	0.0000	1.0000
good_11055829_30_3AB	0.8840	0.1160
good_17321648_15_3AB	1.0000	0.0000
good_14486014_15_3AB	0.6721	0.3279
poor_78970539_-30_2BB	0.0000	1.0000
good_14331589_45_3A-A-	1.0000	0.0000
good_12024489_-15_3AA-	1.0000	0.0000
poor_80058256_-15_1BB	0.0000	1.0000
poor_78810664_30_1B-B-	0.0000	1.0000
good_11486813_15_3AA-	1.0000	0.0000
poor_97314024_-45_3B-B	0.0007	0.9993
good_14411543_45_3AA-	1.0000	0.0000
good_14005274_15_3AB	1.0000	0.0000
poor_79038991_-30_1BB-	0.0000	1.0000
poor_79085047_-45_1BB	0.0000	1.0000
good_14661384_15_3A-A-	1.0000	0.0000
poor_81033173_-15_1-2BB	0.0000	1.0000
poor_82168233_15_1-2B-B	0.0000	1.0000
poor_78548643_-15_1BB	0.0000	1.0000
poor_79463940_15_2BB	0.0000	1.0000
good_19942087_45_3A-A-	1.0000	0.0000
good_19890072_-15_3AB	1.0000	0.0000
poor_77655020_30_2BB-	0.0000	1.0000
good_19527698_-30_3A-A	1.0000	0.0000
poor_77320590_30_3BB-	0.0000	1.0000
poor_79219063_-30_3B-B	0.0013	0.9987
poor_78341980_45_3B-B	0.0000	1.0000
poor_93031274_15_3B-B	0.1654	0.8346
poor_79441878_45_1B-B-	0.0000	1.0000

good_14851789_0_3AA	1.0000	0.0000
poor_78164186_45_3BB-	0.0004	0.9996
good_16286039_15_3A-A-	0.9988	0.0012
good_19942087_-15_3A-A-	1.0000	0.0000
poor_78651678_0_1B-B-	0.0000	1.0000
good_11055829_-30_3AB	1.0000	0.0000
poor_77666018_45_1B-B-	0.0000	1.0000
good_11486813_-45_3AA-	1.0000	0.0000
good_14994218_0_3A-A-	1.0000	0.0000
poor_85923677_-30_3B-B	0.0020	0.9980
good_20123884_-45_3AA-	1.0000	0.0000
good_14952697_-15_3AA-	0.9985	0.0015
good_19334426_15_3A-A-	1.0000	0.0000
poor_83001010_0_3BB-	0.0027	0.9973
poor_94202441_15_3B-B	0.0001	0.9999
poor_79867694_0_1BB	0.0000	1.0000
good_18570412_-45_3AB	1.0000	0.0000
good_16482386_0_3AA	1.0000	0.0000
poor_79867694_-45_1BB	0.0000	1.0000
poor_81581513_-15_3BB-	0.0023	0.9977
poor_78334440_30_2BB	0.0000	1.0000
poor_77272153_-15_2BB-	0.0000	1.0000
good_13318962_-45_3AB	1.0000	0.0000
poor_77509010_45_2BB-	0.0000	1.0000
good_18213488_15_3AB	1.0000	0.0000
good_12483038_-45_3A-A-	1.0000	0.0000
good_19536372_45_3AB	0.5032	0.4968
good_20205181_45_3A-A-	1.0000	0.0000
good_17300193_-15_3A-A-	1.0000	0.0000
good_15472679_30_3AA-	1.0000	0.0000
poor_77852541_30_2BB-	0.0000	1.0000
good_17878975_30_3AB	1.0000	0.0000
poor_98690041_45_3B-B	0.0000	1.0000
poor_82745365_45_3BB-	0.0000	1.0000
poor_85900848_-45_3B-B	0.0000	1.0000
poor_77655020_-30_2BB-	0.0002	0.9998
poor_80428295_0_3BB-	0.0000	1.0000
good_18935832_-45_3A-A-	1.0000	0.0000
good_15453155_-15_3AB	1.0000	0.0000
poor_81526383_-15_3BB-	0.0000	1.0000
good_13991233_15_3AA-	1.0000	0.0000
good_17392842_-30_3A-A-	0.9905	0.0095
good_11302954_30_3AA-	0.0000	1.0000
poor_98690041_-30_3B-B	0.0000	1.0000
poor_78814052_-15_2BB	0.0000	1.0000

good_11132559_-30_3A-A-	1.0000	0.0000
poor_77852541_-30_2BB-	0.0000	1.0000
poor_94994695_-15_3B-B	0.0013	0.9987
poor_83001010_45_3BB-	0.0447	0.9553
poor_78869483_-45_3B-B	0.7521	0.2479
poor_83663432_30_1-2BB-	0.0000	1.0000
good_17340290_-30_3A-A-	1.0000	0.0000
poor_83626811_30_3B-B	0.0000	1.0000
good_12731906_15_3A-A-	1.0000	0.0000
poor_79365441_-15_2BB-	0.0000	1.0000
good_15697722_15_3AA-	1.0000	0.0000
poor_94062269_30_3B-B	0.0000	1.0000
poor_77769680_30_2B-B-	0.0032	0.9968
poor_80310340_-45_1B-B-	0.0000	1.0000
poor_81105911_0_1B-B	0.0000	1.0000
poor_88330925_-45_3BB-	0.0000	1.0000
poor_78828717_-45_2B-B	0.0000	1.0000
good_13852248_30_3AB	1.0000	0.0000
poor_78634338_-45_2BB	0.0002	0.9998
good_19238542_-45_3A-A-	1.0000	0.0000
good_12483038_15_3A-A-	0.9993	0.0007
good_14952697_30_3AA-	1.0000	0.0000
poor_84080274_-15_1-2B-B	0.0000	1.0000
good_17017003_-30_3A-A-	0.9955	0.0045
poor_79431035_15_1B-B	0.0000	1.0000
poor_84092585_45_1BB-	0.0000	1.0000
good_19678783_-45_3AA-	1.0000	0.0000
poor_77206651_-15_2B-B-	0.0000	1.0000
poor_78814052_0_2BB	0.0000	1.0000
poor_77769680_0_2B-B-	0.0000	1.0000
good_13117449_30_3A-A-	1.0000	0.0000
good_15430377_-15_3A-A-	1.0000	0.0000
good_18781105_-30_3AA	1.0000	0.0000
good_11124729_30_3AA	1.0000	0.0000
good_15472679_0_3AA-	1.0000	0.0000
good_18657357_-30_3AA-	0.9739	0.0261
good_15075835_30_3A-A-	1.0000	0.0000
good_19079428_45_3A-A-	1.0000	0.0000
poor_80876604_15_1-2B-B	0.0000	1.0000
good_18213488_0_3AB	1.0000	0.0000
good_17990992_-30_3A-A-	1.0000	0.0000
poor_84106579_15_1B-B-	0.0000	1.0000
poor_79085047_0_1BB	0.0000	1.0000
good_16416586_15_3AB	1.0000	0.0000
good_14486014_-15_3AB	0.9987	0.0013

good_19079428_-30_3A-A-	1.0000	0.0000
good_17591250_30_3AA	1.0000	0.0000
poor_93633156_-15_3B-B	0.0000	1.0000
good_13858706_-15_3AA-	1.0000	0.0000
good_11311361_45_3A-A-	1.0000	0.0000
poor_78869483_15_3B-B	0.0003	0.9997
good_14450819_-30_3A-A-	1.0000	0.0000
poor_94994695_30_3B-B	0.0000	1.0000
poor_78909238_-30_3BB-	0.0000	1.0000
good_16482386_15_3AA	1.0000	0.0000
poor_82313942_30_1-2B-B-	0.0000	1.0000
good_19942087_0_3A-A-	1.0000	0.0000
good_17017003_15_3A-A-	1.0000	0.0000
good_18707760_45_3AA	0.7152	0.2848
poor_77681626_-15_1B-B	0.0000	1.0000
poor_79146459_-15_2B-B-	0.0000	1.0000
good_10423352_30_3AA-	1.0000	0.0000
poor_79478757_-45_2BB	0.0000	1.0000
good_19527698_30_3A-A	1.0000	0.0000
good_16837019_45_3A-A-	1.0000	0.0000
good_19070722_-45_3AA-	1.0000	0.0000
poor_84092585_-45_1BB-	0.0000	1.0000
good_17585937_45_3AA-	1.0000	0.0000
good_15481025_-30_3A-A	1.0000	0.0000
poor_80384973_-45_3BB-	0.0055	0.9945
good_14661384_-30_3A-A-	1.0000	0.0000
poor_96225317_15_3B-B	0.0000	1.0000
poor_83838129_-30_3BB-	0.0000	1.0000
poor_77189946_30_1BB	0.0000	1.0000
poor_80042420_0_3BB-	0.0000	1.0000
good_11838370_-45_3AB	0.9944	0.0056
poor_84565094_15_3B-B	0.0000	1.0000
good_17591250_-45_3AA	1.0000	0.0000
good_14046542_-30_3A-A-	1.0000	0.0000
good_10101873_30_3AA-	1.0000	0.0000
poor_80384973_15_3BB-	0.0023	0.9977
good_10046346_30_3AA	1.0000	0.0000
poor_83663432_-15_1-2BB-	0.0000	1.0000
poor_78265703_45_2BB-	0.0000	1.0000
good_16482386_-30_3AA	1.0000	0.0000
good_16004053_-45_3A-A-	1.0000	0.0000

Patients	STORK-Quali	Embryologist	Patient-Age	Outcome
63685691	poor	3B-B	30 or younger	Negative
34456455	poor	2BB-	33 to 34	Negative
72708419	good	3BB	33 to 34	Negative
54721684	good	3BB	30 or younger	Positive
14131815	poor	2BB-	30 or younger	Positive
67877402	poor	2B-B-	31 to 32	Negative
62270180	good	3BB	39 to 40	Negative
17243159	poor	2BB-	33 to 34	Positive
66008899	poor	3B-B	33 to 34	Negative
28271606	poor	3B-B-	33 to 34	Positive
81537530	good	3A-A-	37 to 38	Positive
15027201	good	3A-B	37 to 38	Positive
88142804	poor	2BB-	31 to 32	Positive
43274488	good	3AA	33 to 34	Positive
98859208	good	3A-B	33 to 34	Positive
25944735	poor	2BB-	33 to 34	Positive
40280930	poor	1B-B	33 to 34	Negative
81775155	good	3A-B	33 to 34	Negative
68448802	poor	2BB	33 to 34	Negative
45825345	good	3BB	33 to 34	Negative
20797011	poor	2BB	31 to 32	Positive
57702120	good	3BB	31 to 32	Positive
86013550	good	3A-B	30 or younger	Positive
64823264	poor	1BB-	41 or older	Negative
98337985	good	3A-B	35 to 36	Negative
53464007	poor	3B-B-	39 to 40	Positive
70237151	good	3AB	33 to 34	Positive
25990611	poor	3B-B	31 to 32	Negative
55716475	good	3AB	31 to 32	Positive
84922971	good	3AB	31 to 32	Positive
78251375	poor	1B-B	31 to 32	Negative
26376386	poor	1B-B-	30 or younger	Positive
45779561	good	3BB	35 to 36	Positive
61395610	good	3BB	33 to 34	Negative
63241334	poor	2.5BB-	33 to 34	Negative
65669969	good	3BB	31 to 32	Negative
30325478	good	3AB	33 to 34	Positive
91497640	good	3A-A-	31 to 32	Positive
47569705	good	3BB	33 to 34	Positive
77268846	good	3A-A-	30 or younger	Negative
26029446	poor	2BB	31 to 32	Positive

48790667	good	3A-A-	30 or younger	Positive
11973368	poor	2.5B-B	30 or younger	Positive
49179982	good	3BB	30 or younger	Positive
31783492	poor	3B-B-	33 to 34	Positive
15108343	poor	3BB-	33 to 34	Negative
76604170	poor	2BB	30 or younger	Positive
17296884	good	3BB	30 or younger	Positive
75966226	good	3BB	30 or younger	Positive
16785628	poor	1BB	37 to 38	Negative
78134875	poor	2BB	41 or older	Negative
86035421	poor	1.5BB-	33 to 34	Positive
21350119	poor	1B-B	35 to 36	Negative
23919161	poor	3BB-	30 or younger	Positive
60050063	poor	2B-B	37 to 38	Negative
94290954	poor	1B-B-	37 to 38	Negative
81775263	good	3BB	35 to 36	Negative
60596156	good	3BB	35 to 36	Positive
83678771	poor	3B-B-	35 to 36	Positive
56340302	good	3A-B	35 to 36	Negative
91915868	good	3BB	35 to 36	Positive
26651808	poor	2BB	30 or younger	Negative
55420469	poor	2BB	30 or younger	Negative
59998682	good	3BB	39 to 40	Positive
45715792	poor	3BB-	39 to 40	Positive
83852410	poor	2B-B-	39 to 40	Negative
25015508	poor	2BB	39 to 40	Negative
93685015	good	3AA-	39 to 40	Negative
23856180	good	3AB	39 to 40	Negative
84212965	good	3AB	37 to 38	Positive
42620433	good	3A-B	37 to 38	Positive
36427559	good	3AB	35 to 36	Positive
81039566	good	3BB	35 to 36	Positive
80570420	poor	2BB-	33 to 34	Positive
94111926	good	3A-B	37 to 38	Negative
74468327	poor	2B-B-	37 to 38	Positive
14466899	poor	2B-B-	31 to 32	Positive
48346027	good	3BB	31 to 32	Positive
75560497	poor	3B-B	39 to 40	Positive
35782109	poor	2BB-	39 to 40	Positive
56248002	good	3BB	39 to 40	Negative
37508905	poor	1.5BB-	39 to 40	Negative
18881661	good	3BB	41 or older	Negative

90103980	good	3AB	33 to 34	Positive
51866389	good	3BB	33 to 34	Positive
57950189	poor	3B-B	33 to 34	Positive
80444634	poor	2BB-	37 to 38	Positive
14964408	poor	1B-B-	41 or older	Negative
53222137	poor	3BB-	35 to 36	Positive
86245269	good	3A-B	35 to 36	Positive
15102907	good	3AA	31 to 32	Positive
26074510	good	3AA	31 to 32	Positive
45097572	good	3BB	35 to 36	Positive
28774476	poor	2.5BB-	33 to 34	Positive
69190533	good	3BB	33 to 34	Positive
37244773	poor	3B-B	33 to 34	Positive
89965037	poor	1B-B-	33 to 34	Positive
32662374	good	3A-B	30 or younger	Positive
97178979	poor	2.5B-B	33 to 34	Positive
39030397	good	3A-B	31 to 32	Positive
37601803	good	3AA	30 or younger	Positive
91062246	poor	1BB-	31 to 32	Positive
78555781	poor	2BB	31 to 32	Positive
60736085	good	3A-B	31 to 32	Positive
86952923	poor	2.5BB-	30 or younger	Negative
20072235	poor	1BB	30 or younger	Negative
65889587	poor	3BB-	30 or younger	Negative
62453514	good	3A-B	31 to 32	Positive
30048987	good	3A-B	30 or younger	Negative
60585570	good	3AB	35 to 36	Negative
33687990	good	3A-B	35 to 36	Negative
24454769	poor	2BB-	39 to 40	Negative
27344072	poor	2B-B	39 to 40	Negative
93431058	poor	1B-B-	35 to 36	Negative
22580463	poor	3BB-	31 to 32	Negative
29131935	good	3A-B	39 to 40	Positive
80802859	good	3AB	39 to 40	Positive
92566827	poor	2BB-	37 to 38	Negative
84183960	good	3A-B	35 to 36	Negative
86916090	good	3AA-	35 to 36	Negative
32220582	poor	3B-B-	33 to 34	Negative
91896764	poor	2B-B-	33 to 34	Negative
12770560	poor	3B-B	33 to 34	Negative
26025713	poor	1.5BB	33 to 34	Positive
92175870	poor	2.5BB-	33 to 34	Negative

80888849	poor	2.5BB-/C	33 to 34	Negative
22956423	poor	2.5B-B	33 to 34	Positive
43852154	good	3BB	33 to 34	Positive
74539200	poor	1B-B	39 to 40	Negative
91851193	poor	2BB	39 to 40	Negative
65463743	poor	1B-B-/C	39 to 40	Negative
47572064	good	3BB	33 to 34	Negative
34131799	poor	2B-B	30 or younger	Negative
93292668	poor	2B-B	30 or younger	Positive
82191075	poor	2BB-	35 to 36	Positive
17085693	poor	2BB-	35 to 36	Positive
24089012	good	3A-A-	33 to 34	Positive
37820023	good	3A-A	33 to 34	Positive
38993644	good	3A-B	39 to 40	Negative
14895606	good	3BB	39 to 40	Negative
72474967	poor	1BB-	30 or younger	Positive
12936184	good	3BB	31 to 32	Positive
20884770	poor	2B-B	37 to 38	Negative
58254887	good	3BB	31 to 32	Positive
21377193	poor	2B-B-	33 to 34	Positive
91665921	poor	3BB	30 or younger	Negative
64555311	poor	3BB	30 or younger	Negative
43772228	poor	2.5B-B-	30 or younger	Negative
57461078	poor	3B-B-	30 or younger	Negative
98017077	good	3BB	30 or younger	Positive
47447053	good	3BB	30 or younger	Positive
50590598	poor	3B-/CB	39 to 40	Positive
53632727	poor	1B-B-	39 to 40	Negative
53877914	good	3AB	37 to 38	Positive
29580602	good	3BB	37 to 38	Negative
94062269	poor	3B-B	37 to 38	Positive
48286338	good	3A-A-	30 or younger	Positive
12219496	poor	2.5BB-	31 to 32	Positive
45159575	poor	3BB	33 to 34	Negative
90808937	good	3A-A-	31 to 32	Negative
78358707	good	3AA-	31 to 32	Negative
94833778	poor	3BB-	39 to 40	Positive
28496706	poor	2B-B-/C	31 to 32	Positive
29804392	poor	3BB-	33 to 34	Negative
85816609	good	3Ab	33 to 34	Positive
33996213	good	3AB	41 or older	Positive
62361130	good	3AB	41 or older	Positive

17325858	poor	3B-B	31 to 32	Positive
87597991	poor	2B-B-	30 or younger	Positive
94539634	poor	2B-B-/C	33 to 34	Negative
70701968	poor	2B-B-	33 to 34	Negative
72499901	poor	2B-B-	35 to 36	Positive
22968625	good	3A-B	31 to 32	Positive
92498946	poor	3B-B-	37 to 38	Negative
32706681	poor	2B-B	37 to 38	Negative
93597139	good	3A-A-	31 to 32	Negative
53160502	good	3A-B	35 to 36	Negative
37740401	good	3A-B	35 to 36	Negative
19590335	good	3A-B	39 to 40	Positive
50810973	poor	3BB-	39 to 40	Negative
63136699	poor	3BB-	39 to 40	Negative
30090366	poor	3BB-	39 to 40	Negative
31727517	good	3BB	39 to 40	Negative
69490150	good	3A-A	33 to 34	Positive
25594594	good	3A-B	33 to 34	Positive
80264654	poor	2BB	33 to 34	Positive
80545800	good	3BB	33 to 34	Positive
35635677	poor	2B-B-	39 to 40	Negative
67707481	poor	3B-B	39 to 40	Negative
70035440	good	3BB	33 to 34	Positive
35896148	poor	3BB-	33 to 34	Positive
85194824	good	3A-B	35 to 36	Negative
16559088	poor	2.5B-B	33 to 34	Negative
77174924	poor	2B-B-	33 to 34	Negative
86096940	poor	2B-B	30 or younger	Positive
25291468	poor	3A-B	33 to 34	Positive
97166359	good	3A-B	33 to 34	Positive
36048939	poor	2BB	37 to 38	Negative
84888863	poor	3B-B	37 to 38	Negative
97254863	poor	2B-B-	37 to 38	Positive
92375325	poor	2BB	33 to 34	Positive
94817037	good	3BB	33 to 34	Negative
11666570	good	3.5AA	33 to 34	Positive
39418455	poor	1B-B	39 to 40	Negative
80530664	poor	1.5B-B	39 to 40	Negative
28083688	poor	2BB	30 or younger	Negative
87102044	good	3A-B	30 or younger	Negative
55166495	good	3BB	30 or younger	Negative
72933060	poor	3B-B-	33 to 34	Positive

95178981	good	3A-B	33 to 34	Negative
78194144	poor	2BB	33 to 34	Negative
60871492	good	3BA-	39 to 40	Negative
36868408	good	3A-B	39 to 40	Negative
54334366	good	3AB	39 to 40	Negative
23265700	good	3BB	30 or younger	Positive
61029387	good	3A-B	30 or younger	Positive
60869657	good	3A-A-	37 to 38	Positive
41467233	good	3A-B	37 to 38	Positive
53834357	poor	3BB-	35 to 36	Positive
49841390	poor	2.5BB-	35 to 36	Positive
30556689	poor	3BB-	35 to 36	Positive
32536565	poor	3B-B	35 to 36	Positive
82212265	poor	1.5BB	30 or younger	Negative
97597986	good	3BB	30 or younger	Positive
54249142	poor	3BB-	35 to 36	Positive
69617340	good	3BB	35 to 36	Positive
95746872	poor	2BB	35 to 36	Positive
10718644	good	3AA	35 to 36	Negative
75848157	good	3A-B	35 to 36	Negative
92849918	poor	2BB-	39 to 40	Negative
83875675	good	3AA	31 to 32	Positive
74423501	good	3BB	31 to 32	Positive
61039941	good	3BB	37 to 38	Positive
11353352	good	3A-B	37 to 38	Positive
21600374	poor	2.5BB-	31 to 32	Positive
97790489	good	3AA	31 to 32	Negative
82340556	good	3A-A	31 to 32	Negative
46307835	poor	2B-B	30 or younger	Positive
24676163	poor	3BB-	30 or younger	Positive
13768233	poor	2.5B-B-	35 to 36	Negative
26525086	poor	2B-B	35 to 36	Negative
81105911	poor	1B-B	35 to 36	Negative
51349727	poor	1.5BB-	33 to 34	Negative
80247673	poor	2BB-	33 to 34	Negative
35871960	good	3BB	31 to 32	Negative
48158541	good	3BB	31 to 32	Negative
10587245	poor	3BB-	31 to 32	Negative
41334238	poor	3BB-	31 to 32	Negative
28972236	poor	3B-B	31 to 32	Positive
34812433	good	3A-B	31 to 32	Positive
81276152	poor	3B-B-	31 to 32	Positive

40795381	good	3AA	31 to 32	Positive
50346580	good	3AA	31 to 32	Positive
25287867	poor	3BB-	41 or older	Negative
75218312	poor	3B-B-/C	41 or older	Negative
21678545	good	3BB	30 or younger	Positive
96738489	good	3A-A-	30 or younger	Positive
91529626	poor	2.5B-B	35 to 36	Negative
72314471	good	3AA	30 or younger	Positive
32119925	good	3AA	30 or younger	Positive
96527048	good	3BB	33 to 34	Positive
83558125	good	3A-B	33 to 34	Positive
85812022	poor	2BB	33 to 34	Positive
86066301	good	3A-B	41 or older	Positive
14046542	good	3A-A-	41 or older	Positive
14254300	good	3BB	41 or older	Negative
11038955	good	3A-A-	30 or younger	Positive
25153430	poor	2B-B-	30 or younger	Negative
53366134	poor	2B-B-	35 to 36	Positive
61704844	good	3A-A-	31 to 32	Positive
42111191	poor	1B-B	30 or younger	Positive
50362332	poor	1B-B-	41 or older	Negative
62436614	good	3AA	37 to 38	Negative
76182673	good	3AA	37 to 38	Negative
95084418	poor	1B-B-	31 to 32	Positive
56105575	poor	1B-B-	31 to 32	Positive
52280505	good	3A-B	30 or younger	Negative
80204552	good	3AB	30 or younger	Positive
78719513	poor	3BB-	31 to 32	Positive
37229167	poor	3B-B	31 to 32	Positive
82610654	poor	2.5BB-	41 or older	Negative
77666018	poor	1B-B-	41 or older	Negative
96007542	good	3A-B	41 or older	Positive
20014226	poor	2.5BB-	41 or older	Positive
41159897	poor	1.5B-B	30 or younger	Positive
13396280	poor	3BB-	33 to 34	Negative
13001139	poor	2BB-	39 to 40	Negative
12828151	good	3AB	35 to 36	Positive
64880983	good	3BB	35 to 36	Positive
50004027	good	3BB	39 to 40	Negative
88985528	poor	2B-B-	39 to 40	Negative
26693143	poor	2B-B	30 or younger	Negative
58288576	good	3A-B	30 or younger	Negative

18789120	poor	2B-B	30 or younger	Negative
70948156	good	3A-B	30 or younger	Positive
50140207	good	3A-A-	30 or younger	Positive
59347377	good	3A-A-	35 to 36	Positive
52691144	poor	2BB-	37 to 38	Negative
20766484	poor	2BB-	37 to 38	Negative
88266792	poor	3BB-	33 to 34	Positive
15472679	good	3AA-	33 to 34	Negative
22453579	poor	1B-B-	33 to 34	Negative
53401850	poor	3B-B	33 to 34	Positive
24690276	poor	2.5B-B	31 to 32	Negative
17508008	good	3BB	33 to 34	Positive
62322629	good	3BB	33 to 34	Positive
84170294	good	3AB	33 to 34	Positive
44396910	good	4AA	31 to 32	Positive
40928464	poor	3B-B-	39 to 40	Negative
49185060	poor	3B-B-	39 to 40	Negative
52496614	poor	3B-B	35 to 36	Positive
77788406	poor	2B-B	35 to 36	Positive
30506261	poor	1B-B-	41 or older	Negative
41149905	poor	2BB-	41 or older	Negative
71591060	good	3BB	30 or younger	Positive
96893279	good	3BB	30 or younger	Positive
42453481	poor	2BB-	33 to 34	Positive
51795422	good	3AB	35 to 36	Positive
17300193	good	3A-A-	37 to 38	Negative
42220863	good	3BB	33 to 34	Positive
12104082	poor	3BB-	39 to 40	Negative
23733194	poor	3B-B-	39 to 40	Negative
86952498	good	3AB	35 to 36	Positive
11165082	good	3A-B	39 to 40	Negative
75103887	good	3A-B	39 to 40	Negative
87353387	poor	3BB	39 to 40	Negative
85246378	good	3AA	39 to 40	Negative
93342217	poor	3B-B-	35 to 36	Negative
48226830	good	3AA	35 to 36	Negative
58281157	poor	1BB	31 to 32	Negative
30168481	poor	1BB	31 to 32	Negative
71470896	poor	2B-B-	35 to 36	Negative
11835134	poor	1B-B-	35 to 36	Positive
25172379	poor	3B-B	35 to 36	Negative
97960922	poor	3B-B-	41 or older	Positive

37178160	good	3BB	39 to 40	Positive
94451686	poor	1BB	39 to 40	Positive
74052366	good	3BB	35 to 36	Positive
74527968	good	3A-A-	33 to 34	Positive
56262216	good	3AB	35 to 36	Negative
22845691	poor	3BB-	33 to 34	Negative
97354707	poor	1.5B-B-	31 to 32	Negative
13724519	good	3BB	33 to 34	Positive
85335760	poor	3BB-	33 to 34	Positive
13533354	poor	2BB-	33 to 34	Positive
16311464	good	3BB	33 to 34	Positive
15788276	poor	3B-B	39 to 40	Positive
24191777	poor	1B-B-	39 to 40	Positive
18747562	poor	2.5BB-	39 to 40	Negative
56615735	poor	1BB	39 to 40	Negative
96637388	good	3BB	35 to 36	Positive
67613161	good	3BB	41 or older	Positive
84610766	poor	2B-B	41 or older	Negative
59320662	good	3BB	33 to 34	Positive
53712257	poor	3BB-	33 to 34	Positive
23551468	good	3BB	33 to 34	Positive
17383268	poor	2.5BB-	35 to 36	Positive
54055751	poor	3BB	35 to 36	Positive
14673951	poor	3B-B	35 to 36	Positive
18590470	good	3A-B	35 to 36	Positive
21496736	good	3AA-	35 to 36	Positive
93880048	poor	2BB	35 to 36	Negative
94567608	poor	1B-B-	35 to 36	Negative
13061921	poor	1B-B-	35 to 36	Negative
10777011	good	3A-B	35 to 36	Negative
32994173	good	3A-A-	35 to 36	Negative
13833375	good	3BB	35 to 36	Negative
41165375	good	3AA	33 to 34	Positive
92665799	good	3A-B	33 to 34	Negative
66241793	good	3A-B	33 to 34	Positive
62099367	poor	2BB-	39 to 40	Negative
22401645	good	3BB	35 to 36	Positive
69594060	poor	1BB-	37 to 38	Negative
45578419	poor	2B-B-	37 to 38	Positive
96074766	good	3BB	30 or younger	Positive
75426688	poor	1.5BB	30 or younger	Positive
18657357	good	3AA-	30 or younger	Negative

37735452	good	3A-B	30 or younger	Positive
81292067	poor	2.5B-B-	37 to 38	Negative
35595473	good	3BB	33 to 34	Positive
84721062	poor	2BB-	39 to 40	Positive
63877083	good	3AA	39 to 40	Negative
86029689	poor	2BB	30 or younger	Positive
54901847	poor	1.5BB-	31 to 32	Negative
94698381	poor	1BB-	31 to 32	Negative
12339198	good	3BB	31 to 32	Negative
20253695	good	3AB	33 to 34	Negative
15742703	poor	2B-B	33 to 34	Negative
17117143	poor	2BB-	33 to 34	Negative
68192738	poor	3BB-/C	33 to 34	Negative
40070555	good	3AA	33 to 34	Positive
13944933	poor	3BB-	33 to 34	Negative
96156937	good	3BB	33 to 34	Negative
39721131	good	3AA	33 to 34	Positive
68136819	good	3AA	33 to 34	Positive
99807162	poor	2.5BB-	37 to 38	Positive
24546615	poor	3B-B	33 to 34	Negative
69056051	poor	2BB-	33 to 34	Positive
67392971	poor	2BB	33 to 34	Negative
98771185	poor	2.5B-B-/C	35 to 36	Positive
73038734	poor	3B-B-	30 or younger	Positive
29980599	good	3BB	30 or younger	Positive
20313723	poor	3B-B-	30 or younger	Positive
78345868	good	3BB	30 or younger	Positive
81370326	good	3AA	30 or younger	Positive
35840735	poor	2B-B-	30 or younger	Negative
28795560	good	3AB	30 or younger	Negative
15222909	good	3A-B	30 or younger	Negative
88851922	good	3A-A	30 or younger	Positive
61939605	poor	3BB	30 or younger	Positive
22295244	good	3AB	33 to 34	Positive
77803033	good	3AB	33 to 34	Positive
98844646	good	3A-B	39 to 40	Negative
83388442	good	3BB	39 to 40	Negative
68964716	good	3BB	39 to 40	Negative
91932561	good	3BB	39 to 40	Negative
57762937	poor	2BB-	31 to 32	Negative
66098481	good	3BB	31 to 32	Positive
63423070	good	3AB	33 to 34	Negative

56489930	good	3A-B	31 to 32	Positive
92888433	good	3A-B	31 to 32	Positive
36292194	poor	1B-B-	31 to 32	Negative
23015143	poor	2.5B-B	31 to 32	Negative
38819794	poor	2BB	39 to 40	Negative
22792535	poor	2BB	39 to 40	Negative
35912019	poor	3B-B-/C	33 to 34	Positive
18806973	poor	2BB	37 to 38	Negative
28859350	poor	2B-B-	37 to 38	Negative
19536372	good	3AB	31 to 32	Positive
57065645	good	3AA	31 to 32	Positive
97645729	good	3AA-	30 or younger	Positive
57924419	poor	3BB-	31 to 32	Positive
37879908	poor	2BB	31 to 32	Negative
67073469	poor	1B-B-	37 to 38	Negative
54239290	good	3AA	35 to 36	Positive
39198242	good	3BB	30 or younger	Positive
27297179	good	3A-B	30 or younger	Positive
53810673	good	3AB	35 to 36	Positive
43613630	good	3BB	35 to 36	Positive
94560342	good	3AB	35 to 36	Positive
22292826	good	3AA	35 to 36	Positive
39008723	good	3AA	35 to 36	Positive
36913793	poor	3B-B-	41 or older	Positive
32157418	poor	3BB-	37 to 38	Negative
52642470	good	3AB	37 to 38	Negative
69544088	good	3A-B	33 to 34	Positive
30555538	good	3A-A-	33 to 34	Negative
42575442	good	3A-B	31 to 32	Positive
95292145	good	3A-B	31 to 32	Positive
80692621	poor	2B-B-	35 to 36	Negative
65191013	good	3BA-	35 to 36	Negative
72441806	good	3A-B	35 to 36	Negative
56081361	poor	2BB	35 to 36	Positive
69584182	poor	1BB	35 to 36	Positive
50006565	good	3BB	33 to 34	Positive
78981490	good	3A-B	37 to 38	Negative
78454606	poor	2.5B-B-	39 to 40	Negative
83001565	poor	2.5B-B-	39 to 40	Negative
13256762	good	3BB	30 or younger	Negative
35188238	poor	3A-B	30 or younger	Negative
10604335	poor	2.5B-B	39 to 40	Negative

30521481	poor	1BB	31 to 32	Positive
57713518	poor	3BB-	37 to 38	Negative
81481038	poor	3B-B-	37 to 38	Negative
55791994	poor	3BB-	37 to 38	Negative
81581513	poor	3BB-	33 to 34	Negative
43675539	good	3A-A-	33 to 34	Positive
12773594	poor	3BB-	33 to 34	Positive
68154216	poor	1.5BB	35 to 36	Positive
24225165	good	3BB	30 or younger	Negative
99099293	good	3A-A-	30 or younger	Negative
33133845	good	3A-B	30 or younger	Positive
79521449	good	3A-A-	33 to 34	Negative
44740828	good	3A-B	33 to 34	Positive
44685681	poor	3BA-	33 to 34	Positive
98274894	good	3AA	33 to 34	Negative
41267673	good	3BB	35 to 36	Negative
24371676	good	3BB	35 to 36	Negative
85024461	poor	2B-B	39 to 40	Positive
61615880	poor	2B-B	33 to 34	Positive
83549474	poor	2BB-	37 to 38	Negative
43818964	poor	1BB	39 to 40	Positive
28712098	poor	3BB	39 to 40	Negative
89372461	good	3AB	35 to 36	Negative
71739140	poor	1B-B-	39 to 40	Negative
74622113	poor	1BB-	39 to 40	Negative
55792508	good	3BB	31 to 32	Positive
57111741	good	3BB	31 to 32	Positive
11553115	poor	2B-B-	35 to 36	Negative
11580128	good	3A-B	30 or younger	Negative
81776568	good	3A-A-	30 or younger	Negative
53919629	good	3BB	30 or younger	Negative
41178194	good	3AA	30 or younger	Positive
92390266	good	3AA	31 to 32	Negative
76404583	poor	3B-B	31 to 32	Positive
84183923	good	3AA	30 or younger	Positive
52184281	poor	3BB	35 to 36	Positive
94797319	good	3AB	35 to 36	Positive
74374673	poor	3BB-	33 to 34	Positive
49046233	good	3AA	33 to 34	Positive
83816694	poor	2BB	39 to 40	Positive
95319069	good	3AB	37 to 38	Positive
26954915	good	3A-B	35 to 36	Positive

92270015	good	3AA-	35 to 36	Positive
39918515	good	3BB	30 or younger	Positive
82097420	good	3A-B	30 or younger	Positive
52913210	poor	1BB	33 to 34	Positive
63551572	poor	2BB	33 to 34	Positive
94151167	good	3A-A-	31 to 32	Positive
45211072	poor	2.5B-B-	39 to 40	Negative
24499203	poor	2BB	37 to 38	Negative
81057182	good	3A-B	31 to 32	Negative
42541997	good	3AA	31 to 32	Positive
11273163	poor	3BB-	31 to 32	Negative
92568209	poor	3B-B	31 to 32	Positive
26598358	poor	3B-B-	30 or younger	Positive
86408783	poor	2.5B-B	30 or younger	Positive
57876732	good	3AB	30 or younger	Positive
19527698	good	3A-A	30 or younger	Positive
68521796	good	3AA	30 or younger	Negative
50297641	poor	2B-B	33 to 34	Positive
71185823	poor	3B-B	33 to 34	Positive
60890076	poor	2B-B-	30 or younger	Positive
69981287	poor	1BB	33 to 34	Positive
93150996	poor	2BB	39 to 40	Negative
10617532	poor	2BB	39 to 40	Negative
39934437	poor	3A-B	33 to 34	Positive
21699615	good	3AB	33 to 34	Positive
17794119	good	3A-B	30 or younger	Positive
31307131	poor	2BB-	30 or younger	Positive
39924058	poor	2B-B	39 to 40	Negative
48064661	good	3A-B	37 to 38	Positive
11081289	good	3A-B	35 to 36	Positive
47367423	good	3BA-	35 to 36	Positive
31051082	poor	3B-B	39 to 40	Positive
80404108	poor	3B-B	39 to 40	Positive
32045391	good	3BB	35 to 36	Negative
47542070	good	3BB	33 to 34	Positive
75787058	poor	2B-B-	30 or younger	Negative
66043529	good	3BB	33 to 34	Positive
50882977	poor	2BB-	31 to 32	Positive
25264941	poor	1B-B-	39 to 40	Negative
87430735	good	3BB	39 to 40	Negative
89817112	good	3BB	39 to 40	Negative
34628528	good	3AB	30 or younger	Positive

60733572	good	3A-A-	30 or younger	Positive
42406425	good	3BA-	31 to 32	Negative
31879857	poor	3B-B-	31 to 32	Positive
88712016	good	3BB	35 to 36	Negative
23890414	poor	2BB-	35 to 36	Negative
41166791	good	3A-A-	30 or younger	Positive
61302434	good	3AA	30 or younger	Positive
27975325	good	3BB	30 or younger	Positive
47251649	good	3A-A	30 or younger	Positive
81855979	poor	1B-B-/C	39 to 40	Negative
16120958	poor	1B-B-/C	39 to 40	Negative
99115670	good	3AA	30 or younger	Positive
61473004	poor	3A-B	33 to 34	Negative
45459192	poor	1.5B-B	33 to 34	Negative
25885058	poor	2BB-	33 to 34	Negative
61470662	poor	2BB-	31 to 32	Positive
32652953	good	3AA	30 or younger	Positive
25512940	good	3AA-	30 or younger	Positive
59155342	good	3A-A-	30 or younger	Positive
78715791	poor	3BB-	33 to 34	Positive
63958877	good	3BA-	33 to 34	Negative
46186221	good	3A-B	37 to 38	Negative
96189138	good	3A-B	37 to 38	Negative
88160824	poor	2BB	35 to 36	Negative
75218140	poor	2BB	35 to 36	Negative
51306474	poor	2BB-	37 to 38	Negative
79350865	poor	2B-B-	37 to 38	Positive
32366564	poor	2BB	37 to 38	Negative
19342799	poor	1BB	37 to 38	Positive
84429086	good	3BB	37 to 38	Negative
92323593	good	3AA	35 to 36	Positive
10745533	poor	3BB	35 to 36	Positive
90089512	good	3A-B	35 to 36	Positive
78531629	good	3A-A-	41 or older	Positive
55727640	poor	3BB-	41 or older	Negative
53916269	poor	1B-B-/C	41 or older	Negative
99176374	good	3A-B	30 or younger	Positive
52974471	good	3BB	30 or younger	Positive
92514609	poor	2BB	31 to 32	Positive
99040894	poor	2.5BB-	37 to 38	Negative
98382029	poor	2BB	35 to 36	Positive
68371928	poor	3B-B-	35 to 36	Positive

36506599	good	3A-A	37 to 38	Positive
13158796	good	3A-A	31 to 32	Negative
72939211	poor	3BB	31 to 32	Positive
85503073	poor	2B-B	37 to 38	Positive
21832602	good	3A-A-	37 to 38	Positive
56254337	good	3A-A-	35 to 36	Negative
26584532	good	3A-A	35 to 36	Positive
50195460	good	3AB	35 to 36	Negative
75188351	good	3BB	35 to 36	Positive
20288250	good	3A-B	31 to 32	Positive
62494772	good	3A-A-	31 to 32	Positive
25802062	good	3AB	35 to 36	Positive
50056959	good	3AA	35 to 36	Negative
18102857	good	3AA-	35 to 36	Positive
97719613	good	3AA	35 to 36	Negative
87407498	good	3BB	30 or younger	Positive
43769568	good	3A-A	30 or younger	Positive
97980094	good	3A-B	30 or younger	Negative
37880744	good	3AA-	30 or younger	Positive
22288342	good	3A-B	30 or younger	Positive
35620090	good	3A-A-	30 or younger	Positive
14112595	good	3BB	30 or younger	Positive
22043797	good	3AB	37 to 38	Negative
20581787	good	3AA	37 to 38	Negative
34146847	good	3A-A-	37 to 38	Negative
48557448	poor	3B-B	37 to 38	Negative
92175648	good	3AA	30 or younger	Positive
80329242	good	3AA	30 or younger	Positive
37456460	good	3AA	31 to 32	Positive
21476908	good	3BB	30 or younger	Positive
96671587	good	3BB	37 to 38	Negative
70210306	good	3A-B	37 to 38	Negative
49927088	good	3BB	35 to 36	Positive
14278503	poor	3BB-/C	35 to 36	Positive
94993271	good	3AA	31 to 32	Positive
61561285	good	3AA-	31 to 32	Positive
35002048	poor	3BB-	41 or older	Negative
10069171	good	3BB	30 or younger	Negative
50758837	good	3A-A	30 or younger	Negative
48932213	poor	3B-B-	37 to 38	Negative
37132582	good	3BB	37 to 38	Negative
78207319	good	3A-A-	31 to 32	Positive

18765488	good	3A-B	35 to 36	Positive
59682329	good	3BB	30 or younger	Positive
17612946	good	3A-A-	33 to 34	Negative
46303134	poor	3A-B	35 to 36	Negative
62353321	poor	3A-B	35 to 36	Positive
30335299	poor	2B-B	35 to 36	Negative
10366177	poor	2BB	35 to 36	Positive
92785313	poor	3B-B	37 to 38	Negative
30102249	good	3BA-	37 to 38	Negative
10506855	good	3A-B	37 to 38	Positive
53044301	poor	3BB	30 or younger	Negative
89461080	poor	2.5BB-	30 or younger	Negative
64030993	good	3BB	31 to 32	Negative
71819503	good	3BB	31 to 32	Negative
45386043	good	3BB	30 or younger	Positive
86906680	good	3BB	30 or younger	Positive
99515363	good	3AB	37 to 38	Positive
50862964	good	4AB	33 to 34	Positive
91936880	poor	2BB	30 or younger	Positive
49671975	poor	1B-B	30 or younger	Positive
69610034	good	3A-B	41 or older	Negative
35174457	poor	1.5B-B-	30 or younger	Positive
91250056	poor	2BB-	30 or younger	Positive
65249151	good	3A-A-	33 to 34	Negative
11559906	poor	1B-B-/C	41 or older	Negative
69753833	good	3BB	33 to 34	Negative
84116513	poor	1BB-	33 to 34	Negative
27987203	good	3BB	33 to 34	Negative
96253565	poor	2BB	35 to 36	Negative
91707565	poor	3BB-	35 to 36	Negative
70045374	good	3BB	35 to 36	Negative
25315242	poor	3BB-	35 to 36	Negative
25994060	poor	3BB	35 to 36	Negative
77236795	poor	3BB-	41 or older	Negative
65044674	poor	3BB-	33 to 34	Positive
56696594	poor	3B-B	33 to 34	Positive
48583692	good	3A-B	31 to 32	Positive
82885313	poor	3BB-	41 or older	Negative
96598365	good	3BB	41 or older	Negative
19179528	poor	3BB-	35 to 36	Negative
28197679	poor	2BB	37 to 38	Positive
56116129	poor	3BB-	35 to 36	Positive

70588794	good	3A-A	35 to 36	Positive
11183795	good	3AB	35 to 36	Positive
81322712	poor	1.5B-B	35 to 36	Positive
84796817	poor	1BB	41 or older	Negative
62344663	good	3AA-	41 or older	Negative
11895674	poor	1BB	31 to 32	Positive
96934468	poor	1BB	31 to 32	Positive
23765483	good	3AA	33 to 34	Positive
25127570	good	3AA-	33 to 34	Positive
97211513	poor	2B-B	30 or younger	Negative
27058489	good	3BA-	30 or younger	Negative
13729605	poor	2B-B-	41 or older	Negative
86172597	good	3AA	30 or younger	Positive
54195668	good	3AA-	30 or younger	Positive
20123884	good	3AA-	39 to 40	Positive
83081873	good	3A-A-	33 to 34	Positive
27002081	poor	1BB	41 or older	Negative
37781185	poor	1.5BB	41 or older	Negative
63210844	poor	2BB	41 or older	Negative
10724862	poor	2BB	37 to 38	Positive
72192681	poor	1.5B-B	30 or younger	Positive
38845777	good	3A-A-	33 to 34	Positive
54802774	good	3BB	33 to 34	Positive
39652286	poor	1.5BB	39 to 40	Negative
35915522	good	3AA	35 to 36	Positive
16482386	good	3AA	35 to 36	Positive
86503538	good	3A-A-	31 to 32	Positive
63600134	good	3BB	31 to 32	Positive
73466161	good	3BB	31 to 32	Negative
77675171	good	3AA-	35 to 36	Positive
19850991	good	3BB	37 to 38	Positive
90001306	good	3AA	37 to 38	Positive
66800883	poor	1.5BB	37 to 38	Negative
16737103	poor	1.5BB	37 to 38	Positive
35619864	good	3AA	31 to 32	Positive
26390497	poor	3B-B-	37 to 38	Negative
35751488	poor	2BB-	37 to 38	Negative
72032487	good	3BB	33 to 34	Negative
25312178	poor	3BB-	33 to 34	Positive
59132799	good	3AB	31 to 32	Positive
66503199	good	3AA	31 to 32	Positive
12368448	poor	2.5BB-	37 to 38	Negative

81678844	poor	3BB-	37 to 38	Negative
27122908	good	5A-A-	30 or younger	Positive
34997423	good	3A-B	31 to 32	Negative
45516319	good	3AB	30 or younger	Positive
56757450	poor	1.5BB	30 or younger	Positive
62809704	poor	2BB	30 or younger	Positive
55772427	poor	2BB	30 or younger	Negative
65965653	good	3A-B	35 to 36	Positive
28604175	good	3A-A-	35 to 36	Positive
15236547	poor	2.5B-B	37 to 38	Negative
48082909	good	3AA-	41 or older	Positive
41342951	poor	3BB-	41 or older	Positive
43817066	good	3A-A	33 to 34	Positive
67089893	good	3AB	30 or younger	Positive
43969091	good	3AA	37 to 38	Positive
77650194	poor	2B-B-	37 to 38	Negative
37193419	poor	3B-B-	37 to 38	Negative
23404190	poor	2B-B-	35 to 36	Positive
95219612	good	3A-B	37 to 38	Positive
94228067	good	3BB	37 to 38	Positive
89578446	poor	1.5BB	39 to 40	Positive
86930102	good	3BB	30 or younger	Positive
91899505	good	3A-B	37 to 38	Positive
15374462	good	3BB	33 to 34	Positive
79672209	good	3AA-	37 to 38	Positive
66230511	good	3AB	33 to 34	Negative
21786500	good	3AA	33 to 34	Negative
11842286	poor	3B-B-	31 to 32	Positive
87749651	poor	3B-B	31 to 32	Positive
21763943	poor	2BB	31 to 32	Positive
74012931	good	3AA-	41 or older	Positive
16266445	poor	3BB-	41 or older	Negative
13830301	poor	2B-B	41 or older	Negative
34267612	poor	1B-B-	31 to 32	Positive
28983733	poor	2BB	31 to 32	Positive
23871837	poor	3BB-	41 or older	Negative
90334454	poor	3BB-	41 or older	Negative
58533394	good	3A-B	30 or younger	Negative
18213488	good	3AB	30 or younger	Negative
52816209	poor	1.5BB-	33 to 34	Negative
27316188	poor	2BB	33 to 34	Negative
17003193	good	3AA	35 to 36	Negative

51782536	good	3AB	35 to 36	Positive
52104703	good	3AA-	35 to 36	Positive
57428965	good	3A-B	33 to 34	Positive
95604000	good	3AA	37 to 38	Positive
37787687	good	3AA	37 to 38	Positive
17257791	good	3AA	37 to 38	Negative
49056217	good	3BB	33 to 34	Negative
22249546	good	3A-B	31 to 32	Positive
53185788	good	3BB	31 to 32	Positive
69506877	good	3BB	39 to 40	Negative
14851789	good	3AA	33 to 34	Positive
77629731	good	3A-B	37 to 38	Negative
20732032	good	3AA	35 to 36	Negative
53475907	good	3A-A-	30 or younger	Positive
25230000	good	3A-B	33 to 34	Positive
25911973	good	3AA	33 to 34	Positive
15336100	good	3BA-	33 to 34	Positive
56462174	poor	2B-B-	41 or older	Negative
16801536	poor	3BB-	41 or older	Negative
77042598	poor	1B-B-	37 to 38	Negative
43851275	poor	2BB-	37 to 38	Negative
92290329	good	3A-B	39 to 40	Positive
13026323	poor	3BB-	39 to 40	Positive
50463664	poor	2BB	37 to 38	Negative
61993440	good	3BB	31 to 32	Positive
58571144	poor	2.5BC	39 to 40	Positive
92717744	good	3BB	33 to 34	Negative
39428598	poor	3BB-	33 to 34	Negative
16364902	good	3A-B	33 to 34	Positive
23901575	poor	2B-B-/C	41 or older	Negative
26907987	poor	3BB	33 to 34	Positive
51938424	good	3BB	33 to 34	Positive
46067207	poor	3BB-	41 or older	Negative
91913160	poor	2B-B-	41 or older	Negative
85768330	poor	2BB-	41 or older	Negative
14267142	poor	2B-B-	41 or older	Negative
74895049	good	3AB	31 to 32	Positive
39453539	good	3BB	31 to 32	Positive
58562091	poor	2.5BB-	39 to 40	Negative
13490370	poor	3BB-	39 to 40	Negative
73510479	good	3AB	31 to 32	Negative
47719639	poor	3BB	31 to 32	Negative

40086121	good	3AB	31 to 32	Negative
95008841	good	3AB	30 or younger	Positive
87733195	good	3BB	33 to 34	Negative
41218015	good	3A-B	33 to 34	Negative
39701181	good	3BB	33 to 34	Positive
69901712	good	3BB	33 to 34	Positive
87319899	good	3A-A-	35 to 36	Negative
69500741	poor	1BB	37 to 38	Positive
32689099	poor	2BB-	30 or younger	Negative
53785767	poor	3B-B	30 or younger	Negative
98740714	good	3BB	30 or younger	Negative
51470305	good	3BB	35 to 36	Positive
59623419	good	3A-B	35 to 36	Positive
34135097	good	3A-B	35 to 36	Negative
37873606	poor	2BB-	35 to 36	Negative
88079640	good	3BB	30 or younger	Negative
87136749	poor	2BB	30 or younger	Negative
16801241	poor	1BB	30 or younger	Positive
83040091	poor	2BB-	35 to 36	Negative
25622003	poor	3A-B	30 or younger	Positive
33662960	good	3A-A-	30 or younger	Positive
73132993	good	3A-B	30 or younger	Negative
17231777	poor	2B-B-	41 or older	Negative
47679455	good	3AB	35 to 36	Positive
13579733	good	3BB	35 to 36	Positive
91335500	good	3BB	31 to 32	Positive
75787229	poor	3BB-	31 to 32	Negative
70590220	good	3A-B	30 or younger	Positive
19484263	poor	3BB	37 to 38	Positive
35959225	good	3A-B	37 to 38	Positive
61366278	good	3AB	33 to 34	Positive
38326943	poor	2BB	33 to 34	Positive
13830695	poor	2B-B	33 to 34	Positive
32903221	good	3A-B	33 to 34	Positive
96917745	good	3AA	30 or younger	Negative
54350732	poor	1BB	39 to 40	Positive
87221438	poor	1.5BB-	37 to 38	Negative
86010840	poor	3BB-	37 to 38	Negative
87042447	good	3BB	37 to 38	Negative
45309983	good	3BB	33 to 34	Positive
90197627	good	3BA-	33 to 34	Negative
43889366	good	3BB	33 to 34	Negative

72196125	good	4AA-	30 or younger	Positive
81558611	good	3BB	30 or younger	Negative
28451640	good	3A-A-	30 or younger	Positive
30798035	good	3A-B	30 or younger	Negative
16109665	poor	3B-B	35 to 36	Positive
84724340	poor	3BB-	41 or older	Positive
75126943	good	3A-A-	35 to 36	Positive
13761804	poor	3B-B	41 or older	Negative
85306126	good	3A-B	41 or older	Negative
18114395	poor	3B-B	41 or older	Negative
11032986	poor	3BB-	41 or older	Negative
43830936	poor	2BB	41 or older	Negative
44218676	good	3A-A-	33 to 34	Positive
43051266	good	3AA-	33 to 34	Positive
70075209	good	3A-B	39 to 40	Positive
59631813	good	3A-A-	31 to 32	Positive
39573348	poor	2BB	35 to 36	Positive
88972465	poor	3BB	31 to 32	Positive
74426288	poor	3BB-	39 to 40	Negative
62158249	poor	3BB-	39 to 40	Negative
73624854	good	3A-A-	35 to 36	Negative
60772938	good	3A-B	35 to 36	Positive
35579207	poor	3A-B	35 to 36	Positive
44554772	good	3BB	39 to 40	Negative
17792533	good	3A-B	39 to 40	Negative
28940817	poor	3BB-	39 to 40	Negative
74452593	good	3A-B	35 to 36	Positive
90547380	good	3AB	30 or younger	Positive
17368274	good	3BB	31 to 32	Positive
89270762	good	3AA	30 or younger	Negative
90131916	good	3A-A-	30 or younger	Negative
26023351	good	3A-A-	30 or younger	Negative
24164396	good	3A-A-	35 to 36	Positive
93872383	good	3A-A-	31 to 32	Negative
64136954	poor	3BB	35 to 36	Negative
18964251	poor	3A-B	35 to 36	Negative
19859182	good	3A-B	30 or younger	Negative
40163909	poor	3B-B-	30 or younger	Negative
53513291	poor	2B-B-	30 or younger	Negative
63052475	poor	3B-B	30 or younger	Negative
52609664	poor	3B-B	30 or younger	Negative
93170952	good	3A-B	33 to 34	Negative

95100906	good	3A-A-	41 or older	Positive
12072820	good	3BB	41 or older	Positive
52954306	good	3A-B	35 to 36	Negative
49841035	poor	3BB-	35 to 36	Positive
52492792	good	3BB	35 to 36	Positive
95913988	good	3AB	35 to 36	Negative
86970575	good	3BB	35 to 36	Positive
59288767	good	3AA-	33 to 34	Negative
72575165	good	3A-A-	33 to 34	Positive
66006342	good	3AB	33 to 34	Positive
87543007	good	3AA-	31 to 32	Negative
17859271	good	3BB	31 to 32	Positive
52089098	good	3A-B	31 to 32	Negative
28989886	good	3A-A	37 to 38	Positive
80630229	poor	3BB	33 to 34	Positive
45679139	poor	3B-B-	33 to 34	Positive
14906261	good	3A-B	35 to 36	Positive
10665447	poor	2.5BB-	39 to 40	Negative
49350596	good	3AB	33 to 34	Positive
82733248	good	3AA	33 to 34	Positive
20042371	good	3BB	37 to 38	Negative
47831549	good	3AB	39 to 40	Positive
85903978	good	3BA-	39 to 40	Positive
96430346	good	3AA-	35 to 36	Positive
11848058	good	3BA-	30 or younger	Positive
19334426	good	3A-A-	30 or younger	Positive
65664236	good	3AA	39 to 40	Negative
16390514	good	3AA	39 to 40	Negative
35784576	good	3AA	33 to 34	Negative
93417607	good	3AA-	33 to 34	Positive
71172437	poor	3BB-	31 to 32	Positive
10497937	poor	3BB-	31 to 32	Positive
58298461	poor	3A-B	31 to 32	Negative
62230015	poor	1.5BB	31 to 32	Positive
82685134	good	3A-B	31 to 32	Negative
72112290	good	3A-A-	33 to 34	Positive
76005670	good	3AA	35 to 36	Positive
35339584	good	3A-B	35 to 36	Negative
27340137	poor	2B-B	33 to 34	Positive
84184749	good	3BB	30 or younger	Negative
91093071	good	3A-B	30 or younger	Negative
22246179	good	3A-B	33 to 34	Negative

23928993	poor	1B-B-	33 to 34	Negative
20450641	good	3A-B	33 to 34	Positive
31772222	good	3AA	33 to 34	Positive
86183620	good	3A-A	33 to 34	Positive
23109092	good	3BB	30 or younger	Positive
68896545	poor	3BB-	33 to 34	Negative
49649727	poor	2BB-	33 to 34	Positive
95319525	poor	2BB	33 to 34	Negative
58529282	good	3AA-	39 to 40	Negative
71783785	poor	2BB	39 to 40	Negative
55559661	poor	3BB-	37 to 38	Negative
29532053	good	3AB	37 to 38	Negative
30984599	poor	2B-B-	41 or older	Positive
77852541	poor	2BB-	31 to 32	Negative
82745365	poor	3BB-	31 to 32	Negative
32779492	poor	1B-B-	30 or younger	Positive
14735360	good	3BA-	39 to 40	Negative
81204898	poor	2B-B	39 to 40	Positive
32840637	poor	3BB-	39 to 40	Negative
24389172	poor	3BB-	33 to 34	Negative
52079937	poor	1BB	33 to 34	Positive
80816354	good	3A-A	33 to 34	Negative
55487091	poor	3B-B-	35 to 36	Negative
97758912	poor	1BB-	30 or younger	Negative
29580747	good	3A-A-	33 to 34	Negative
38123386	poor	3B-B	33 to 34	Negative
58572456	poor	2BB	33 to 34	Negative
97148305	good	3BA-	33 to 34	Negative
74346803	poor	3BB-	31 to 32	Positive
91912253	good	3AB	31 to 32	Negative
84308253	poor	3BB-	31 to 32	Negative
67003719	good	3A-A-	33 to 34	Negative
39694169	poor	2B-B-	30 or younger	Positive
67391791	poor	3BB	30 or younger	Positive
49376708	good	3BB	30 or younger	Positive
65301439	good	3A-B	30 or younger	Positive
64137521	good	3AB	35 to 36	Positive
72047826	good	3AB	30 or younger	Positive
67777876	poor	2BB	33 to 34	Negative
83649160	good	3A-A-	33 to 34	Positive
96885826	good	3AA-	37 to 38	Positive
78034165	good	3AA-	37 to 38	Positive

60547113	good	3AA	30 or younger	Positive
15697722	good	3AA-	37 to 38	Negative
91948421	good	3A-B	37 to 38	Positive
72123729	good	3AB	37 to 38	Positive
29460817	good	3AB	39 to 40	Positive
50554470	good	3AA	33 to 34	Negative
68455772	poor	1B-B-	41 or older	Negative
89832127	good	3BA-	41 or older	Negative
89252303	good	3A-B	37 to 38	Negative
28713587	good	3A-B	37 to 38	Positive
82290206	poor	3BB-	39 to 40	Positive
36292714	good	3AA-	39 to 40	Negative
52855711	poor	3BB-	39 to 40	Positive
64889943	poor	3B-B-	39 to 40	Positive
54693458	good	3BA-	37 to 38	Positive
58022869	good	3A-A-	37 to 38	Negative
17453353	good	3BB	37 to 38	Positive
94234253	poor	2BB-	35 to 36	Negative
41019399	poor	1B-B-	39 to 40	Negative
49369484	good	3BB	30 or younger	Negative
25320206	good	3AB	37 to 38	Negative
20940556	good	3BB	37 to 38	Positive
92316287	poor	2.5B-B-	39 to 40	Negative
22881446	good	3A-B	39 to 40	Negative
78886489	good	3BB	35 to 36	Negative
92215680	poor	3B-B	35 to 36	Negative
51875278	good	3AA	31 to 32	Negative
55893432	good	3BB	33 to 34	Positive
30734171	good	3AA	31 to 32	Positive
94221011	good	3AA-	33 to 34	Positive
22682267	good	3AB	41 or older	Negative
39740677	good	3AA	35 to 36	Negative
55615605	good	3BB	37 to 38	Positive
92126570	poor	1.5BB-	37 to 38	Negative
48556337	poor	1.5BB	37 to 38	Negative
36933247	poor	1BB	37 to 38	Negative
19981558	poor	1.5B-B-	30 or younger	Negative
22623141	poor	1B-B-	30 or younger	Positive
74819118	good	3AA	30 or younger	Positive
87399002	good	3AA-	31 to 32	Positive
99207700	good	3AA	30 or younger	Negative
27674299	good	3BB	31 to 32	Positive

77606477	good	3AB	30 or younger	Negative
13397491	good	3BB	30 or younger	Negative
59151180	good	3AB	39 to 40	Positive
25918380	poor	3B-B	30 or younger	Positive
24387510	good	3A-B	35 to 36	Positive
84710770	poor	1BB	33 to 34	Negative
69220308	good	3A-B	31 to 32	Positive
36338476	good	3AA	33 to 34	Positive
61549780	good	3AA-	35 to 36	Positive
99758375	good	3AA-	35 to 36	Positive
73250818	good	3BB	30 or younger	Negative
76307434	poor	2BB	30 or younger	Positive
44567947	good	3A-B	35 to 36	Positive
30121825	poor	1BB-	39 to 40	Negative
80709288	good	3A-B	37 to 38	Negative
50858110	poor	3BB-	37 to 38	Negative
68062287	good	3AA-	35 to 36	Negative
26723343	good	3A-B	35 to 36	Negative
76800269	poor	2B-B	37 to 38	Positive
49991423	poor	3BB-	33 to 34	Positive
26406580	poor	3BB	33 to 34	Positive
77454540	good	3AA	31 to 32	Positive
50348579	good	3AB	37 to 38	Positive
40959582	good	3BB	37 to 38	Positive
17861406	poor	3B-B-	39 to 40	Negative
72156647	poor	1BB-	33 to 34	Negative
29752331	good	3AA	33 to 34	Negative
53978030	poor	3BB-	33 to 34	Negative
65596636	good	3BB	33 to 34	Negative
49469808	good	3A-B	30 or younger	Negative
26064180	poor	3A-B	33 to 34	Negative
13831587	good	3AB	31 to 32	Positive
97008995	good	3AA-	37 to 38	Positive
87369799	good	3AB	37 to 38	Positive
87533878	good	3AB	33 to 34	Positive
45774247	good	3BB	30 or younger	Negative
90097656	poor	3A-B	35 to 36	Positive
70462406	good	3AB	30 or younger	Positive
77447241	poor	1.5BB	35 to 36	Negative
19131622	poor	2BB-	35 to 36	Negative
64112834	good	3A-B	35 to 36	Negative
49178484	poor	3B-B	35 to 36	Negative

38936479	good	3AA-	35 to 36	Negative
38397957	good	3AB	35 to 36	Positive
95294385	good	3A-A-	31 to 32	Positive
38276512	good	3A-A-	31 to 32	Negative
94604373	good	3A-B	33 to 34	Positive
77115724	good	3A-B	33 to 34	Negative
48340947	good	3AB	37 to 38	Positive
26083938	good	3A-A-	37 to 38	Positive
88830533	good	3BB	31 to 32	Positive
69556143	good	3BA-	31 to 32	Positive
99284152	poor	1BB-	33 to 34	Positive
18100962	good	3A-B	35 to 36	Positive
76082993	good	3AA	35 to 36	Positive
56177868	poor	2BB	33 to 34	Negative
10657796	poor	2.5B-B-	39 to 40	Negative
54746027	good	3A-B	39 to 40	Negative
76110622	good	3A-B	39 to 40	Negative
74417817	good	3AB	37 to 38	Positive
78242925	good	3AA	37 to 38	Positive
83386749	good	3BA-	35 to 36	Positive
69599850	good	3A-A-	35 to 36	Positive
30009452	poor	3BB	30 or younger	Positive
19713078	good	3BB	31 to 32	Negative
49944856	good	3A-B	31 to 32	Negative
47118384	good	3BB	31 to 32	Negative
37037261	good	3BB	31 to 32	Negative
82699950	poor	3BB-	37 to 38	Negative
32898300	poor	1B-B-	33 to 34	Negative
60930978	good	3BB	33 to 34	Negative
37896220	poor	3B-B-	33 to 34	Negative
61900763	poor	2.5B-B-	33 to 34	Negative
69971789	poor	2.5BB-	31 to 32	Negative
36946788	poor	2BB-	31 to 32	Negative
72823286	poor	3B-B	37 to 38	Positive
97280136	poor	3B-B-	37 to 38	Positive
78815787	good	3AB	31 to 32	Positive
33070473	good	3AB	30 or younger	Positive
83116444	good	3BB	30 or younger	Positive
56941234	good	3AA-	33 to 34	Positive
31344666	poor	2BB-	33 to 34	Positive
60446769	good	3AA-	37 to 38	Negative
12806939	good	3A-B	31 to 32	Positive

52900129	poor	3BB-	35 to 36	Negative
49281297	poor	3BB-	35 to 36	Negative
42465813	good	3A-B	39 to 40	Negative
41176874	poor	3B-B	39 to 40	Negative
99607893	good	3A-A-	37 to 38	Negative
64103700	poor	1B-B-	30 or younger	Positive
64285120	good	3AB	30 or younger	Negative
19066674	good	3A-B	35 to 36	Negative
26932333	poor	3BB-	41 or older	Negative
53688381	poor	2BB	35 to 36	Positive
99012950	good	3BB	35 to 36	Positive
86507472	good	3AB	33 to 34	Positive
90556711	good	3AA	33 to 34	Positive
50461296	good	3AB	33 to 34	Positive
43563405	poor	1BB-	39 to 40	Negative
90436015	good	3AA-	31 to 32	Negative
98704163	good	3A-A-	31 to 32	Negative
73018917	good	3BA-	31 to 32	Positive
39996825	poor	3A-B	31 to 32	Positive
48857364	good	3A-B	31 to 32	Positive
11486813	good	3AA-	33 to 34	Positive
61230868	good	3BB	33 to 34	Positive
76931237	good	3BB	33 to 34	Positive
67629128	poor	3B-B-	30 or younger	Negative
83171367	poor	3BB-	30 or younger	Negative
49976120	good	3A-B	31 to 32	Positive
57873931	good	3A-A-	31 to 32	Negative
46732274	good	3AB	31 to 32	Positive
16428794	poor	2BB-	33 to 34	Negative
54687439	good	3BB	33 to 34	Negative
44494608	good	3AB	33 to 34	Positive
72574076	poor	1BB	31 to 32	Negative
83054468	poor	1BB	31 to 32	Negative
46016022	poor	2BB	31 to 32	Positive
33890272	poor	2BB-	41 or older	Positive
43420231	good	3A-A-	37 to 38	Negative
68124687	good	3BB	37 to 38	Negative
37949730	good	3BB	37 to 38	Negative
14763737	poor	3BB-	30 or younger	Negative
68770029	good	3AA-	33 to 34	Positive
25959926	poor	3B-B-	30 or younger	Positive
45645348	good	3BB	33 to 34	Positive

14469741	good	3AB	30 or younger	Positive
70638959	good	3A-B	30 or younger	Positive
43425619	good	3AB	33 to 34	Positive
96914603	poor	2.5B-B	35 to 36	Negative
77390363	poor	1BB-	41 or older	Negative
37248564	good	3BB	41 or older	Positive
46413145	good	3AA	41 or older	Positive
75844149	good	3AA-	33 to 34	Negative
71051570	poor	1B-B-	41 or older	Positive
28249987	poor	2B-B-/C	41 or older	Positive
21771420	good	3AB	33 to 34	Negative
54463556	poor	3B-B-	33 to 34	Positive
99053170	good	3AB	37 to 38	Positive
87529226	good	3AA	31 to 32	Positive
32101024	good	3BB	39 to 40	Positive
67415626	good	3BB	41 or older	Negative
36010328	good	3A-A-	37 to 38	Positive
41155378	good	3BB	33 to 34	Positive
46583992	poor	2B-B-	31 to 32	Negative
49051896	poor	1.5B-B-	35 to 36	Negative
69287457	poor	2.5BB-	35 to 36	Positive
33174164	good	3BB	35 to 36	Positive
39021179	poor	3B-B	35 to 36	Negative
27579491	poor	3B-B	35 to 36	Positive
98914316	good	3A-B	33 to 34	Positive
48839173	good	3AB	30 or younger	Positive
22906109	poor	3BB-	30 or younger	Negative
73642267	good	3BB	30 or younger	Negative
10180916	good	3AA-	31 to 32	Positive
22794628	poor	3B-B	33 to 34	Positive
82482237	good	3AA	31 to 32	Positive
25300259	good	3AB	33 to 34	Positive
49270948	good	3AA-	30 or younger	Positive
63714382	good	3AA-	30 or younger	Negative
19449529	good	3AA-	30 or younger	Negative
41045667	poor	2BB	31 to 32	Positive
87144713	good	3A-A-	37 to 38	Negative
99996651	good	3A-A-	37 to 38	Negative
26356717	good	3A-B	31 to 32	Positive
61016038	good	3A-B	35 to 36	Negative
30566024	poor	2BB	39 to 40	Positive
98894923	good	3AA	37 to 38	Positive

97964460	poor	2.5BB-	31 to 32	Positive
72312497	poor	2BB	31 to 32	Positive
57792278	good	3AA-	31 to 32	Negative
15065344	poor	1BB	30 or younger	Positive
59820246	poor	2BB	30 or younger	Positive
97389584	poor	2BB	30 or younger	Negative
94311015	good	3AA-	30 or younger	Negative
55083539	good	3BB	31 to 32	Positive
18570412	good	3AB	31 to 32	Negative
47693792	good	3AB	31 to 32	Positive
90655237	good	4AB	35 to 36	Positive
60776333	good	3BB	35 to 36	Positive
91800456	good	3BB	35 to 36	Negative
37515583	poor	3BB-	35 to 36	Negative
60240856	poor	2BB	33 to 34	Positive
99763130	good	3AB	33 to 34	Negative
17157058	good	3A-B	31 to 32	Positive
15578261	good	3BB	35 to 36	Negative
94323073	poor	2BB	33 to 34	Negative
75182688	poor	2B-B	33 to 34	Negative
42014345	poor	3BB-	33 to 34	Negative
73488832	poor	3BB-	33 to 34	Negative
89420672	poor	3B-B-	33 to 34	Negative
80087882	good	3BB	33 to 34	Negative
14516568	poor	3BB	39 to 40	Negative
63233850	good	3BB	33 to 34	Positive
12343401	poor	2BB	30 or younger	Negative
25759852	poor	2BB	30 or younger	Negative
92691821	poor	2B-B	39 to 40	Negative
37647569	good	3BB	39 to 40	Negative
88903848	good	3AB	35 to 36	Positive
93321876	poor	1.5B-B	35 to 36	Positive
92921469	poor	3B-B	35 to 36	Positive
39867514	good	3A-B	37 to 38	Negative
10459671	good	3A-B	37 to 38	Negative
49568986	good	3A-A-	30 or younger	Negative
17072482	poor	2BB	31 to 32	Negative
58215177	poor	2BB-/C	31 to 32	Negative
94919261	good	3AB	33 to 34	Positive
51449983	poor	1B-B	35 to 36	Positive
65616408	good	3BB	41 or older	Negative
87287144	poor	3B-B	41 or older	Negative

73354590	poor	2BB-	39 to 40	Negative
36965847	poor	1.5B-B	39 to 40	Negative
33270733	poor	1.5BB	39 to 40	Positive
86977806	poor	2BB	39 to 40	Negative
38694946	poor	1BB	39 to 40	Negative
97935961	poor	1BB	39 to 40	Positive
79171176	poor	1BB-	31 to 32	Negative
82155782	poor	2BB	31 to 32	Negative
22698424	good	3AA	35 to 36	Positive
51383770	good	3A-A	33 to 34	Positive
71582089	good	3AA-	30 or younger	Negative
84730242	poor	3A-B	35 to 36	Positive
33007786	good	3BB	30 or younger	Positive
46014486	good	3A-B	35 to 36	Positive
69407497	good	3BB	41 or older	Positive
26950519	good	3BB	37 to 38	Positive
44155279	good	3A-A-	37 to 38	Positive
90189632	good	3AA	37 to 38	Positive
86756955	good	3A-A-	35 to 36	Positive
61434021	poor	3BB	37 to 38	Negative
51407081	poor	1B-B	33 to 34	Positive
56169298	good	3A-B	33 to 34	Positive
17066800	good	3BB	33 to 34	Positive
76156339	good	3AB	37 to 38	Negative
19079428	good	3A-A-	31 to 32	Positive
85117626	good	3AA	33 to 34	Positive
40331132	good	3BB	33 to 34	Positive
37407022	good	3AB	33 to 34	Positive
10144703	poor	3B-/CB-/C	33 to 34	Negative
19113441	good	3A-A	31 to 32	Positive
83629179	good	3A-A-	30 or younger	Positive
48500243	good	3A-A-	39 to 40	Positive
12611071	good	3BB	35 to 36	Positive
31497441	poor	3BB	35 to 36	Positive
81516054	good	3A-B	31 to 32	Positive
40365308	poor	2.5BB-	37 to 38	Negative
54378073	good	3BB	37 to 38	Positive
20513248	poor	2BB	37 to 38	Negative
69225976	poor	2B-B	35 to 36	Positive
54931258	poor	3BB	35 to 36	Positive
47135010	good	3A-B	31 to 32	Positive
39614097	good	3AB	31 to 32	Positive

31990887	good	3BB	39 to 40	Negative
55512502	good	3BB	31 to 32	Positive
93041983	good	3BB	31 to 32	Negative
60463694	poor	1BB	37 to 38	Negative
40710319	poor	1.5BB	39 to 40	Negative
10069317	good	3BB	33 to 34	Negative
58972262	poor	3B-B-	41 or older	Positive
84743654	good	3BB	31 to 32	Negative
78236513	poor	2B-B	31 to 32	Positive
10240519	poor	1BB	31 to 32	Positive
32151951	good	3A-A-	31 to 32	Negative
68145032	good	3AA	30 or younger	Positive
87541867	good	3BA-	31 to 32	Positive
73728890	good	3A-B	31 to 32	Positive
33805182	good	3BB	31 to 32	Positive
92016396	good	3AA	31 to 32	Positive
45083766	poor	1.5BB	35 to 36	Positive
78686831	good	3A-B	35 to 36	Positive
30064601	good	3BB	35 to 36	Negative
73236573	poor	3B-B	35 to 36	Negative
29200231	good	3BB	35 to 36	Negative
23293874	poor	1.5BB-	33 to 34	Positive
92702679	poor	2BB-	31 to 32	Negative
21563957	poor	1BB	37 to 38	Negative
51422443	good	3AA-	31 to 32	Positive
78821331	good	3A-B	39 to 40	Positive
49342549	good	3BB	33 to 34	Negative
20891208	good	3BA-	33 to 34	Negative
16130067	good	3A-A-	33 to 34	Negative
56518412	good	3A-B	30 or younger	Positive
62838776	poor	2BB	37 to 38	Negative
22587942	good	3AA	30 or younger	Positive
16433634	good	3A-A-	35 to 36	Positive
48649567	good	3AA-	35 to 36	Positive
62517421	poor	3BB-	33 to 34	Negative
48967847	poor	3BB-	33 to 34	Positive
95108350	good	3AA	31 to 32	Positive
75023326	good	3BB	31 to 32	Negative
77807985	good	3A-B	39 to 40	Negative
84473399	poor	3B-B-	39 to 40	Negative
57287531	poor	1BB-	37 to 38	Negative
19023133	poor	3BB	30 or younger	Positive

77344747	good	3A-B	35 to 36	Positive
83453674	poor	3BB-	35 to 36	Positive
75193937	good	3A-A-	41 or older	Negative
51796993	good	3BA-	41 or older	Negative
72623537	poor	1.5BB	37 to 38	Negative
10557206	poor	3B-/CB-	37 to 38	Negative
39979676	poor	1B-B	37 to 38	Negative
34909211	poor	1B-B	37 to 38	Negative
58505154	good	3A-A-	39 to 40	Negative
25763238	good	3A-B	33 to 34	Negative
16963025	good	3AA-	30 or younger	Positive
34656175	good	3A-A-	30 or younger	Positive
73080286	poor	2BB	35 to 36	Negative
41704497	good	3AB	30 or younger	Positive
63269114	good	3A-A-	37 to 38	Positive
48958067	good	3AA	33 to 34	Positive
98214292	good	3A-B	30 or younger	Negative
73290464	good	3BB	30 or younger	Positive
20524464	good	3A-B	30 or younger	Negative
33323888	good	3A-A-	33 to 34	Positive
55632253	good	3A-B	33 to 34	Positive
61620414	good	3A-B	35 to 36	Positive
51445439	good	4AB	30 or younger	Positive
78786554	good	3BB	35 to 36	Positive
24583562	good	3BA-	30 or younger	Positive
28432994	good	3BB	33 to 34	Positive
29523710	good	3BB	33 to 34	Negative
11846197	good	3A-A-	33 to 34	Positive
38465392	good	3AB	31 to 32	Negative
46927842	good	3AA-	33 to 34	Negative
22867262	good	3A-B	35 to 36	Positive
46846364	poor	2BB	30 or younger	Negative
32611406	good	3A-B	35 to 36	Positive
13165946	poor	1BB-	37 to 38	Positive
54443883	good	3A-B	37 to 38	Positive
28770149	poor	3B-B-	31 to 32	Positive
21119982	good	3BB	37 to 38	Positive
73292523	poor	2BB	33 to 34	Positive
50624829	poor	3BB	33 to 34	Negative
56984737	good	3AB	33 to 34	Positive
60671645	good	3AA-	33 to 34	Negative
58458699	poor	2B-B-	30 or younger	Positive

38538755	poor	1BB-	30 or younger	Positive
26290386	good	3BB	37 to 38	Positive
65893328	poor	3BB	30 or younger	Positive
20338026	good	3A-B	30 or younger	Negative
15892653	good	3A-A-	30 or younger	Positive
79424036	good	3BB	31 to 32	Negative
60835187	poor	3BB-	31 to 32	Positive
70102295	good	3A-B	30 or younger	Negative
32715556	good	3A-A-	30 or younger	Negative
43227519	good	3A-B	30 or younger	Positive
31916872	good	3A-B	30 or younger	Positive
73703907	good	3AB	37 to 38	Positive
65011417	good	3A-B	33 to 34	Positive
74415775	good	3A-A-	33 to 34	Positive
27784871	poor	3BB	39 to 40	Positive
24476438	good	3AA-	35 to 36	Positive
12634236	good	3A-A-	30 or younger	Positive
28453570	good	3A-B	31 to 32	Positive
86605521	poor	3B-B	39 to 40	Positive
22108851	good	3A-B	33 to 34	Negative
77618804	good	3A-A-	33 to 34	Negative
44604006	good	3A-B	33 to 34	Positive
21370868	good	3A-B	31 to 32	Positive
80862426	poor	3A-B	39 to 40	Positive
63424460	good	3A-B	39 to 40	Negative
15722433	good	3BB	37 to 38	Positive
40131626	good	3A-A-	30 or younger	Positive
54735099	good	3A-B	31 to 32	Positive
80509514	good	3A-A-	31 to 32	Positive
89946588	poor	2BB	35 to 36	Positive
64310049	good	3BB	35 to 36	Positive
76880500	good	3BB	35 to 36	Negative
38429554	poor	3BB-	35 to 36	Positive
98260345	poor	1.5BB	33 to 34	Negative
80321976	poor	1B-B-	33 to 34	Negative
72942833	poor	1B-C	41 or older	Negative
10720322	good	3AB	35 to 36	Positive
43278985	good	3AB	35 to 36	Positive
16365159	poor	3BB	35 to 36	Negative
22511811	poor	3BB	35 to 36	Negative
66871748	good	3A-B	35 to 36	Negative
98718462	good	3BB	35 to 36	Negative

25441109	good	3A-B	35 to 36	Negative
90729857	poor	2BB-	35 to 36	Negative
61883224	poor	3A-B	35 to 36	Negative
74344358	good	3A-B	35 to 36	Negative
37774990	good	3A-A-	35 to 36	Negative
46190748	good	3A-A-	35 to 36	Negative
91626788	poor	2BB	35 to 36	Negative
75079702	poor	1B-B-	35 to 36	Negative
34107362	good	3AA-	39 to 40	Positive
72968040	good	3AA-	39 to 40	Positive
37116005	poor	3BB-	37 to 38	Positive
28496330	poor	3BB-	41 or older	Negative
63341068	good	3A-B	41 or older	Negative
88910342	poor	3BB-	41 or older	Negative
95625983	poor	3BB-	35 to 36	Negative
64647292	poor	1.5BB-	35 to 36	Positive
51596059	poor	1BB	35 to 36	Positive
94151163	poor	2.5BB-	33 to 34	Negative
32214769	good	3AB	35 to 36	Positive
33772089	good	3A-B	35 to 36	Positive
93703581	good	3AB	39 to 40	Negative
55213789	good	3BB	39 to 40	Positive
61390100	poor	2BB	35 to 36	Positive
15130446	good	3A-B	31 to 32	Positive
92774782	good	3AA-	30 or younger	Positive
17641234	good	3A-A-	31 to 32	Positive
52285894	good	3A-A-	35 to 36	Negative
47659595	good	3A-A-	33 to 34	Positive
71254297	poor	1B-B-/C	41 or older	Negative
38859640	poor	2B-B-/C	41 or older	Negative
69663908	poor	3B-C	41 or older	Negative
92587282	poor	2BB-	41 or older	Negative
19499995	poor	1.5BB-	41 or older	Negative
35096606	good	3A-A	35 to 36	Negative
38738112	good	3BB	30 or younger	Positive
67862702	good	3AB	39 to 40	Positive
13211524	poor	1.5BB-	33 to 34	Positive
86810943	good	3A-B	31 to 32	Positive
77854742	good	3BB	37 to 38	Positive
83513645	good	3A-B	37 to 38	Positive
27863656	good	3BB	33 to 34	Negative
46293292	good	3AA-	35 to 36	Negative

88325539	good	3BB	33 to 34	Positive
10566201	good	3BB	33 to 34	Positive
41586576	poor	2B-/CB-	41 or older	Negative
28161414	poor	1.5BB-	41 or older	Negative
74461506	poor	3BB-/C	41 or older	Negative
24677735	good	3A-B	33 to 34	Negative
84577039	good	3A-B	35 to 36	Positive
85729229	poor	2B-B-	39 to 40	Negative
46241693	poor	3BB	39 to 40	Negative
67173806	good	3A-B	31 to 32	Negative
98307333	good	3BB	31 to 32	Positive
15867411	good	3A-B	31 to 32	Positive
98481605	good	3BB	35 to 36	Positive
97000633	good	3A-B	31 to 32	Positive
94565276	poor	3B-B-	41 or older	Negative
40816696	poor	3BB-	41 or older	Negative
85215342	poor	1BB	41 or older	Negative
55703312	good	3A-A-	30 or younger	Negative
69007694	poor	3B-B	37 to 38	Positive
21529534	good	3A-A-	31 to 32	Positive
99222798	good	3BB	31 to 32	Positive
21967227	good	3BB	30 or younger	Positive
55022998	good	3BB	31 to 32	Positive
39650879	poor	1.5BB	35 to 36	Negative
82127295	poor	1BB-	35 to 36	Negative
85298275	good	3A-B	35 to 36	Negative
55411679	good	3AA	33 to 34	Positive
28043433	good	3A-A-	30 or younger	Negative
86491524	good	3A-B	37 to 38	Negative
45787199	good	3BB	30 or younger	Negative
76632416	good	3BB	30 or younger	Positive
93309587	poor	3BB-	30 or younger	Positive
18678824	good	3A-B	35 to 36	Positive
62569462	good	3A-B	33 to 34	Positive
94663643	good	3A-A-	30 or younger	Positive
39687159	good	3AB	41 or older	Positive
57166073	good	3A-B	30 or younger	Positive
52988374	good	3A-A-	30 or younger	Negative
89065879	good	3BB	30 or younger	Positive
25036135	good	3BB	30 or younger	Positive
71409150	good	3A-A-	30 or younger	Negative
18886535	good	3AA	31 to 32	Positive

47007703	poor	3BB-	41 or older	Negative
92475782	poor	3BB-	41 or older	Negative
62504597	poor	3B-B	41 or older	Negative
78060518	poor	3BB	41 or older	Negative
42349327	good	3AB	41 or older	Positive
72838517	good	3AB	41 or older	Positive
43574952	good	3BB	37 to 38	Positive
54345658	poor	3BB	37 to 38	Positive
42411415	good	3BB	33 to 34	Negative
26569532	good	3BB	35 to 36	Positive
49177335	poor	3BB-	33 to 34	Positive
73667612	good	3BB	33 to 34	Negative
82080499	good	3BB	31 to 32	Positive
79645483	good	3BB	31 to 32	Positive
49496472	good	3AA-	39 to 40	Negative
84504417	good	3A-A	35 to 36	Positive
96154450	good	3BB	37 to 38	Negative
31064757	good	3A-A	37 to 38	Negative
47141742	good	3A-B	30 or younger	Positive
76464216	good	3AA-	31 to 32	Positive
11745488	good	3BB	35 to 36	Positive
51237147	poor	2BB	30 or younger	Negative
38674682	good	3BB	30 or younger	Negative
93198820	poor	3BB-	30 or younger	Negative
78965838	good	3BB	30 or younger	Negative
46002271	good	3A-B	41 or older	Negative
28667965	good	3BB	41 or older	Negative
81062603	good	3A-A-	39 to 40	Negative
11960451	poor	3B-B	39 to 40	Negative
13732028	good	3BB	39 to 40	Negative
90107206	good	3A-A-	33 to 34	Negative
42009465	good	3A-A-	41 or older	Negative
53300725	good	3BA-	39 to 40	Negative
94071806	good	3A-A-	33 to 34	Positive
28944861	good	3A-A-	33 to 34	Positive
83695582	good	3A-A-	37 to 38	Positive
48104249	good	3A-A-	33 to 34	Positive
31727305	poor	2BB	35 to 36	Positive
91335774	poor	2BB	35 to 36	Positive
86984097	good	3AB	39 to 40	Negative
55602743	good	3BB	39 to 40	Negative
77074033	good	3A-A-	31 to 32	Positive

63556455	poor	1BB-	30 or younger	Negative
28354163	good	3AA	37 to 38	Positive
37096567	good	3BB	31 to 32	Positive
31493485	good	3A-B	39 to 40	Positive
56752925	good	3BB	39 to 40	Negative
36132785	good	3BA-	35 to 36	Positive
42611408	good	3A-B	35 to 36	Positive
86655492	poor	3BB	41 or older	Positive
75188319	good	3BB	31 to 32	Positive
12338517	poor	2BB	33 to 34	Positive
31431197	good	3A-B	33 to 34	Positive
65109836	good	3BB	33 to 34	Negative
97083804	poor	3B-B-	37 to 38	Negative
55454738	poor	3B-B	37 to 38	Negative
86958229	good	3A-B	37 to 38	Negative
22841763	poor	2BB-	35 to 36	Negative
45128646	poor	2BB	35 to 36	Negative
96168745	good	3BB	35 to 36	Negative
17722816	good	3BB	35 to 36	Negative
45175943	good	3A-A-	33 to 34	Negative
34030355	good	3A-B	33 to 34	Positive
43896209	good	3A-A-	33 to 34	Negative
94887926	poor	3B-B	33 to 34	Positive
59517611	poor	1BB-	39 to 40	Negative
64446656	poor	2B-B-	37 to 38	Negative
88955176	good	3A-B	35 to 36	Positive
22290028	poor	3B-B	39 to 40	Positive
28465063	good	3A-B	39 to 40	Positive
43051468	poor	1B-C	41 or older	Negative
54824150	poor	3A-B	33 to 34	Positive
11513176	good	3BB	37 to 38	Positive
41802347	poor	3BB-	31 to 32	Negative
51672185	good	3AA	35 to 36	Negative
93929389	good	3A-A-	35 to 36	Positive
76537583	good	3A-A-	35 to 36	Positive
13315919	poor	3BB-	39 to 40	Negative
93898884	poor	3B-B-	39 to 40	Negative
20932985	good	3A-A	30 or younger	Positive
19242614	poor	2B-B-/C	37 to 38	Negative
49596096	good	3AA	33 to 34	Positive
82983484	good	3BB	39 to 40	Negative
89169378	good	3BB	39 to 40	Negative

74206843	good	3BB	39 to 40	Negative
91804565	good	3BB	39 to 40	Negative
36008245	poor	3BB-	39 to 40	Negative
31069283	good	3BB	39 to 40	Negative
32077906	good	3A-A-	39 to 40	Negative
23450110	poor	1B-B-	39 to 40	Positive
64701498	good	3A-A	30 or younger	Positive
20972305	good	3A-B	30 or younger	Positive
77568303	good	3AA-	30 or younger	Negative
14082093	good	3BB	35 to 36	Negative
19769453	good	3BB	37 to 38	Positive
10101873	good	3AA-	30 or younger	Positive
55405919	poor	2BB	33 to 34	Negative
40171480	poor	2BB	33 to 34	Negative
17454864	good	3AA-	33 to 34	Negative
55398131	good	3BA-	30 or younger	Negative
54012268	good	3A-A-	33 to 34	Positive
37402042	good	3BA-	31 to 32	Positive
60754678	poor	3B-B	31 to 32	Positive
72006527	poor	3BB	31 to 32	Positive
59388424	good	3A-B	41 or older	Positive
22670846	good	3A-A-	41 or older	Positive
24549095	poor	2BB	35 to 36	Negative
48297454	poor	2.5BB-	35 to 36	Negative
54385016	good	3BB	35 to 36	Positive
14288139	good	3AA-	33 to 34	Positive
90388050	poor	1B-B	33 to 34	Negative
38027426	poor	1BB-	33 to 34	Negative
29228425	good	3BB	31 to 32	Positive
76326381	poor	3BB-	33 to 34	Negative
60385622	good	3A-B	35 to 36	Positive
27910628	poor	2BB-	33 to 34	Negative
91511457	good	3AB	31 to 32	Positive
49000596	poor	2BB	33 to 34	Positive
31464344	poor	3BB-	35 to 36	Positive
36722198	good	3BB	35 to 36	Positive
28476777	good	3AA-	31 to 32	Negative
90668027	good	3AA-	31 to 32	Negative
23175353	good	3A-B	31 to 32	Positive
80675209	poor	2bb-	31 to 32	Positive
54032946	good	3BB	33 to 34	Positive
87181866	good	3A-A-	30 or younger	Negative

99228769	poor	2B-B	30 or younger	Negative
65163529	good	3BB	39 to 40	Positive
17516331	good	3AA-	39 to 40	Positive
29539193	good	3A-B	39 to 40	Positive
18508277	good	3BA-	39 to 40	Positive
19141554	good	3AA-	39 to 40	Negative
63556200	poor	2BB	33 to 34	Positive
27933729	good	3BB	33 to 34	Negative
36787635	poor	1BB-	41 or older	Negative
46259373	good	3BB	33 to 34	Positive
15049031	good	3A-A-	30 or younger	Positive
23710812	good	3AA-	35 to 36	Positive
85474139	good	3AA-	35 to 36	Positive
64998567	good	3AA-	35 to 36	Positive
34337356	good	3AA-	35 to 36	Negative
50664428	good	3BB	30 or younger	Positive
67564952	good	3BB	31 to 32	Positive
87788384	poor	3BB	37 to 38	Negative
69510353	good	3BB	37 to 38	Negative
42118554	poor	2BB-	37 to 38	Positive
78233182	poor	1B-B-	30 or younger	Negative
34374386	poor	3B-B-	30 or younger	Negative
54082801	poor	3B-B-/C	41 or older	Negative
91483995	poor	2B-B-	33 to 34	Negative
30353597	poor	2BB-	33 to 34	Negative
24255986	good	3A-A-	30 or younger	Positive
85168799	good	3A-B	33 to 34	Negative
79324943	good	3A-A-	39 to 40	Negative
88342744	poor	2BB	37 to 38	Negative
78268634	poor	3B-B	41 or older	Positive
39461677	poor	2BB-	33 to 34	Positive
14219084	good	3A-B	31 to 32	Negative
45225589	good	3A-B	31 to 32	Negative
74374837	good	3A-B	31 to 32	Negative
72139681	good	3AA	31 to 32	Negative
72767532	good	3AA	31 to 32	Negative
97825241	good	3AA-	31 to 32	Positive
83083150	good	3AA	31 to 32	Negative
69606543	good	3BB	33 to 34	Positive
16282604	good	3BB	33 to 34	Positive
24203451	good	3BB	37 to 38	Negative
33809324	good	3A-B	37 to 38	Negative

11538496	good	3A-A-	33 to 34	Positive
26573302	good	3AA-	30 or younger	Positive
75515687	good	5A-A-	33 to 34	Positive
25018440	good	3BB	39 to 40	Positive
87551069	poor	2BB	33 to 34	Negative
47102089	poor	2BB	33 to 34	Negative
34576827	good	3A-B	30 or younger	Positive
86132553	good	3A-A-	31 to 32	Positive
15483985	good	3BB	30 or younger	Negative
99173023	good	3A-A-	35 to 36	Negative
92338613	good	3A-A-	35 to 36	Negative
99210072	good	3BB	33 to 34	Positive
54376593	good	3BB	37 to 38	Positive
86598725	good	3BB	41 or older	Negative
74758367	good	3A-A-	30 or younger	Positive
77942783	good	3A-A-	39 to 40	Positive
20185605	good	3AA	39 to 40	Positive
57473683	good	3A-A-	41 or older	Negative
26515344	good	3BB	37 to 38	Negative
26348650	good	3A-A-	37 to 38	Negative
83519830	good	3A-A-	37 to 38	Positive
65748121	good	3BB	33 to 34	Negative
68567166	poor	1BB	33 to 34	Positive
58152873	good	3A-B	33 to 34	Positive
49438613	good	3A-A	33 to 34	Negative
65538336	good	3BB	37 to 38	Positive
74035196	good	3BB	39 to 40	Negative
14668460	good	3AA	33 to 34	Positive
82683303	good	3A-A-	35 to 36	Positive
28306866	good	3BB	31 to 32	Negative
52988247	poor	3BB-	31 to 32	Negative
87637069	good	3BA-	31 to 32	Negative
38442826	good	3AA	33 to 34	Positive
41283107	good	3BB	31 to 32	Positive
18853851	poor	2BB	30 or younger	Negative
55667179	good	3AA-	30 or younger	Negative
71640571	poor	2BB	30 or younger	Negative
57486103	good	3AB	30 or younger	Negative
73371473	poor	1BB	35 to 36	Negative
42613632	good	3BB	35 to 36	Negative
90318419	good	3BB	31 to 32	Negative
59530954	poor	3B-B	33 to 34	Negative

84178013	good	3BB	35 to 36	Negative
32131696	good	3A-A-	30 or younger	Positive
24431194	good	3AA-	33 to 34	Positive
36256381	good	3BB	30 or younger	Positive
30802694	good	3BB	30 or younger	Positive
50576575	good	3A-B	30 or younger	Positive
34394482	good	3AB	30 or younger	Negative
40898701	poor	3BB-	33 to 34	Positive
86688867	good	3BB	31 to 32	Positive
54104774	poor	1BB	37 to 38	Negative
86381767	poor	3BB	31 to 32	Negative
22406496	good	3A-A-	31 to 32	Negative
32524632	poor	3B-B	31 to 32	Negative
88441771	good	3BB	31 to 32	Negative
12124769	poor	3B-B-	31 to 32	Negative
23248188	good	3A-B	31 to 32	Negative
91487611	good	3AA-	31 to 32	Positive
55435430	good	3AA-	35 to 36	Negative
65700982	poor	2BB	35 to 36	Negative
33801054	poor	2BB	35 to 36	Negative
36672968	good	3BB	35 to 36	Negative
37920728	good	3BA-	33 to 34	Negative
21045798	good	3AB	33 to 34	Positive
93742800	poor	1B-B-	31 to 32	Positive
73952696	poor	3B-B-	31 to 32	Positive
21062896	good	3A-A-	33 to 34	Positive
97414138	good	3BB	35 to 36	Positive
80794767	good	3A-A	35 to 36	Positive
75446780	good	3AA-	33 to 34	Positive
46149861	good	3A-B	37 to 38	Positive
28135473	poor	1B-B	33 to 34	Positive
53361652	good	3BB	35 to 36	Positive
41754282	poor	1B-B	35 to 36	Positive
11223270	good	3AA	33 to 34	Positive
47231907	poor	2.5BB-	31 to 32	Positive
13895276	poor	2BB-	35 to 36	Negative
59064051	good	3BA-	35 to 36	Negative
33919531	good	3BA-	33 to 34	Positive
15561021	good	3A-B	33 to 34	Positive
78351197	good	3BB	33 to 34	Positive
88537669	poor	3B-B-	33 to 34	Negative
69995983	good	3BA-	35 to 36	Negative

76490571	good	3AA	31 to 32	Positive
89279604	good	3AA	33 to 34	Positive
15075835	good	3A-A-	33 to 34	Positive
35673329	good	3AA-	41 or older	Negative
23626234	good	3AA	41 or older	Negative
75855331	good	3A-A-	30 or younger	Positive
46616821	poor	2B-B	33 to 34	Positive
29462419	poor	3B-B-	39 to 40	Negative
31604757	good	3A-A	39 to 40	Negative
52007575	good	3BB	39 to 40	Negative
21667179	good	3AB	37 to 38	Positive
56627764	good	3BB	35 to 36	Negative
69646118	poor	1BB	35 to 36	Negative
12097042	good	3AB	33 to 34	Negative
62082813	good	3A-A-	33 to 34	Negative
26475099	good	3A-A-	33 to 34	Negative
85709856	good	3AA	33 to 34	Negative
20102638	poor	2BB	31 to 32	Negative
35183227	poor	2BB-	30 or younger	Positive
61311777	poor	3BB-	30 or younger	Positive
57298520	good	3AB	35 to 36	Positive
59609632	good	3AB	33 to 34	Positive
66066274	good	3BB	35 to 36	Positive
66832653	poor	3B-B	35 to 36	Positive
88939297	good	3AB	30 or younger	Positive
91385919	good	3AB	33 to 34	Positive
26278116	good	3BB	35 to 36	Positive
14302022	poor	3BB-	35 to 36	Negative
86176576	good	3A-A-	35 to 36	Negative
95734805	poor	3A-B	30 or younger	Positive
72625604	good	3A-B	30 or younger	Negative
84315440	poor	1BB	31 to 32	Positive
20430073	poor	3B-B	37 to 38	Positive
52572430	good	3AA-	37 to 38	Positive
83192950	good	3BB	30 or younger	Positive
75017808	good	3A-A-	30 or younger	Positive
50158937	good	3A-A-	30 or younger	Positive
76161217	good	3A-B	39 to 40	Negative
10423352	good	3AA-	33 to 34	Negative
93268410	good	3A-B	33 to 34	Positive
98239149	good	3AB	30 or younger	Negative
65601225	good	3BB	30 or younger	Positive

58508976	good	3A-A-	41 or older	Negative
47041072	poor	2BB	41 or older	Negative
31565190	good	3A-A	37 to 38	Positive
91715247	poor	1BB	33 to 34	Negative
95802258	good	3AA	30 or younger	Positive
11139600	good	3AA	30 or younger	Negative
42669672	good	3A-A-	35 to 36	Positive
50936296	good	3AA	30 or younger	Positive
66036836	poor	1.5BB	41 or older	Negative
10239739	good	3AA	37 to 38	Positive
12997501	good	3A-A-	39 to 40	Positive
91493198	poor	2B-B-/C	39 to 40	Negative
45445337	good	3BB	41 or older	Positive
53929398	good	3A-A-	41 or older	Positive
23400612	good	3AA	31 to 32	Positive
47546499	good	3A-B	30 or younger	Positive
54740457	poor	3BB-	37 to 38	Negative
22786566	good	3AA-	33 to 34	Positive
76615559	good	3AA-	31 to 32	Positive
75682442	good	3A-B	30 or younger	Positive
47088599	poor	3BC	41 or older	Negative
15489437	poor	1B-B-	41 or older	Negative
30857998	good	3A-B	33 to 34	Positive
55092056	good	3AA-	33 to 34	Positive
73742859	good	3AA	33 to 34	Positive
78074627	good	3AA	35 to 36	Positive
99827445	good	3AB	41 or older	Negative
69410814	good	3BB	31 to 32	Negative
30222448	poor	2BB	31 to 32	Negative
34991057	good	3BB	31 to 32	Negative
44325360	good	3AB	39 to 40	Negative
54300779	poor	2BB	39 to 40	Negative
98027925	good	3BB	39 to 40	Positive
76892213	poor	3BB-	30 or younger	Positive
52831834	good	3A-B	30 or younger	Negative
11701995	poor	3B-B	30 or younger	Positive
50522015	good	3A-A-	31 to 32	Positive
78909238	poor	3BB-	37 to 38	Positive
70585947	good	3A-A-	37 to 38	Negative
66028352	good	3BB	37 to 38	Negative
84454083	good	3A-B	39 to 40	Positive
68416801	poor	2BB	31 to 32	Positive

40871077	poor	1.5B-B	31 to 32	Positive
56940400	good	3BA-	31 to 32	Negative
89672220	poor	2B-B	31 to 32	Negative
34128462	good	3AB	30 or younger	Positive
35583292	good	3BB	35 to 36	Positive
47891099	good	3BB	35 to 36	Positive
18981889	good	3BB	39 to 40	Negative
90134596	poor	1BB-	39 to 40	Negative
27574436	good	3A-B	37 to 38	Positive
60827399	good	3BB	37 to 38	Positive
63501625	poor	3BB	31 to 32	Negative
56066139	good	3A-A-	33 to 34	Negative
30782965	good	3A-A-	33 to 34	Negative
53297449	good	3A-B	33 to 34	Positive
72067898	good	4AA-	31 to 32	Negative
88579541	good	3A-B	31 to 32	Positive
30617740	good	3BB	30 or younger	Negative
11431853	poor	3B-B	30 or younger	Negative
98789883	good	3BB	31 to 32	Positive
78103016	good	3AB	33 to 34	Positive
42934507	good	3BB	33 to 34	Positive
53593142	good	3BB	30 or younger	Positive
91752750	poor	3B-B-	33 to 34	Negative
75458478	poor	3BB-	33 to 34	Negative
90574063	poor	3BB	37 to 38	Positive
16621319	good	3A-A-	37 to 38	Negative
22269702	good	3BA-	30 or younger	Negative
53886233	good	3A-B	30 or younger	Positive
32025691	good	3BB	35 to 36	Positive
55240342	good	3A-B	35 to 36	Negative
11132559	good	3A-A-	31 to 32	Positive
40168641	poor	3BB	31 to 32	Negative
63049974	poor	2BB	31 to 32	Negative
58694444	poor	1BB	31 to 32	Negative
65024159	good	3A-A-	33 to 34	Positive
39927026	good	3BA-	35 to 36	Positive
95121387	good	3BA-	35 to 36	Positive
11088653	poor	3A-B	33 to 34	Negative
85777973	poor	1BB	33 to 34	Negative
60706312	good	3A-B	35 to 36	Positive
34344304	good	3A-B	37 to 38	Negative
33152844	good	3A-A-	41 or older	Positive

24956618	good	3A-B	37 to 38	Positive
55067032	good	3A-A-	37 to 38	Positive
75412161	good	4AA	33 to 34	Positive
56838626	poor	2BB	31 to 32	Negative
15547543	poor	2B-B-	31 to 32	Negative
66220363	good	3A-A-	33 to 34	Negative
75307492	good	3AA-	33 to 34	Negative
22518496	good	3BB	33 to 34	Positive
46545499	poor	3B-B	31 to 32	Positive
96981597	good	3A-A-	30 or younger	Positive
60909236	poor	1BB-	31 to 32	Negative
34863032	poor	3BB	37 to 38	Positive
27323957	poor	3B-B-	37 to 38	Negative
91657493	poor	3B-B-	37 to 38	Negative
68828672	poor	2B-B-	37 to 38	Negative
72536892	poor	1BB	37 to 38	Negative
34856423	good	3BB	35 to 36	Positive
78921464	good	3A-B	35 to 36	Negative
30058331	good	3A-A-	33 to 34	Positive
79370960	good	3A-A-	33 to 34	Positive
46656783	good	3A-B	35 to 36	Negative
22889471	good	3AB	30 or younger	Positive
33756944	poor	3B-B-	30 or younger	Positive
33654296	good	3A-B	30 or younger	Positive
81306342	poor	2BB	30 or younger	Positive
54101881	poor	3B-B-	41 or older	Negative
89377439	good	3BB	30 or younger	Positive
68376307	good	3A-A-	33 to 34	Positive
57262611	good	3AA-	33 to 34	Negative
13198789	good	3AA	30 or younger	Positive
46831040	poor	2BB	33 to 34	Negative
73838180	good	3AA-	30 or younger	Positive
45661535	good	3AA	31 to 32	Positive
14551441	poor	1BB-	41 or older	Negative
21330364	poor	1BB-	41 or older	Negative
17585937	good	3AA-	30 or younger	Positive
15347170	good	3A-B	31 to 32	Positive
63134776	good	3A-B	31 to 32	Positive
96311248	poor	3BB	41 or older	Positive
60483902	poor	3A-B	31 to 32	Positive
38069751	good	3A-A-	37 to 38	Negative
51109109	good	3BA-	37 to 38	Negative

16303512	good	3A-B	31 to 32	Negative
44361437	poor	3BB-	33 to 34	Negative
71012830	poor	1BB	33 to 34	Negative
38590018	good	3BB	33 to 34	Negative
31657741	poor	3B-B	31 to 32	Positive
10794293	good	5AA-	30 or younger	Positive
25918541	good	3BB	35 to 36	Positive
36050207	poor	3BB-	35 to 36	Positive
23418850	poor	3BB	31 to 32	Negative
36054645	poor	3BB-	31 to 32	Positive
92607283	good	3BB	31 to 32	Negative
89284746	good	3A-A-	33 to 34	Positive
68125925	good	3A-B	35 to 36	Negative
33769223	poor	3B-B	30 or younger	Positive
59009135	good	3A-B	30 or younger	Positive
33595073	good	3AB	33 to 34	Positive
35947526	poor	2BB	33 to 34	Positive
54858552	good	3BB	31 to 32	Positive
97857206	good	3AA	37 to 38	Positive
30413814	good	3A-B	33 to 34	Positive
64917559	good	3A-B	41 or older	Negative
38646889	good	3A-B	41 or older	Positive
42833157	good	3A-B	35 to 36	Negative
11355843	good	3BB	33 to 34	Positive
41143942	good	3A-A-	30 or younger	Positive
89226327	good	3BB	31 to 32	Negative
41305043	good	3BB	31 to 32	Positive
87251887	poor	3BB	35 to 36	Positive
26921424	good	3A-B	35 to 36	Negative
39385732	good	3A-B	33 to 34	Positive
79688012	good	3A-A-	37 to 38	Negative
91829450	good	3A-B	37 to 38	Negative
40590233	good	3AB	37 to 38	Negative
17017003	good	3A-A-	30 or younger	Positive
69390122	poor	1BB	35 to 36	Negative
26457399	good	3AA	31 to 32	Positive
41537951	good	3A-A-	35 to 36	Positive
75376401	good	3A-B	37 to 38	Positive
68938160	good	3A-B	33 to 34	Negative
20505988	good	3A-B	31 to 32	Positive
53689334	good	3A-B	33 to 34	Positive
18707760	good	3AA	30 or younger	Positive

99796531	good	3AA-	30 or younger	Positive
13465060	good	3A-A-	30 or younger	Positive
51674206	good	3A-A-	33 to 34	Positive
34506601	good	3A-B	31 to 32	Positive
14411240	good	3A-B	30 or younger	Negative
97344175	good	3A-B	33 to 34	Positive
23161440	good	3A-A-	31 to 32	Positive
29479505	poor	1BB	30 or younger	Positive
40038404	poor	1B-B	37 to 38	Negative
15598892	good	3BB	37 to 38	Negative
31617855	good	3A-A-	30 or younger	Positive
58398181	good	3A-B	30 or younger	Positive
43975915	good	3BB	33 to 34	Positive
72267704	poor	1B-B-	41 or older	Negative
66886907	good	3A-B	30 or younger	Negative
73090006	good	3BB	30 or younger	Positive
31681948	poor	3B-B	30 or younger	Positive
96274540	good	3BB	39 to 40	Positive
96246118	poor	3BB	39 to 40	Positive
14239476	good	3BB	33 to 34	Negative
45437591	good	3A-B	39 to 40	Negative
64241754	poor	3BB-	35 to 36	Negative
66021218	good	3AB	37 to 38	Negative
43010140	poor	2BB	37 to 38	Negative
23336664	good	3A-A	33 to 34	Positive
95521239	good	3A-A-	30 or younger	Positive
82275436	good	3A-B	33 to 34	Negative
80984534	good	3A-B	33 to 34	Positive
56901753	good	3A-A-	35 to 36	Positive
88307899	good	3BA-	31 to 32	Negative
56303970	good	3A-A-	31 to 32	Negative
80213367	good	3A-B	33 to 34	Positive
27715166	good	3BB	37 to 38	Negative
13117449	good	3A-A-	39 to 40	Positive
98720644	poor	3BB	39 to 40	Positive
23581318	good	3A-B	30 or younger	Positive
79622533	good	3AA-	31 to 32	Negative
55084529	good	3BB	31 to 32	Negative
60344109	good	3A-B	31 to 32	Negative
14025550	good	3BA-	41 or older	Negative
19113736	good	3A-B	41 or older	Negative
97890153	good	3AA	35 to 36	Positive

97576868	good	3BA-	39 to 40	Negative
48055549	poor	3BB	31 to 32	Negative
13729954	good	4AA	33 to 34	Positive
13838446	good	3A-B	39 to 40	Positive
18935832	good	3A-A-	39 to 40	Positive
77562445	good	3A-A-	35 to 36	Positive
20624042	poor	2BB	31 to 32	Negative
55878812	poor	2BB	35 to 36	Positive
11570017	poor	3BB	30 or younger	Negative
10867514	good	3BB	35 to 36	Positive
63329829	good	3A-B	33 to 34	Positive
56727066	good	3A-B	37 to 38	Positive
77286893	good	3A-B	30 or younger	Positive
31134263	good	3A-A-	31 to 32	Negative
21664440	poor	2.5BB-	31 to 32	Negative
68677180	poor	3BB	31 to 32	Negative
19805340	poor	2BB	35 to 36	Negative
47827979	poor	1BB	35 to 36	Negative
29960432	good	3BB	37 to 38	Positive
66593974	good	3BB	37 to 38	Positive
52988180	good	3AA	33 to 34	Positive
43308527	good	3AB	35 to 36	Positive
87703937	good	4AA	30 or younger	Positive
33000125	good	3BB	31 to 32	Negative
54255267	poor	3A-B	30 or younger	Negative
93286159	good	3AA	30 or younger	Negative
39627541	poor	3B-B-	35 to 36	Negative
46065511	good	3A-B	35 to 36	Positive
50197856	good	3A-B	35 to 36	Negative
24269596	poor	2BB	31 to 32	Positive
74584408	good	3A-A-	31 to 32	Positive
37157131	good	3A-B	33 to 34	Positive
67985398	good	3A-A-	41 or older	Positive
83467377	poor	2.5BB-	33 to 34	Positive
56107987	good	3A-B	35 to 36	Positive
21166337	good	3AA-	35 to 36	Negative
59845945	poor	1BB	31 to 32	Positive
33324428	good	3AA	33 to 34	Positive
84937659	poor	3BB-	39 to 40	Positive
77670904	poor	3BB	31 to 32	Negative
80678895	poor	1B-B-	37 to 38	Negative
57709578	poor	1.5BB	37 to 38	Negative

49469922	good	3A-A-	37 to 38	Negative
81033173	poor	1.5BB	37 to 38	Negative
80617951	poor	3BB	37 to 38	Negative
72968743	good	3A-B	35 to 36	Positive
26407306	good	3A-A-	35 to 36	Negative
54818772	good	3BA-	35 to 36	Negative
68089424	poor	1.5BB	39 to 40	Positive
41926579	good	3AA-	37 to 38	Positive
99316711	good	3A-B	35 to 36	Positive
39761569	poor	1BB	33 to 34	Negative
38707883	good	3BB	31 to 32	Positive
42073997	poor	3BB-	31 to 32	Positive
54404403	good	3BB	39 to 40	Negative
80093996	poor	3BB	39 to 40	Negative
30310345	poor	3BB-	39 to 40	Negative
30189374	good	3A-B	35 to 36	Negative
93832914	good	3A-B	30 or younger	Positive
79323033	good	3A-B	37 to 38	Positive
17474956	good	3AB	39 to 40	Positive
77007598	good	3A-A-	39 to 40	Positive
29215347	good	3A-A-	31 to 32	Positive
80313188	good	3A-A-	31 to 32	Positive
81593484	poor	2.5B-B-	35 to 36	Positive
36429347	good	3A-B	35 to 36	Positive
41362367	good	3BB	33 to 34	Positive
32176207	poor	2BB-	37 to 38	Negative
45722195	good	3A-B	37 to 38	Positive
39558724	poor	3BB-/C	33 to 34	Negative
69829405	good	3A-B	33 to 34	Negative
15088065	good	3A-A-	35 to 36	Positive
53064676	good	3BB	35 to 36	Positive
80203541	poor	3A-B	33 to 34	Positive
99356908	good	3A-B	30 or younger	Positive
45485270	good	3BB	30 or younger	Positive
44137049	good	3A-B	31 to 32	Positive
51244115	good	3A-B	31 to 32	Positive
11142866	good	3A-A-	30 or younger	Positive
72973453	poor	3B-B	30 or younger	Positive
32856722	good	3A-A-	35 to 36	Negative
89957882	poor	1B-B	35 to 36	Negative
57725925	poor	2BB	35 to 36	Negative
99783224	good	3A-B	37 to 38	Positive

39119089	good	3A-B	39 to 40	Positive
23542928	good	3A-B	35 to 36	Negative
73179601	good	3BB	35 to 36	Positive
13718777	good	3A-B	35 to 36	Positive
32885354	good	3BB	33 to 34	Positive
76634392	poor	3BB	31 to 32	Negative
51223586	poor	2BB	41 or older	Negative
38855285	poor	3BB	39 to 40	Negative
50898861	good	3BB	39 to 40	Positive
57765820	poor	3bb-	33 to 34	Negative
56142103	good	3BB	37 to 38	Positive
18872022	good	3BB	37 to 38	Positive
10862851	good	3A-B	39 to 40	Positive
17340290	good	3A-A-	39 to 40	Positive
41160841	poor	2BB	30 or younger	Positive
20588100	poor	1B-B-	41 or older	Negative
39370153	poor	1BB	41 or older	Negative
63100139	poor	1BB	41 or older	Negative
36464521	good	3AA-	35 to 36	Positive
94499537	good	3AA	31 to 32	Positive
29443389	poor	3B-B	33 to 34	Positive
40479925	good	3bb	33 to 34	Negative
18781105	good	3AA	33 to 34	Positive
79422643	good	3A-A-	30 or younger	Positive
68550838	good	3A-A-	31 to 32	Positive
14411543	good	3AA-	30 or younger	Positive
55812838	good	3A-B	41 or older	Negative
30573434	good	3BB	37 to 38	Positive
78496081	good	3AB	33 to 34	Positive
42613115	poor	3BB	33 to 34	Negative
95160595	good	3A-B	30 or younger	Positive
57041764	good	3AB	30 or younger	Positive
47432090	good	3BB	37 to 38	Negative
99445735	good	3AA	31 to 32	Negative
50097770	good	3A-A-	37 to 38	Positive
38829662	poor	3BB	41 or older	Negative
26185318	poor	2BB	41 or older	Negative
95226858	poor	3BB	35 to 36	Positive
91447446	good	3A-A-	31 to 32	Positive
25315588	good	3A-B	33 to 34	Negative
93485380	good	3A-B	37 to 38	Negative
19401438	good	3BB	31 to 32	Negative

81088305	poor	3BB-	31 to 32	Negative
86809997	good	3A-B	39 to 40	Positive
46329298	good	3A-B	39 to 40	Negative
28214327	good	3BB	33 to 34	Positive
19373130	good	3AB	37 to 38	Negative
47398252	good	3A-B	39 to 40	Positive
97393443	good	3BB	35 to 36	Positive
94131148	good	3AA	30 or younger	Positive
41873785	good	3A-A-	31 to 32	Positive
58845039	poor	1BB	33 to 34	Negative
68428053	poor	3BB-	39 to 40	Negative
48186769	good	3BB	39 to 40	Negative
98529045	poor	2BB-/C	39 to 40	Positive
87507048	poor	3BA-	35 to 36	Positive
88146076	good	3A-A-	30 or younger	Positive
43133885	good	3A-A-	33 to 34	Positive
60973558	good	3A-A-	31 to 32	Positive
56194785	good	4AA	31 to 32	Negative
57833451	good	3BB	30 or younger	Positive
85748422	good	3AA	30 or younger	Positive
87180394	good	3AA	30 or younger	Positive
22859437	poor	3BB-	33 to 34	Positive
12688156	poor	3A-B	35 to 36	Positive
54135276	good	3BB	37 to 38	Negative
61236518	good	3A-A-	33 to 34	Negative
47076516	poor	3A-B	35 to 36	Positive
35844723	good	3AA-	37 to 38	Positive
44408119	good	3A-A-	41 or older	Positive
22272191	poor	2B-B	33 to 34	Negative
40178292	good	3A-B	33 to 34	Positive
89860445	good	3BB	35 to 36	Negative
15631749	good	3A-B	35 to 36	Positive
40815275	good	3A-B	37 to 38	Positive
13284489	good	3A-B	30 or younger	Negative
45351144	poor	2BB	35 to 36	Positive
31071070	good	3A-A-	39 to 40	Positive
39866553	good	3BB	35 to 36	Negative
47811395	good	3A-B	33 to 34	Positive
68030192	good	3AB	35 to 36	Negative
71549446	good	3A-B	30 or younger	Positive
64327856	poor	3BB-	41 or older	Negative
47132671	good	3A-A-	33 to 34	Positive

92356667	poor	2.5B-B	37 to 38	Negative
28184213	good	3A-B	37 to 38	Positive
43653546	good	3BB	39 to 40	Negative
27926063	good	3A-B	39 to 40	Negative
13101335	good	3BB	31 to 32	Positive
83990350	poor	1B-B-	41 or older	Negative
15868254	good	3BB	41 or older	Negative
88024939	good	3BB	37 to 38	Negative
85909775	good	3A-A-	31 to 32	Negative
48666957	good	3A-B	33 to 34	Negative
73796234	good	3A-B	31 to 32	Negative
43999399	good	3BB	33 to 34	Positive
50696461	good	3A-B	39 to 40	Negative
95489580	poor	3BB-	33 to 34	Negative
87755745	good	3A-A-	33 to 34	Positive
81978172	good	3A-A	35 to 36	Positive
72619414	good	3A-B	37 to 38	Positive
86521545	good	3A-A-	37 to 38	Negative
48725685	good	3A-A-	37 to 38	Negative
93169721	good	3A-A-	31 to 32	Positive
22853905	poor	3BB	41 or older	Negative
92374377	good	3A-A-	41 or older	Negative
11075821	poor	3A-B	31 to 32	Positive
95892426	good	3A-B	39 to 40	Positive
57511259	good	3BB	39 to 40	Positive
32390208	good	3A-B	30 or younger	Negative
29850098	poor	3B-B-	37 to 38	Positive
87945558	good	3A-B	37 to 38	Positive
35959611	good	3A-B	31 to 32	Positive
15921159	poor	2B-B-	41 or older	Negative
72797188	good	3A-B	41 or older	Negative
82384146	poor	1.5BB	39 to 40	Negative
53800066	poor	1BB	39 to 40	Negative
18079557	poor	3BB-/C	31 to 32	Negative
56763520	poor	1BB	35 to 36	Negative
87885091	poor	2BB	35 to 36	Negative
54402234	poor	1B-B-	39 to 40	Negative
36264590	good	3A-B	35 to 36	Negative
74354682	poor	3A-B	35 to 36	Negative
91075772	poor	3A-B	37 to 38	Negative
91404880	good	3A-B	30 or younger	Negative

Embryos	E1	E2	E3	E4	E5	Majority-vote	STORK
a12	-1	-1	-1	1	-1	-1	1
a14	0	1	1	0	-1	0	1
a15	-1	-1	-1	-1	-1	-1	1
a20	0	0	-1	0	-1	0	1
a23	0	0	-1	-1	0	0	1
a25	0	0	-1	-1	-1	-1	1
a26	1	0	0	1	0	0	1
a27	1	1	0	1	1	1	1
a28	0	-1	-1	0	0	0	1
a29	0	0	0	1	0	0	1
a3	-1	-1	-1	0	-1	-1	1
a30	0	0	0	1	1	0	1
a31	0	0	-1	0	0	0	1
a37	1	0	1	1	1	1	1
a39	0	0	1	1	1	1	1
a4	0	0	0	0	0	0	1
a45	1	0	1	1	1	1	1
a46	0	0	0	-1	0	0	1
a50	0	1	1	0	-1	0	1
a51	0	0	-1	-1	-1	-1	1
a52	0	0	-1	-1	-1	-1	1
a56	0	0	-1	0	0	0	1
a68	0	0	-1	0	0	0	1
a69	0	0	0	0	0	0	1
a73	0	0	0	1	-1	0	1
a75	0	1	-1	1	-1	0	1
a76	0	0	-1	-1	-1	-1	1
a77	0	0	0	-1	0	0	1
a8	1	0	0	1	1	1	1
a81	0	1	0	1	1	1	1
a82	1	1	1	1	1	1	1
a84	1	0	0	1	0	0	1
a90	1	0	0	0	0	0	1
a94	0	1	0	1	1	1	1
a95	0	0	0	-1	0	0	1
ab1	0	0	-1	0	0	0	1
ab13	0	0	-1	-1	0	0	1
ab19	0	0	0	-1	-1	0	1
ab21	0	0	0	1	1	0	1
ab28	1	0	0	0	0	0	1
ab29	0	0	-1	1	1	0	1
ab34	0	0	0	0	-1	0	1
ab38	-1	0	-1	-1	-1	-1	1
ab47	0	0	0	1	0	0	1

number 1=good
number -1=poor
number 0=fair or uncertain
E1=Embroyologist 1
E2=Embroyologist 2
E3=Embroyologist 3
E4=Embroyologist 4
E5=Embroyologist 5

ab52	0	0	-1	0	0	0	1
ab66	1	1	1	1	0	1	1
ab67	0	0	-1	0	0	0	1
ab77	1	-1	0	0	1	0	1
ab80	1	0	0	1	-1	0	1
ab81	-1	0	-1	1	-1	-1	1
ab82	0	0	0	0	1	0	1
ab84	1	0	0	1	1	1	1
ab89	0	0	-1	-1	0	0	1
ab9	1	1	0	1	0	1	1
ab91	0	0	-1	0	0	0	1
ab92	1	0	0	1	0	0	1
ab95	0	-1	-1	0	0	0	1
ab96	0	-1	0	0	0	0	1
ac10	0	0	-1	0	-1	0	1
ac13	0	-1	-1	-1	-1	-1	1
ac15	0	0	-1	-1	-1	-1	1
ac22	0	-1	-1	1	0	0	1
ac30	-1	0	-1	0	-1	-1	1
ac34	0	-1	-1	1	0	0	1
ac38	0	0	-1	0	0	0	1
ac4	0	0	0	0	0	0	1
ac40	0	0	-1	-1	0	0	1
ac47	-1	0	-1	0	-1	-1	1
ac50	1	1	1	1	1	1	1
ac55	0	0	-1	1	-1	0	1
ac58	0	0	0	1	0	0	1
ac64	1	1	1	1	1	1	1
ac67	0	1	0	0	0	0	1
ac68	1	1	1	1	1	1	1
ac8	0	0	-1	1	0	0	1
ad17	1	0	0	0	1	0	1
ad18	1	0	0	1	1	1	1
ad21	1	0	0	1	1	1	1
ad30	-1	-1	-1	0	-1	-1	1
ad34	1	1	0	1	1	1	1
ad35	0	1	-1	1	1	1	1
ad38	1	0	-1	1	1	1	1
ad41	0	0	-1	-1	0	0	1
ad7	1	0	-1	1	0	0	1
ad9	0	0	0	1	0	0	1
ae10	0	0	-1	0	0	0	1
ae15	0	0	-1	0	0	0	1
ae19	0	1	1	1	1	1	1
ae22	0	0	0	0	0	0	1

ae24	0	0	0	0	0	0	1
ae25	1	1	1	1	1	1	1
ae27	1	0	0	1	-1	0	1
ae29	1	1	1	1	0	1	1
ae36	0	0	-1	0	-1	0	1
ae37	0	1	0	1	-1	0	1
af11	0	0	-1	-1	0	0	1
af12	1	0	0	1	1	1	1
af13	0	0	0	0	0	0	1
af14	0	0	-1	1	0	0	1
af16	0	0	-1	1	0	0	1
af2	0	0	0	1	0	0	1
af23	0	1	-1	1	0	0	1
af26	0	0	-1	1	0	0	1
af3	1	1	0	1	1	1	1
af30	0	1	-1	1	0	0	1
af35	1	1	0	1	0	1	1
af4	-1	-1	-1	0	-1	-1	1
af7	0	0	-1	-1	-1	-1	1
a1	-1	-1	-1	-1	0	-1	-1
a10	0	0	0	1	0	0	-1
a11	-1	-1	-1	0	-1	-1	-1
a13	-1	-1	-1	-1	-1	-1	-1
a16	0	-1	-1	-1	-1	-1	-1
a17	0	0	0	0	0	0	-1
a18	0	-1	-1	-1	-1	-1	-1
a19	-1	-1	-1	-1	-1	-1	-1
a2	-1	-1	-1	-1	-1	-1	-1
a21	0	0	0	1	0	0	-1
a22	-1	-1	-1	-1	-1	-1	-1
a24	-1	-1	-1	-1	-1	-1	-1
a32	-1	-1	-1	-1	-1	-1	-1
a33	1	-1	-1	1	1	1	-1
a34	-1	-1	-1	-1	-1	-1	-1
a35	0	0	0	1	-1	0	-1
a36	-1	-1	-1	-1	-1	-1	-1
a38	0	1	-1	0	0	0	-1
a40	0	-1	-1	-1	-1	-1	-1
a41	0	0	0	0	-1	0	-1
a42	-1	-1	-1	-1	-1	-1	-1
a43	0	-1	-1	0	-1	-1	-1
a44	-1	0	-1	-1	-1	-1	-1
a47	0	-1	-1	-1	-1	-1	-1
a48	-1	0	-1	-1	-1	-1	-1
a49	-1	0	0	-1	-1	-1	-1

a5	0	1	-1	0	0	0	-1
a53	-1	-1	-1	0	-1	-1	-1
a54	-1	0	-1	0	-1	-1	-1
a55	-1	0	-1	-1	-1	-1	-1
a57	-1	-1	-1	-1	-1	-1	-1
a58	0	0	-1	-1	-1	-1	-1
a59	-1	0	-1	-1	-1	-1	-1
a6	0	-1	-1	-1	0	-1	-1
a60	-1	-1	-1	-1	-1	-1	-1
a61	0	-1	-1	-1	-1	-1	-1
a62	0	-1	-1	0	-1	-1	-1
a63	0	0	0	0	0	0	-1
a64	-1	0	-1	0	-1	-1	-1
a65	-1	-1	-1	-1	-1	-1	-1
a66	0	0	-1	-1	-1	-1	-1
a67	-1	-1	-1	-1	-1	-1	-1
a7	0	0	-1	0	0	0	-1
a70	-1	0	0	0	-1	0	-1
a71	0	0	-1	0	-1	0	-1
a72	1	1	-1	1	0	1	-1
a74	1	-1	-1	0	-1	-1	-1
a78	-1	-1	-1	0	-1	-1	-1
a79	0	-1	-1	-1	-1	-1	-1
a80	1	1	0	-1	-1	0	-1
a83	-1	0	-1	-1	-1	-1	-1
a85	-1	-1	-1	-1	-1	-1	-1
a86	0	0	0	-1	-1	0	-1
a87	-1	0	-1	-1	-1	-1	-1
a88	-1	-1	-1	-1	-1	-1	-1
a89	1	0	0	0	0	0	-1
a9	0	0	0	-1	1	0	-1
a91	0	0	-1	0	-1	0	-1
a92	0	0	-1	1	1	0	-1
a93	0	-1	-1	0	-1	-1	-1
ab10	0	-1	-1	1	0	0	-1
ab100	-1	-1	-1	-1	-1	-1	-1
ab11	-1	-1	-1	-1	-1	-1	-1
ab12	0	0	0	-1	-1	0	-1
ab14	0	0	0	-1	-1	0	-1
ab15	0	0	-1	-1	-1	-1	-1
ab16	-1	0	-1	-1	-1	-1	-1
ab17	0	0	-1	-1	-1	-1	-1
ab18	-1	0	-1	-1	-1	-1	-1
ab2	-1	-1	-1	-1	-1	-1	-1
ab20	-1	-1	-1	-1	-1	-1	-1

ab22	0	0	-1	1	0	0	-1
ab23	-1	-1	-1	-1	-1	-1	-1
ab24	-1	-1	-1	-1	-1	-1	-1
ab25	0	0	-1	-1	-1	-1	-1
ab26	-1	0	-1	-1	-1	-1	-1
ab27	-1	-1	-1	-1	-1	-1	-1
ab3	-1	0	-1	-1	-1	-1	-1
ab30	0	-1	-1	-1	-1	-1	-1
ab31	-1	0	-1	-1	-1	-1	-1
ab32	0	0	-1	1	0	0	-1
ab33	0	0	0	-1	-1	0	-1
ab35	-1	0	-1	-1	-1	-1	-1
ab36	-1	-1	-1	-1	-1	-1	-1
ab37	-1	0	-1	-1	-1	-1	-1
ab39	-1	-1	-1	-1	-1	-1	-1
ab4	0	-1	0	0	0	0	-1
ab40	-1	-1	-1	-1	-1	-1	-1
ab41	-1	-1	-1	-1	-1	-1	-1
ab42	-1	-1	-1	-1	-1	-1	-1
ab43	-1	-1	-1	-1	-1	-1	-1
ab44	-1	0	-1	-1	-1	-1	-1
ab45	0	0	0	-1	-1	0	-1
ab46	-1	-1	-1	-1	-1	-1	-1
ab48	0	0	0	-1	0	0	-1
ab49	0	0	0	0	-1	0	-1
ab5	1	1	-1	0	1	1	-1
ab50	0	0	-1	-1	0	0	-1
ab51	0	-1	-1	-1	-1	-1	-1
ab53	0	-1	0	-1	-1	-1	-1
ab54	0	0	0	-1	-1	0	-1
ab55	-1	0	-1	-1	-1	-1	-1
ab56	0	-1	-1	-1	-1	-1	-1
ab57	-1	-1	-1	-1	-1	-1	-1
ab58	0	0	0	-1	-1	0	-1
ab59	0	-1	-1	-1	-1	-1	-1
ab6	-1	0	-1	-1	-1	-1	-1
ab60	-1	-1	-1	-1	-1	-1	-1
ab61	0	0	-1	0	-1	0	-1
ab62	-1	0	-1	-1	-1	-1	-1
ab63	-1	-1	-1	-1	-1	-1	-1
ab64	0	0	-1	-1	0	0	-1
ab65	0	0	0	-1	-1	0	-1
ab68	0	-1	-1	-1	-1	-1	-1
ab69	0	0	0	-1	-1	0	-1
ab7	-1	-1	-1	-1	-1	-1	-1

ab70	-1	-1	-1	-1	-1	-1	-1
ab71	0	0	-1	-1	-1	-1	-1
ab72	0	0	0	-1	-1	0	-1
ab73	0	-1	-1	-1	-1	-1	-1
ab74	-1	0	-1	-1	-1	-1	-1
ab75	0	-1	-1	0	-1	-1	-1
ab76	0	0	-1	1	0	0	-1
ab78	-1	0	-1	-1	-1	-1	-1
ab79	0	0	0	-1	-1	0	-1
ab8	-1	0	-1	-1	-1	-1	-1
ab83	0	-1	-1	-1	-1	-1	-1
ab85	0	-1	-1	-1	0	-1	-1
ab86	-1	-1	-1	-1	-1	-1	-1
ab87	-1	-1	-1	-1	-1	-1	-1
ab88	0	0	0	-1	-1	0	-1
ab90	-1	0	-1	0	-1	-1	-1
ab93	-1	-1	-1	0	-1	-1	-1
ab94	0	0	0	1	-1	0	-1
ab97	0	-1	-1	-1	-1	-1	-1
ab98	-1	-1	-1	-1	-1	-1	-1
ab99	0	0	0	-1	-1	0	-1
ac1	0	0	0	-1	-1	0	-1
ac11	0	-1	-1	-1	-1	-1	-1
ac12	-1	-1	-1	-1	-1	-1	-1
ac14	0	0	-1	1	0	0	-1
ac16	0	0	0	-1	-1	0	-1
ac17	0	0	0	-1	-1	0	-1
ac18	-1	0	-1	-1	-1	-1	-1
ac19	0	0	0	-1	-1	0	-1
ac2	-1	0	-1	-1	-1	-1	-1
ac20	-1	-1	-1	-1	-1	-1	-1
ac21	0	1	1	1	0	1	-1
ac23	0	0	-1	-1	-1	-1	-1
ac24	-1	-1	-1	-1	-1	-1	-1
ac25	0	-1	-1	-1	-1	-1	-1
ac26	-1	0	-1	-1	-1	-1	-1
ac27	-1	-1	-1	-1	-1	-1	-1
ac28	0	-1	-1	-1	-1	-1	-1
ac29	0	0	-1	-1	-1	-1	-1
ac3	0	0	-1	-1	-1	-1	-1
ac31	0	-1	-1	-1	-1	-1	-1
ac32	0	-1	-1	-1	-1	-1	-1
ac33	-1	0	-1	-1	-1	-1	-1
ac35	0	-1	-1	0	-1	-1	-1
ac36	-1	0	0	-1	-1	-1	-1

ac37	-1	-1	-1	-1	-1	-1	-1
ac39	0	0	-1	0	-1	0	-1
ac41	-1	0	-1	-1	-1	-1	-1
ac42	0	-1	-1	-1	-1	-1	-1
ac43	0	-1	-1	-1	-1	-1	-1
ac44	0	-1	-1	-1	-1	-1	-1
ac45	-1	-1	-1	-1	-1	-1	-1
ac46	0	1	-1	-1	-1	-1	-1
ac48	-1	-1	-1	-1	-1	-1	-1
ac49	-1	0	-1	-1	0	-1	-1
ac5	1	0	-1	0	-1	0	-1
ac51	0	0	0	-1	-1	0	-1
ac52	-1	0	-1	-1	-1	-1	-1
ac53	0	0	0	-1	-1	0	-1
ac54	0	0	0	0	0	0	-1
ac56	0	-1	-1	-1	-1	-1	-1
ac57	0	-1	0	0	-1	0	-1
ac59	0	-1	-1	0	-1	-1	-1
ac6	-1	-1	-1	-1	-1	-1	-1
ac60	0	-1	-1	0	-1	-1	-1
ac61	0	-1	-1	0	-1	-1	-1
ac62	-1	-1	-1	-1	-1	-1	-1
ac63	-1	-1	-1	0	-1	-1	-1
ac65	1	1	1	1	-1	1	-1
ac66	0	1	0	0	-1	0	-1
ac7	0	-1	-1	-1	-1	-1	-1
ac9	-1	-1	-1	-1	-1	-1	-1
ad1	-1	-1	-1	-1	-1	-1	-1
ad10	0	0	-1	-1	1	0	-1
ad11	0	0	-1	0	-1	0	-1
ad12	0	0	0	1	-1	0	-1
ad13	-1	-1	-1	-1	-1	-1	-1
ad14	-1	-1	-1	0	-1	-1	-1
ad15	0	0	-1	0	-1	0	-1
ad16	-1	-1	-1	-1	-1	-1	-1
ad19	-1	0	-1	-1	-1	-1	-1
ad2	-1	-1	-1	-1	-1	-1	-1
ad20	-1	-1	-1	-1	-1	-1	-1
ad22	0	0	0	-1	-1	0	-1
ad23	0	0	-1	-1	-1	-1	-1
ad24	-1	0	-1	-1	-1	-1	-1
ad25	-1	-1	-1	-1	-1	-1	-1
ad26	0	0	0	-1	-1	0	-1
ad27	0	-1	-1	-1	-1	-1	-1
ad28	-1	-1	-1	-1	-1	-1	-1

ad29	0	0	0	-1	-1	0	-1
ad3	-1	-1	-1	-1	-1	-1	-1
ad31	0	0	0	0	-1	0	-1
ad32	0	-1	-1	-1	-1	-1	-1
ad33	0	0	1	-1	-1	0	-1
ad36	-1	-1	-1	-1	-1	-1	-1
ad37	-1	-1	0	-1	-1	-1	-1
ad39	-1	-1	-1	-1	-1	-1	-1
ad4	0	0	0	-1	-1	0	-1
ad40	0	0	0	-1	-1	0	-1
ad42	-1	0	-1	-1	-1	-1	-1
ad43	0	-1	-1	0	-1	-1	-1
ad44	0	-1	-1	-1	-1	-1	-1
ad45	-1	-1	-1	-1	-1	-1	-1
ad46	0	-1	-1	-1	-1	-1	-1
ad47	-1	0	-1	-1	-1	-1	-1
ad48	-1	-1	-1	-1	-1	-1	-1
ad5	0	0	-1	0	-1	0	-1
ad6	-1	-1	-1	-1	-1	-1	-1
ad8	0	-1	-1	-1	-1	-1	-1
ae1	-1	-1	-1	0	0	-1	-1
ae11	-1	-1	-1	-1	-1	-1	-1
ae12	0	-1	-1	0	-1	-1	-1
ae13	0	-1	-1	0	-1	-1	-1
ae14	0	0	-1	-1	0	0	-1
ae16	-1	-1	-1	-1	-1	-1	-1
ae17	-1	0	-1	-1	-1	-1	-1
ae18	0	-1	-1	-1	-1	-1	-1
ae2	-1	-1	-1	0	-1	-1	-1
ae20	-1	0	-1	-1	-1	-1	-1
ae21	0	0	0	-1	-1	0	-1
ae23	0	0	0	0	0	0	-1
ae26	-1	0	-1	-1	-1	-1	-1
ae28	-1	0	0	0	-1	0	-1
ae3	-1	-1	0	-1	-1	-1	-1
ae30	0	0	-1	0	-1	0	-1
ae31	-1	-1	-1	-1	-1	-1	-1
ae32	-1	-1	-1	-1	0	-1	-1
ae33	-1	-1	-1	-1	-1	-1	-1
ae34	-1	-1	-1	-1	-1	-1	-1
ae35	0	0	0	1	-1	0	-1
ae38	0	-1	-1	1	-1	-1	-1
ae39	0	-1	-1	0	-1	-1	-1
ae4	0	1	-1	1	1	0	-1
ae40	1	0	1	1	1	1	-1

ae41	0	1	-1	1	0	0	-1
ae42	-1	-1	-1	-1	0	-1	-1
ae43	0	0	0	-1	-1	0	-1
ae44	0	0	0	0	-1	0	-1
ae45	0	1	-1	1	0	0	-1
ae46	0	1	-1	1	-1	0	-1
ae47	0	0	-1	-1	0	0	-1
ae48	0	-1	-1	0	-1	-1	-1
ae5	0	0	0	0	-1	0	-1
ae6	-1	0	0	-1	-1	-1	-1
ae7	1	1	-1	1	0	1	-1
ae8	0	0	-1	0	-1	0	-1
ae9	0	0	0	0	-1	0	-1
af1	-1	0	-1	-1	-1	-1	-1
af10	-1	-1	-1	-1	-1	-1	-1
af15	-1	-1	-1	-1	-1	-1	-1
af17	0	0	0	-1	-1	0	-1
af18	0	1	-1	1	-1	0	-1
af19	-1	-1	-1	-1	-1	-1	-1
af20	-1	-1	-1	-1	-1	-1	-1
af21	-1	-1	-1	-1	-1	-1	-1
af22	-1	-1	-1	-1	-1	-1	-1
af24	0	0	0	-1	-1	0	-1
af25	-1	-1	-1	-1	-1	-1	-1
af27	0	0	0	-1	-1	0	-1
af28	0	0	-1	-1	0	0	-1
af29	0	-1	-1	-1	-1	-1	-1
af31	-1	0	0	0	-1	0	-1
af32	0	0	0	-1	-1	0	-1
af33	-1	-1	-1	-1	-1	-1	-1
af34	-1	-1	-1	-1	-1	-1	-1
af5	-1	0	-1	-1	-1	-1	-1
af6	0	0	0	-1	-1	0	-1
af8	-1	0	0	-1	-1	-1	-1
af9	-1	0	-1	-1	-1	-1	-1
